# New Antifungal Metabolites from the Mariana Trench Sediment-Associated Actinomycete *Streptomyces* sp. SY1965

**DOI:** 10.3390/md18080385

**Published:** 2020-07-24

**Authors:** Wenwen Yi, Le Qin, Xiao-Yuan Lian, Zhizhen Zhang

**Affiliations:** 1 Ocean College, Zhoushan Campus, Zhejiang University, Zhoushan 316021, China; 18790658213@163.com (W.Y.); qinle19951102@126.com (L.Q.); 2 College of Pharmaceutical Sciences, Zhejiang University, Hangzhou 310058, China

**Keywords:** Mariana Trench actinomycete, *Streptomyces* sp. SY1965, streptothiazolidine A, Diketopiperazines, antimicrobial activities

## Abstract

New streptothiazolidine A (**1**), streptodiketopiperazines A (**2**) and B (**3**), and (*S*)-1-(3-ethylphenyl)-1,2-ethanediol (**4**), together with eight known compounds (**5**–**12**), were isolated from the Mariana Trench sediment-associated actinomycete *Streptomyces* sp. SY1965. The racemic mixtures of (±)-streptodiketopiperazine (**2** and **3**) and (±)-1-(3-ethylphenyl)-1,2-ethanediol (**4** and **5**) were separated on a chiral high-performance liquid chromatography (HPLC) column. Structures of the new compounds were elucidated by their high-resolution electrospray ionization mass spectroscopy (HRESIMS) data and extensive nuclear magnetic resonance (NMR) spectroscopic analyses. Streptothiazolidine A is a novel salicylamide analogue with a unique thiazolidine-contained side chain and its absolute configuration was established by a combination of nuclear Overhauser effect spectroscopy (NOESY) experiment, electronic circular dichroism (ECD) and ^13^C NMR calculations. New streptothiazolidine A (**1**) and streptodiketopiperazines A (**2**) and B (**3**) showed antifungal activity against *Candida albicans* with MIC values of 47, 42, and 42 g/mL, respectively.

## 1. Introduction

The deep-sea organisms under extreme conditions have had to make significant biochemical and physiological adaptations for survival, which result in the modification of both gene regulation and metabolic pathways to produce metabolites with unique structures and bioactivities that differ from those produced by shallow-water organisms [1,2,3]. It was reported that about 75% of deep-sea natural products have biological activity, about 40% are drug-like, and 2/3 are within Known Drug Space (KDS) [2]. The well-known example is salinosporamide A, which was isolated from a marine obligate actinomycete *Salinospora tropica* strain CNB-392 [4]. Salinosporamide A is a second-generation proteasome inhibitor and currently termed as marizomib under investigation for the treatment of relapsed-refractory multiple myeloma and malignant glioma [5,6]. The marine obligate *Salinospora* actinomycetes were found in tropical and subtropical marine sediments at depths of up to 1100 m [7,8], and most of the secondary metabolites reported to date from the genus *Salinospora* are novel structural molecules [9].

**Figure 1:**
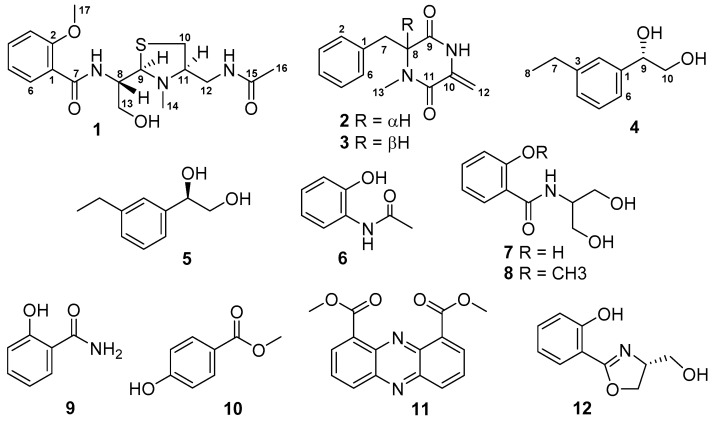
Structures of compounds **1**–**12** isolated from the culture of *Streptomyces* sp. SY1965.

The Mariana Trench is the deepest known site in the Earth’s oceans, reaching a depth of about 11,000 m at the Challenger Deep. Recent studies revealed that Mariana Trench sediments are enriched in microorganisms [10,11,12], however, the structures and bioactivities of their secondary metabolites are poorly known. During the course of our ongoing research program for the discovery of novel bioactive agents from marine microorganisms [13,14,15,16,17], an actinomycete strain SY1965 was isolated from a sediment sample collected from the Mariana Trench at depth of 11,000 m. An extract prepared from the culture of this hadal actinomycete in Gauze’s liquid medium with sea salt showed antiproliferative activities against human glioma U87MG and U251 cells with an inhibition rate of over 100%. Chemical investigation on this active extract resulted in the isolation of 12 secondary metabolites (**1**–**12**), including new streptothiazolidine A (**1**), streptodiketopiperazines A (**2**) and B (**3**), and (*S*)-1-(3-ethylphenyl)-1,2-ethanediol (**4**) (Figure 1). In this study, we report the isolation, structure elucidation, and bioactive evaluation of these isolated marine natural products.

## 2. Results and Discussion

The Mariana-Trench-sourced strain SY1965 (Figure S1, Supplementary Materials) was identified as *Streptomyces* sp. SY1965, according to its 16S rDNA sequence (1416 bp, Figure S2), which was over 99.6% match to those of several *Streptomyces* strains (Table S1). An extract prepared from a large-scale culture of the strain SY1965 in Gauze’s liquid medium with sea salt was separated by column chromatography, followed by high performance liquid chromatography (HPLC) purification, to afford compounds **1**–**12**.

Based on the nuclear magnetic resonance (NMR) spectroscopic analyses, optical rotation values, and the comparison to reported data, the known compounds were identified as (*R*)-1-(3-ethylphenyl)-1,2-ethanediol (**5**) [18], orthocetamol (**6**) [19], *N*-salicyloyl-2-aminopropan-1,3-diol (**7**) [20], *N*-[2-hydroxy-1-(hydroxymethyl) ethyl]-2-methoxybenzamide (**8**) [21], salicylamide (**9**) [22], 4-hydroxymethyl benzoate (**10**) [23], 1,9-dicarbomethoxyphenazine (**11**) [24], and spoxazomicin C (**12**) [25]. The ^13^C and ^1^H NMR data of compounds **5**–**12** were reported in Tables S2–S4.

**Figure 2:**
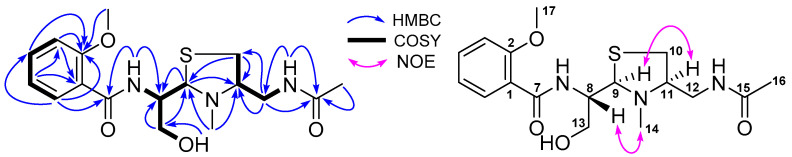
Correlation spectroscopy (COSY), key heteronuclear multiple bond correlation (HMBC), and nuclear Overhauser effect (NOE) correlations of streptothiazomycin A (**1**).

Compound **1** was obtained as a white amorphous powder and had a molecular formula C_17_H_25_N_3_O_4_S, which was deduced from its high-resolution electrospray ionization mass spectroscopy (HRESIMS) ions at *m/z* 368.1642 [M + H]^+^ and 390.1462 [M + Na]^+^, as well as its ^13^C NMR data. Its ^13^C NMR spectrum showed 17 carbon signals for two carbonyls (*δ*_C_ 169.2, 164.0), an aryl (*δ*_C_ 157.4, 132.8, 131.2, 120.7, 121.4, and 112.3), one methoxyl (*δ*_C_ 56.1), one oxymethylene (*δ*_C_ 62.2), three methines (*δ*_C_ 73.9, 69.6, and 52.8), two methylenes (*δ*_C_ 40.7, 33.1), and two methyl (*δ*_C_ 39.6 and 22.4), which were assigned by the analysis of distortionless enhancement by polarization transfer (DEPT) and heteronuclear multiple quantum correlation (HMQC) spectra. The downfield region (*δ*_H_ 7.0–9.0 ppm) of the ^1^H NMR spectrum of **1** showed six signals, of which four were attributed to the aromatic group (*δ*_H_ 7.95, 7.49, 7.18, and 7.05) and two were assigned to two NH groups (*δ*_H_ 8.43 and 7.69) based on the HMQC spectrum. Heteronuclear multiple bond correlation (HMBC) correlations (Figure 2) of NH-7 (*δ*_H_ 8.43, d, 8.3 Hz) with C-7 (*δ*_C_ 164.0) and NH-12 (*δ*_H_ 7.69, t, 5.7 Hz) with C-15 (*δ*_C_ 169.2) indicated the presence of two amide groups in the structure. correlation spectroscopy (COSY) correlations (Figure 2) of H-3 (*δ*_H_ 7.18, d, 7.5 Hz) with H-4 (*δ*_H_ 7.49, d, 7.5, 1.8 Hz), and H-5 (*δ*_H_ 7.05, t, 7.5 Hz) with H-4 and H-6 (*δ*_H_ 7.95, dd, 7.5, 1.8 Hz) as well as HMBC correlations of H_3_-17 (*δ*_H_ 3.93, s) with C-2 (*δ*_C_ 157.4) and H-6 with C-7 demonstrated the presence of a 1,2-disubstituted aryl with a methoxy at C-2 and an amide group at C-1. Similarly, HMBC correlations of both NH-12 and H_3_-16 (*δ*_C_ 1.66, s) with C-15 proved the presence of an acetamido. The aryl and two amide groups accounted for six out of seven degree of unsaturation required by the molecular formula, implying an additional ring in the structure of **1**. In the COSY spectrum, two spin systems of NH-7/H-8/H-9/H-13 and H-10/H-11/H-12/NH-12 were observed, revealing two partial structures of –NH–CH(CH_2_OH)–CH– and –CH_2_–CH–CH_2_–NH–, which were also supported by HMBC correlations. The downfield ^13^C chemical shifts for two methines (*δ*_C_ 73.9 and 69.6) and one methyl (*δ*_C_ 39.6) suggested that these carbons were linked to heteroatoms. HMBC correlations of H_3_-14 (*δ*_H_ 2.36) with C-9 (*δ*_C_ 73.9) and C-11 (*δ*_C_ 69.6), and H-9 (*δ*_H_ 4.22, d, 4.3 Hz) with C-11 and C-14 (*δ*_C_ 39.6), confirmed a partial structure of –. Based on the analyses of these NMR data, in consideration of a sulphur atom in the molecular formula, the additional ring was proposed to be a thiazolidine. HMBC correlations of H-6 with C-7, NH-7 with C-7 and C-8, H-12 (*δ*_H_ 2.93) with C-15, and NH-12 with C-12 and C-15 established the linkage of the 1,2-disubstituted aryl, the thiazolidine-contained part, and the acetamido. To date, the planar structure of **1** has been determined as shown.

**Figure 3:**
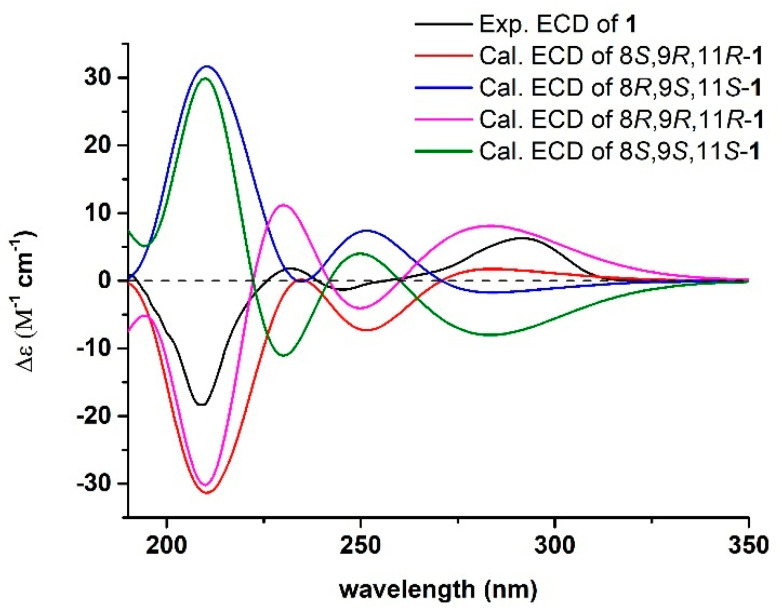
Experimental electronic circular dichroism (ECD) spectrum of streptothiazomycin A (**1**, 190–350 nm) in MeOH and the calculated ECD spectra of 8*S*,9*R*,11*R*-**1**, 8*R*,9*S*,11*S*-**1**, 8*R*,9*R*,11*R*-**1**, and 8*S*,9*S*,11*S*-**1** at the b3lyp/6-311+g(d, p) level in MeOH.

The configuration of **1** was assigned by a combination of nuclear Overhauser effect spectroscopy (NOESY) experiment, electronic circular dichroism (ECD) and ^13^C NMR calculations. NOE correlations (Figure 2) of H-9 with H-11 and H-8 with H_3_-14 suggested a same spatial orientation for H-9 and H-11. First, four model molecules of 8*R*,9*R*,11*R*-**1**, 8*S*,9*R*,11*R*-**1**, 8*R*,9*S*,11*S*-**1**, and 8*S*,9*S*,11*S*-**1** were selected for the time-dependent density-functional theory (TDDFT) ECD calculations in proper solvent (MeOH) [26,27,28] by employing the conductor-like polarizable continuum model (CPCM). As shown in Figure 3, the experimental ECD spectrum of **1** showed good agreement with the calculated ECD curves of 8*S*,9*R*,11*R*-**1** and 8*R*,9*R*,11*R*-**1**. Then, ^13^C NMR calculations [29,30,31] were conducted for the two model molecules of 8*S*,9*R*,11*R*-**1** and 8*R*,9*R*,11*R*-**1**. The results (Table S5) indicated that the experimental ^13^C NMR data of **1** are very close to those of the model molecule of 8*S*,9*R*,11*R*-**1** with a DP4^+^ probability score of 100%. The absolute configuration of **1** was thus assigned to be 8*S*,9*R*,11*R*. Based on the foregoing evidence, the structure of **1** was determined as a novel salicylamide analogue with a unique thiazolidine-contained side chain, named streptothiazomycin A, which was an analogue of spoxazomicins A and B, two rare antitrypanosomal alkaloids isolated from an endophytic actinomycete, *Streptosporangium oxazolinicum* K07-0460T [25]. The ^13^C and ^1^H NMR data of **1** are reported in Table 1.

**Figure 4:**
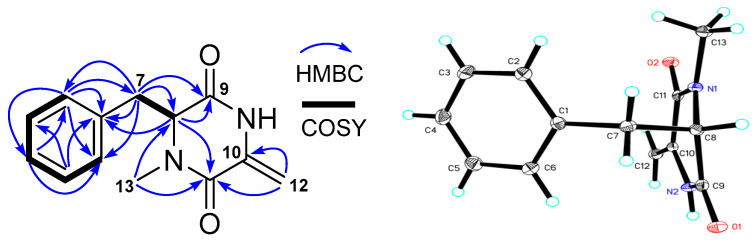
COSY and HMBC correlations and crystal structure of streptodiketopiperazines A (**2**) and B (**3**) (Cu Kα radiation).

A mixture of compounds **2** and **3** was initially obtained as colorless monoclinic crystals, with a molecular formula C_13_H_14_N_2_O_2_ deduced from the HRESIMS ion at *m/z* 231.1128 [M + H]^+^. Analyses of the ^13^C NMR and HMQC spectra indicated the presence of two carbonyls (*δ*_C_ 167.6, 160.9), four pairs of double bonds (*δ*_C_ 135.6, 134.7, 131.2, 131.2, 129.7, 129.7, 128.7, 101.7), one methine (*δ*_C_ 65.4), one methylene (*δ*_C_ 38.3), and one methyl (*δ*_C_ 33.6). Further analyses of COSY and HMBC correlations (Figure 4) δ revealed that the planar structure of **2** and **3** was composed of two units of a monosubstituted aryl and a diketopiperazine skeleton with a = CH_2_ group (*δ*_C_ 101.7; *δ*_H_ 4.96, s, 4.45, s) at C-12 and a methyl at C-13 (*δ*_C_ 33.6; *δ*_H_ 3.12), which were connected to each other through the methylene (*δ*_C_ 38.3; *δ*_H_ 3.25, 3.16). The established structure was further confirmed by the result (Figure 4) from single-crystal X-ray diffraction. However, analysis of the crystal structural data and the optical rotation value (−2°) suggested that **2** and **3** were a racemate. This racemic mixture was further separated on a chiral column (Figure S3) to give **2** and **3** with optical rotation values of −24.8° and +20.7°, respectively. Optical rotation (OR) calculations [32,33] were used to determine the configuration of C-8, the only chiral center for **2** and **3**. The results (Tables S15 and S17) showed a negative OR value (−32.0°) for 8*S* and a positive OR value (+37.1°) for 8*R.* Accordingly, an 8*S* configuration was assigned for **2** and 8*R* for **3**. Therefore, their structures were elucidated as two new diketopiperazine derivatives, named streptodiketopiperazines A (**2**) and B (**3**), which were structural similar to (3*Z*,6*S*)-3-ethylidene-1-methyl-6-(phenylmethyl)-2,5-piperazinedione, an A-factor mimic that restores antibiotic biosynthesis and morphogenesis in *Streptomyces globisporus* 1912-B2 and *Streptomyces griseus* 1439 [34].

Compounds **4** and **5** were also obtained as a racemic mixture, corresponding to a molecular formula, C_10_H_14_O_2_, deduced from the HRESIMS ion at *m*/*z* 165.0914 [M − H]^−^. They were separated on a chiral column (Figure S4) to afford **4** and **5** with optical rotation values of +16.0° and −9.6°, respectively. Based on the ^13^C and ^1^H NMR data and optical values as well as the comparison of the reported data [18], compound **5** was identified as known phenylethanediol (*R*)-1-(3-ethylphenyl)-1,2-ethanediol and **4** was elucidated as (*S*)-1-(3-ethylphenyl)-1,2-ethanediol, a new phenylethanediol.

All isolated compounds **1**–**12** were tested for their antiproliferative activity against human glioma U87MG and U251 cells by the Sulforhodamine B (SRB) assay [35]. Doxorubicin (DOX, an anticancer drug) was used as a positive control. Unfortunately, none of these tested compounds showed antiproliferative activity at a concentration of 50 μM. Obviously, the antiproliferative compounds responsible for the activity of the crude extract were lost during the process of separation.

The antimicrobial activities of compounds **1**–**12** against methicillin-resistant *Staphylococcus aureus* (MRSA)*,* *Escherichia coli*, and *Candida albicans* were also evaluated by the micro-broth dilution method as described in the previous report [36]. Vancomycin (an antibiotic against MRSA), gentamicin (an antibiotic against both Gram-positive and Gram-negative bacteria), and amphotericin B (an antifungal drug) were used as positive controls. The results (Table S6) showed that new compounds streptothiazomycin A (**1**) and streptodiketopiperazines A (**2**) and B (**3**) as well as known compounds [2-hydroxy-1-(hydroxymethyl)ethyl]-2-methoxybenzamide (**8**), salicylamide (**9**), and 4-hydroxymethyl benzoate (**10**) exhibited weak antifungal activity against *C. albicans* with an MIC value of 47, 42, 42, 43, 38, and 38 μg/mL, respectively. Spoxazomicin C (**12**) showed stronger antimicrobial activities, with MIC values of 4 μg/mL for MRSA and 10 μg/mL for *C. albicans*.

## 3. Materials and Methods

### 3.1. General Experimental Procedures

Ultraviolet (UV), infrared radiation (IR), optical rotation (OR), and electronic circular dichroism (ECD) spectra were measured on a METASH UV-8000 (Shanghai METASH Instruments Co. Ltd., Shanghai, China), a Nicolet^TM^ IS^TM^ 10 FT-IR spectrometer (Thermo Fisher Scientific, Waltham, MA, USA), a RUDOLPH AutopolⅠAutomatic polarimeter, and a JASCO J-815 spectropolarimeter (JASCO Co. Tokyo, Japan), respectively. HRESIMS data were obtained on an Agilent 6230 Time of Flight Liquid Chromatography/Mass Spectrometry (TOF LC/MS) spectrometer. NMR data were acquired on a JEOL 600 spectrometer, using a standard JEOL pulse sequences for 1D and 2D (gHMQC, gHMBC, and gCOSY) NMR experiments and chemical shifts were expressed in *δ* (ppm) relative to DMSO-*d*_6_ (*δ*_C_ 39.5, *δ*_H_ 2.50), CH_3_OH-*d*_4_ (*δ*_C_ 49.15, *δ*_H_ 3.31), and CHCl_3_-*d*_1_ (*δ*_C_ 77.0, *δ*_H_ 7.26), respectively. X-ray diffraction analysis was carried out on an Xcalibur Atlas Gemini Ultra diffractometer (Agilent Technologies) with Cu Kα radiation (λ = 1.54184 Å) at 100 K. Melting point was measured with a WRX-4 microscope apparatus (Shanghai Yice Apparatus & Equipment Co. Ltd., Shanghai, China) and was uncorrected. Octadecyl-functionalized silica gel (ODS, Cosmosil 75C_18_-Prep, Nacalai Tesque Inc., Japan), Diaion HP-20 (Mitsubishi Chemical, Tokyo, Japan), and Sephadex LH-20 (Amersham Biosciences, Little Chalfont, UK) were used for column chromatography. HPLC separation was performed on a CXTH LC-3000 preparative HPLC system (Beijing Chuangxin Tongheng Science & Technology Co. Ltd., Beijing, China) using a CT-30 column (Fuji-C_18_, 280 × 30 mm, 10 μm) or an Agilent 1260 HPLC system using Agilent Zorbax SB-C_18_ columns (250 × 9.2 mm, 5 μm) or a CHIRALCEL OJ-RH column (150 × 4.6 mm, 5 μm). All solvents used for this study were purchased from the Sinopharm Chemical Reagent Co. Ltd. (Shanghai, China). Human glioma U87MG (JDS-2568), U251 (XB-0439) cells were ordered from the Cell Bank of the Chinese Academy of Sciences. Methicillin-resistant *Staphylococcus aureus* (MRSA) ATCC 43300, *Escherichia coli* ATCC 25922, and Candida albicans ATCC 10,231 were used for antimicrobial assay. Doxorubicin (DOX, >98.0%) was purchased from Sigma-Aldrich. Gentamicin (99.6%), vancomycin (98.0%) and amphotericin B (>95.0%) were purchased from Meilune Biotechnology Co. Ltd. (Dalian, China). Gauze’s agar medium (Soluble Starch 20 g, KNO_3_ 1 g, MgSO_4_7H_2_O 0.5 g, NaCl 0.5 g, K_2_HPO_4_ 0.5 g, FeSO_4_ 0.01 g, Agar 15 g, water 1 L). Sea salt was bought from the Zhejiang Province Salt Industry Group Company, Ltd. Artificial seawater (sea salt 35 g, water 1 L) was made in the laboratory.

### 3.2. Isolation and Taxonomic Identification of Strain SY1965

Strain SY1965 was isolated from hadal sediments MTD11000, which was collected from the Mariana Trench (11°20′ N and 142°11.5′ E) at depth 11,000 m on November, 2018. Briefly, the soil was dried at 55 °C for 90 min to stimulate the germination of actinomycete spores and the dried soil (1 g) was diluted with sterile water to make dilutions of 10^−1^, 10^−2^, and 10^−3^ g/mL. Each dilution (200 µL) was covered on the surface of different solid media and then incubated at 28 °C for 14 days. The single pure colony of SY1965 was picked from the 10^−1^ g/mL suspension in Gauze’s agar medium and then transferred to another fresh Gauze’s agar medium plate. After another 3 days of growth at 28 °C, the single colony (SY1965) that grew well was transferred onto Gauze’s agar slants and stored at 4 °C for further study.

The 16S rDNA analysis of strain SY1965 was performed by Legenomics (Hangzhou, China) and its DNA sequence was compared to those in the GenBank database using nucleotide BLAST (Basic Local Alignment Search Tool). The 16S rDNA sequence of strain SY1965 has been deposited in GenBank (accession number: MT421944). The strain *Streptomyces* sp. SY1965 was preserved at the Laboratory of Institute of Marine Biology and Pharmacology, Ocean College, Zhoushan campus, Zhejiang University, Zhoushan, China.

### 3.3. Mass Culture of Strain SY1965

The pure colonies of SY1965 were inoculated in an Erlenmeyer flask (500 mL) containing 250 mL Gauze′s liquid medium and then incubated at 28 °C for three days on a rotary shaker at 180 rpm to produce seed broth. The seed broth (10 mL) was transferred into a 500 mL Erlenmeyer flask, which contained 250 mL Gauze’s liquid medium with sea salt. All flasks were placed on rotary shakers at 180 rpm for incubation at 28 °C for 14 days. A total of 50 L culture was prepared for this study.

### 3.4. Extraction and Isolation of Compounds ***1**–**12***

The 50 L culture of strain SY1965 was centrifuged to yield filtrate and mycelia. The mycelia were extracted with MeOH three times to give mycelium extract. The filtrate was absorbed onto a HP-20 column eluting with water and then MeOH to obtain crude filtrate extract. The mycelium and filtrate extracts were combined and then partitioned with EtOAc three times to afford a crude extract (2 g). This crude extract (2 g) was subjected to a column of ODS eluting successively with 30% MeOH, 50% MeOH, 70% MeOH, and 100% MeOH to give three fractions (Frs. A–C) based on the results of TLC and HPLC analyses. Fr. A was fractionated by Sephadex LH-20 using 40% MeOH to give subfractions A_1_–A_3_. Fr. A_1_ was separated by using CXTH LC-3000 preparative HPLC (column: Fuji-C_18_ CT-30, 280 × 30 mm, 10 µm; flow rate: 10 mL/min; UV detection: 210 nm) with linear gradient from 30% to 60% MeOH in 40 min, isocratic elution with 60% MeOH from 40.01 to 50 min to afford a mixture of **6** and **7** (5 mg, t_R_ 30 min), **8** (3.3 mg, t_R_ 32 min), a mixture of **2** and **3** (2.2 mg, t_R_ 39 min), **1** (1.8 mg, t_R_ 45 min). The mixture of **6** and **7** was separated by Agilent 1260 HPLC purification (column: Agilent Zorbax SB-C_18_, 250 × 9.2 mm, 5 µm; mobile phase: MeCN/H_2_O, 21/79; flow rate: 1 mL/min; UV detection: 210 nm) to give **7** (3.4 mg, t_R_ 17 min) and **6** (3.0 mg, t_R_ 23 min). The mixture of **2** and **3** was separated on a chiral HPLC column (CHIRALCEL^®^ OJ-RH, 150 × 4.6 mm, 5 μm; mobile phase: MeCN/H_2_O, 11/89; flow rate: 0.4 mL/min; UV detection: 210 nm) to give **2** (0.7 mg, t_R_ 24 min) and **3** (0.6 mg, t_R_ 26 min). Compounds **9** (4.0 mg, t_R_ 28 min), **10** (3.3 mg, t_R_ 34 min) and **12** (4.0 mg, t_R_ 40 min) were obtained from Fr. A_3_ by preparative HPLC using the same CT-30 column with linear gradient from 30% to 100% MeOH in 46 min. Purification of Fr. A_2_ and B by the same SB-C_18_ column and flow rate with UV detection of 210 nm gave a mixture of **4** and **5** (2 mg, t_R_ 13.5 min, MeCN/H_2_O, 50/50) from Fr. A_2_, **11** (1.0 mg, t_R_ 23.5 min, MeCN/H_2_O, 49/51) from Fr. B, respectively. The mixture of **4** and **5** was further separated by the same chiral HPLC column (mobile phase: MeCN/H_2_O, 15/85; flow rate: 0.5 mL/min; UV detection: 210 nm) to give **5** (0.7 mg, t_R_ 17 min) and **4** (0.7 mg, t_R_ 18.5 min).

Streptothiazomycin A (**1**): white amorphous powder; molecular formula C_17_H_25_N_3_O_4_S; [α]_D_^20^ +18.3° (*c* 0.15, MeOH); ECD (10 mg/L, MeOH) λ_max_ (Δε) 209 (−18.32), 232 (+1.80), 245 (−1.32), 291 (+6.27) nm; UV (MeOH) λ_max_ (log ε) 206 (4.48), 233 (3.95), 289 (3.48) nm; IR (MeOH) *ν*_max_ 3342, 2928, 2853, 2797, 1636, 1600, 1530, 1483, 1369, 1295, 1238, 1163, 1108, 1020, 759 cm^−1^; ^13^C NMR data (150 MHz, in DMSO-*d*_6_), Table 1, ^1^H NMR data (600 MHz, in DMSO-*d*_6_), Table 1; HRESIMS *m*/*z* 368.1642 [M + H]^+^ (calcd for C_17_H_26_N_3_O_3_S, 368.1644) and 390.1462 [M + Na]^+^ (calcd for C_17_H_25_N_3_NaO_4_S, 390.1463).

Streptodiketopiperazines A (**2**) and B (**3**): colorless monoclinic crystals; molecular formula C_13_H_14_N_2_O_2_; m.p. 210–212 °C; [α]_D_^20^ −24.8° (*c* 0.1, MeOH) for **2**, [α]_D_^20^ +20.7° (*c* 0.1, MeOH) for **3**; UV (MeOH) λ_max_ (log ε) 204 (4.04) nm; IR (MeOH) ν_max_ 3204, 3030, 2928, 1687, 1626, 1455, 1395, 1338, 702 cm^–1^; ^13^C NMR data (150 MHz, in CH_3_OH-*d*_4_), Table 1, ^1^H NMR data (600 MHz, in CH_3_OH-*d*_4_), Table 1; HRESIMS *m*/*z* 231.1128 [M + H]^+^ (calcd for C_13_H_15_N_2_O_2_, 231.1134).

Crystal Data for streptodiketopiperazines A (**2**) and B (**3**): C_13_H_14_N_2_O_2_ (*M* = 230.26 g/mol): monoclinic, space group P2_1_/c (no. 14), *a* = 12.0238(12) Å, *b* = 7.8159(7) Å, *c* = 12.0101(11) Å, *β* = 97.506(9)°, *V* = 1119.00(18) Å^3^, *Z* = 4, *T* = 100.00(10) K, μ(Mo Kα) = 0.094 mm^−1^, *Dcalc* = 1.367 g/cm^3^, 4429 reflections measured (6.234° ≤ 2Θ ≤ 49.998°), 1967 unique (*R*_int_ = 0.0336, R_sigma_ = 0.0530) which were used in all calculations. The final *R*_1_ was 0.0458 (I > 2σ(I)) and *wR*_2_ was 0.1030 (all data). The crystal data and structure refinement parameters of **2** and **3** were also reported in Table S19. Crystallographic data has been deposited at the Cambridge Crystallographic Data Centre (CCDC Number: 2004226). Copies of the data can be obtained free of charge from Cambridge Crystallographic Data Centre, 12, Union Road, Cambridge CB2 1EZ, UK (fax (+44)1223-336-033; or e-mail: data_request@ccdc.cam.ac.uk).

(*S*)-1-(3-ethylphenyl)-1,2-ethanediol (**4**): white amorphous powder; molecular formula C_10_H_14_O_2_; [α]_D_^20^ +16.0° (*c* 0.10, MeOH); UV (MeOH) λ_max_ (log ε) 201 (3.72), 210 (3.64) nm; IR (MeOH) ν_max_ 3342, 2963, 2926, 2857, 1457, 1259, 1081, 1032 cm^−1^; ^13^C NMR data (150 MHz, in MeOH-*d*_4_), see Table 1, ^1^H NMR data (600 MHz, in MeOH-*d*_4_), see Table 1. HRESIMS *m/z* 165.0914 [M − H]^−^ (calcd for C_10_H_13_O_2_, 165.0916).

### 3.5. ECD Calculation

Monte Carlo conformational searches were carried out by means of the Spartan’s 10 software using Merck Molecular Force Field (MMFF). Five low-energy conformers of each model molecule of 8*R*,9*R*,11*R*-**1**, 8*S*,9*R*,11*R*-**1**, 8*R*,9*S*,11*S*-**1**, and 8*S*,9*S*,11*S*-**1** (Tables S7, S9, S11 and S13) were selected for ECD calculations. The selected conformers were then optimized at the B3LYP/6-31+g (d, p) level in MeOH using the conductor-like polarizable continuum model (CPCM). The theoretical calculation of ECD was conducted in MeOH using Time-Dependent Density Functional Theory (TD-DFT) at the B3LYP/6-311+g (d, p) level for all selected conformers. Rotatory strengths for a total of 30 excited states were calculated. ECD spectra were generated using the program SpecDis 1.6 (University of Würzburg, Würzburg, Germany) and GraphPad Prism 5 (University of California, San Diego, CA, USA) from dipole-length rotational strengths by applying Gaussian band shapes with sigma = 0.3 eV.

### 3.6. ^13^C NMR Calculations

Monte Carlo conformational searches were carried out by means of the Spartan’s 10 software using Merck Molecular Force Field (MMFF). The conformers with Boltzmann population of over 5% were chosen for NMR calculations, and then the conformers were initially optimized at B3LYP/6-31g (d, p) level in gas. Meanwhile, gauge-independent atomic orbital (GIAO) calculations of ^13^C NMR chemical shifts were accomplished by density functional theory (DFT) at the mPWLPW91-SCRF (DMSO)/6-311+g (d, p) level with the PCM solvent continuum model in Gaussian 09 software. The calculated NMR data of the lowest energy conformers for 8*R*,9*R*,11*R*-**1** and 8*S*,9*R*,11*R*-**1** were averaged according to the Boltzmann distribution theory and their relative Gibbs free energy. The ^13^C NMR chemical shifts for TMS were calculated by the same protocol and used as reference [37]. The experimental and calculated data were analyzed by the improved probability DP4+ method for isomeric compounds. A significantly higher DP4^+^ probability score of 8*R*,9*R*,11*R*-**1** and 8*S*,9*R*,11*R*-**1** suggested the correctness of its configuration.

### 3.7. Culture of Human Glioma Cells

Human glioma U87MG and U251 cells were cultured in Minimum Essential Medium (MEM), Gibco and Dulbecco’s Modified Eagle Medium (DMEM), Gibco with 10% FBS, respectively. All cells were incubated at 37 °C in a humidified incubator with 5% CO_2_ incubator. Cells after the third generation were used for experiment.

### 3.8. Sulforhodamine B (SRB) Assay

The SRB assay, as described in a previous report [35], was applied to determine the antiproliferative activity of all isolated compounds **1**–**12** against human glioma U87MG and C251 cells. Doxorubicin (DOX) was used as a positive control.

### 3.9. Antimicrobial Assay

The antimicrobial activities of compounds **1**–**12** inhibiting the growth of MRSA, *E. coli* and *C. albicans* were also evaluated by the micro-broth dilution method, as described in previous publication [36]. Vancomycin, gentamicin, and amphotericin B were used as positive controls and DMSO was used as a negative control.

## 4. Conclusions

New streptothiazolidine A (**1**), streptodiketopiperazines A (**2**) and B (**3**), and (*S*)-1-(3-ethylphenyl)-1,2-ethanediol (**4**) were discovered and characterized from the actinomycete *Streptomyces* sp. SY1965 isolated from a Mariana Trench sediment collected at depth of 11,000 m. The racemic mixtures of (±)-streptodiketopiperazine and (±)-1-(3-ethylphenyl)-1,2-ethanediol were separated by using a chiral HPLC column. Streptothiazolidine A is a rare salicylamide analogue with a unique thiazolidine-contained side chain. Streptothiazomycin A and streptodiketopiperazines A and B exhibited antifungal activity.

## Figures and Tables

**Table 1:** ^13^C (150 MHz) and ^1^H (600 MHz) NMR data of compounds **1**–**4**.

No.	1 *^a^*		2 + 3 *^b^*		4 *^b^*	
	C, Type	H (*J* in Hz)	C, Type	H (*J* in Hz)	C, Type	H (*J* in Hz)
1	121.4, C	–	135.6, C	–	145.6, C	–
2	157.4, C	–	131.2, CH	7.04, dd (8.2, 2.6)	127.1, CH	7.22, s
3	112.3, CH	7.18, d (7.5)	129.7, CH	7.20–7.24, m	143.4, C	–
4	132.8, CH	7.49, dd (7.5, 1.8)	128.7, CH	7.20–7.24, m	128.2, CH	7.10, d (7.8)
5	120.7, CH	7.05, t (7.5)	129.7, CH	7.20–7.24, m	129.4, CH	7.24, t (7.8)
6	131.2, CH	7.95, dd (7.5, 1.8)	131.2, CH	7.04, dd (8.2, 2.6)	124.9, CH	7.16, d (7.8)
7	164.0, C	–	38.3, CH_2_	3.25, dd (14.6, 5.0); 3.16, dd (14.6, 3.3)	30.0, CH_2_	2.62, q (7.5)
8	52.8, CH	4.25, m	65.4, CH	4.42, dd (5.0, 3.3)	16.4, CH_3_	1.21, t (7.5)
9	73.9, CH	4.22, d (4.3)	167.6, C	–	76.3, CH	4.64, t (7.5)
10	33.1, CH_2_	2.93, m; 2.66, dd (10.3, 8.9)	134.7, C	–	69.0, CH_2_	3.59, m
11	69.6, CH	2.80, m	160.9, CH	–		
12	40.7, CH_2_	3.35, m; 2.93, m	101.7, CH_2_	4.96, s; 4.45, s		
13	62.2, CH_2_	3.59, m; 3.28, m	33.6, CH_3_	3.12, s		
14	39.6, CH_3_	2.36, s				
15	169.2, C	–				
16	22.4, CH_3_	1.66, s				
17	56.1, CH_3_	3.93, s				
NH-7	–	8.43, d (8.3)				
NH-12	–	7.69, t (5.7)				

*^a^* The data were recorded in DMSO-*d*_6_; *^b^* The data were recorded in MeOH-*d*_4_.

## Supplementary Materials

The following are available online at https://www.mdpi.com/1660-3397/18/8/385/s1, Figure S1: Colonies of *Streptomyces* sp. SY1965; Figure S2: 16S rDNA sequence of *Streptomyces* sp. SY1965; Figures S3–S4: HPLC separation chromatograms of compounds **2**–**5** on the chiral HPLC column; Figures S5–S38: NMR and HRESIMS spectra of compounds **1**–**4**; Table S1: Score statistics for sequence alignment of strain SY1965; Tables S2–S4: ^13^C and ^1^H NMR data of compounds **5**–**12**; Table S5: Experimental ^13^C NMR data of **1** and calculated ^13^C NMR data of 8*R*,9*R*,11*R*-**1** (**1a**) and 8*S*,9*R*,11*R*-**1**(**1b**) (ppm); Table S6: Antimicrobial activities of compounds **1**–**12**; Tables S7–S18: Data of ECD and ^13^C NMR calculations; Table S19: Crystal data and structure refinement parameters of streptodiketopiperazines A (**2**) and B (**3**).
