# Supplementary material for: New Antifungal Metabolites from the Mariana Trench Sediment-Associated Actinomycete Streptomyces sp. SY1965
Source: Mar Drugs. 2020 Jul 24;18(8):385. doi: 10.3390/md18080385 (PMC7459909; doi:10.3390/md18080385)
Supplement: Supplementary file 1 [file marinedrugs-18-00385-s001.pdf]

SUPPLEMENTARY DATA FOR

New Antifungal Metabolites from the Mariana Trench Sediment-Associated Actinomycete *Streptomyces* sp. SY1965

Wenwen Yi[a], Le Qin[a], Xiao-Yuan Lian[b,]*, Zhizhen Zhang[a,] **

[1] Ocean College, Zhoushan Campus, Zhejiang University, Zhoushan 316021, China; 18790658213@163.com (W.Y.), qinle19951102@126.com (L.Q.)

[2] College of Pharmaceutical Sciences, Zhejiang University, Hangzhou 310058, China;

* Correspondence: xylian@zju.edu.cn (X.L.); zzhang88@zju.edu.cn (Z.Z.); Tel.: +86-13575476388 (X.L.); +86-13675859706 (Z.Z.)

Content

Table S1. Score statistics for sequence alignment of strain SY1965……………………..…4
Table S2. ^{13}C NMR data of compounds **5–10** (150 MHz)………………………………..4
Table S3. ^{1}H NMR data for compounds **5–10** (600 MHz, *J* in Hz)……………………......5
Table S4. ^{13}C (150 MHz) and ^{1}H (600 MHz) NMR data of compounds **11–12**……………..5
Table S5. Experimental ^{13}C NMR data of **1** and calculated ^{13}C NMR data of 8*R*,9*R*,11*R*-**1** (**1a**) and 8*S*,9*R*,11*R*-**1**(**1b**) (ppm)……………………………………………………………6
Physicochemical data of known compounds **5–12**………………………………………….6
Table S6. Antimicrobial activities of compounds **1–12** (MIC: μg/mL)…………………….7
Table S7. Gibbs free energies and equilibrium populations of low-energy conformers of 8*R*,9*R*,11*R*-**1**……………………………………………………………………………..8
Table S8. Cartesian coordinates for the low-energy reoptimized MMFF conformers of 8*R*,9*R*,11*R*-**1** at B3LYP/6-31G(d, p) level of theory in gas…………………………………8
Table S9. Gibbs free energies and equilibrium populations of low-energy conformers of 8*S*,9*R*,11*R*-**1**……………………………………………………………………………14
Table S10. Cartesian coordinates for the low-energy reoptimized MMFF conformers of 8*S*,9*R*,11*R*-**1** at B3LYP/6-31G(d, p) level of theory in gas………………………………...14
Table S11. Gibbs free energies and equilibrium populations of low-energy conformers of 8*R*,9*S*,11*S*-**1**……………………………………………………………………………20
Table S12. Cartesian coordinates for the low-energy reoptimized MMFF conformers of 8*R*,9*S*,11*S*-**1** at B3LYP/6-31G(d, p) level of theory in gas………………………………...20
Table S13. Gibbs free energies and equilibrium populations of low-energy conformers of 8*S*,9*S*,11*S*-**1**……………………………………………………………………………..27
Table S14. Cartesian coordinates for the low-energy reoptimized MMFF conformers of 8*S*,9*S*,11*S*-**1** at B3LYP/6-31G(d, p) level of theory in gas…………………………………27
Table S15. Gibbs free energies and equilibrium populations of low-energy conformers of *R*-OR-**2**……………………………………………………………………………………33
Table S16. Cartesian coordinates for the low-energy reoptimized MMFF conformers of *R*-OR-**2** at B3LYP/6-311+G(d, p) level of theory in CH_3OH……………………………..33
Table S17. Gibbs free energies and equilibrium populations of low-energy conformers of *S*-OR-**2**……………………………………………………………………………………38
Table S18. Cartesian coordinates for the low-energy reoptimized MMFF conformers of *S*-OR-**2** at B3LYP/6-311+G(d, p) level of theory in CH_3OH……………………………..38
Figure S1. Colonies of *Streptomyces* sp. SY1965…………………………………………42
Figure S2. 16S rDNA sequence of *Streptomyces* sp. SY1965…………………………..……42

Figure S3. HPLC separation chromatogram of streptodiketopiperazines A (**2**) and (**3**) on the chiral HPLC column……………………..…………………………………………..43
Figure S4. HPLC separation chromatogram of (*S*)-1-(3-ethylphenyl)-1,2-ethanediol (**4**) and (*R*-)1-(3-ethylphenyl)-1,2-ethanediol (**5**) on the chiral HPLC column……………………43
Figures S5-7. ^{1}H NMR spectra of streptothiazomycin A (**1**)……………..……………..44
Figures S8-10. ^{13}C NMR spectra of streptothiazomycin A (**1**).……………….…………..45
Figure S11. DEPT NMR spectrum of streptothiazomycin A (**1**) ……………….……………47
Figures S12-13. HMQC spectra of streptothiazomycin A (**1**) ……….………..………….47
Figures S14-15. COSY spectra of streptothiazomycin A (**1**)…….…………………………48
Figures S16-18. HMBC spectra of streptothiazomycin A (**1**) ……..…………………….49
Figures S19-20. NOESY spectra of streptothiazomycin A (**1**) ……………………………51
Figure S21. HRESIMS spectrum of streptothiazomycin A (**1**) ……………………………52
Figure S22. ^{1}H NMR spectrum of streptodiketopiperazines A (**2**) and B (**3**)………………52
Figures S23-25. ^{13}C NMR spectra of streptodiketopiperazines A (**2**) and B (**3**)……..……53
Figure S26. HMQC spectrum of streptodiketopiperazines A (**2**) and B (**3**)………………..54
Figure S27. COSY spectrum of streptodiketopiperazines A (**2**) and B (**3**)…………..……..55
Figures S28-30. HMBC spectra of streptodiketopiperazines A (**2**) and B (**3**)……….………55
Figure S31. HRESIMS spectrum of streptodiketopiperazines A (**2**) and B (**3**) ….…………57
Figures S32-34. ^{1}H NMR spectra of (*S*)-1-(3-ethylphenyl)-1,2-ethanediol (**4**) …..……..…57
Figure S35-37. ^{13}C NMR spectra of (*S*)-1-(3-ethylphenyl)-1,2-ethanediol (**4**) ….…………59
Figure S38. HRESIMS spectrum of (*S*)-1-(3-ethylphenyl)-1,2-ethanediol (**4**)……………60
Table S19. Crystal data and structure refinement parameters of streptodiketopiperazines A (**2**) and B (**3**)………………………………………………………………..……….61

Table S1. Score statistics for sequence alignment of strain SY1965

Accession	Description	Max score	Total score	Query coverage	E value	Ident
MN058252.1	*Streptomyces* sp. strain DCPA3-2 16S ribosomal RNA gene	2588	2588	99%	0.0	99.65%
MH633842.1	*Streptomyces* sp. strain BF9 16S ribosomal RNA gene	2588	2588	99%	0.0	99.65%
MK463974.1	*Streptomyces fimicarius* strain A1 contig-1 16S ribosomal RNA gene	2588	2588	99%	0.0	99.65%
MG197994.1	*Streptomyces fimicarius* strain BWL-H1 16S ribosomal RNA gene	2588	2588	99%	0.0	99.65%
MH635267.1	*Streptomyces* griseus strain INA 01181 16S ribosomal RNA gene	2588	2588	99%	0.0	99.65%
MH635263.1	*Streptomyces fimicarius* strain INA 01179 16S ribosomal RNA gene	2588	2588	99%	0.0	99.72%

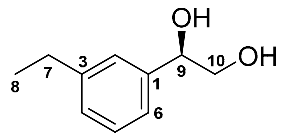

5

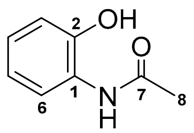

6

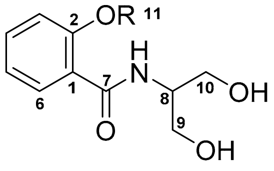

7 R = H **8** R = CH3

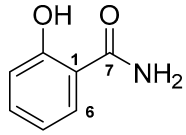

9

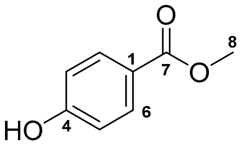

10

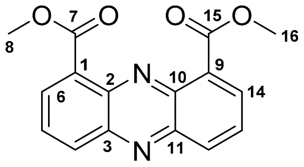

11

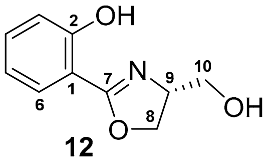

12

Table S2. ^{13}C NMR data of compounds **5**–**10** (150 MHz)

No.	**5**[a]	**6**[b]	**7**[b]	**8**[a]	**9**[a]	**10**[b]
1	145.6, C	126.5, C	116.4, C	122.7, C	116.4, C	119.8, C
2	127.1, CH	148.1, C	159.9, C	159.5, C	162.5, C	131.4, CH
3	143.4, C	115.8, CH	117.4, CH	113.2, CH	118.9, CH	115.4, CH
4	128.2, CH	124.5, CH	133.2, CH	134.5, CH	135.3, CH	162.5, C
5	129.4, CH	118.7, CH	117.9, CH	122.2, CH	119.8, CH	115.4, CH
6	124.9, CH	122.2, CH	128.7, CH	132.5, CH	129.6, CH	131.4, CH
7	30.0, CH_2	168.9, C	167.9, C	168.0, C	174.1, C	166.1, C
8	16.4, CH_3	23.6, CH_3	53.3, CH	54.5, CH	–	51.6, CH_3
9	76.3, CH	–	60.0, CH_2	61.8, CH_2	–	–
10	69.0, CH_2	–	60.1, CH_2	61.8, CH_2	–	–
11	–	–	–	56.8, CH_3	–	–

[a] The data were recorded in MeOH-*d*4; [b] The data were recorded in DMSO-*d*6.

Table S3. ^{1}H NMR data for compounds **5–10** (600 MHz, *J* in Hz)

No.	**5**[a]	**6**[b]	**7**[b]	**8**[a]	**9**[a]	**10**[b]
2	7.22, s	–	–	–	–	7.79, d (8.3)
3	–	6.83, dd (8.2, 1.8)	6.87, d (8.0)	7.15, d (7.8)	6.84, m	6.81, d (8.3)
4	7.10, d (7.6)	6.90, td (7.8, 1.8)	7.33, t (8.0)	7.50, t (7.8)	7.36, t (7.2)	–
5	7.24, t (7.6)	6.72, td (7.8, 1.8)	6.82, t (8.0)	7.05, t (7.8)	6.86, m	6.81, d (8.3)
6	7.16, d (7.6)	7.67, dd (8.2, 1.8)	7.90, d (8.0)	7.99, d (7.8,)	7.75, d (7.2)	7.79, d (8.3)
7	2.62, q (7.5)	–	–	–	–	–
8	1.21, t (7.5)	2.08, s	3.96, m	4.12, m	–	3.78, s
9	4.65, t (7.5)	–	3.50, m	3.68, dd (10.8, 5.6)	–	–
10	3.59, m	–	3.50, m	3.77, dd (10.8, 5.6)	–	–
1-NH		9.30, br s	–	–	–	–
7- NH		–	8.72, br s	–	–	–

[a] The data were recorded in MeOH-d_4; [b] the data were recorded in DMSO-d_6.

Table S4. ^{13}C (150 MHz) and ^{1}H (600 MHz) NMR data of compounds **11–12**

No.	**11**[a]		**12**[b]	
	δ_C, type	δ_H (*J* in Hz)	δ_C, type	δ_H (*J* in Hz)
1	131.4, C	–	112.3, C	–
2	141.1, C	–	161.6, C	–
3	143.2, C	–	117.8, CH	6.91, d (7.8)
4	134.5, CH	8.30, d (7.2)	134.5, CH	7.34, t (7.8)
5	129.8, CH	7.89, dd (7.2, 8.7)	119.6, CH	6.83, t (7.8)
6	133.2, CH	8.47, d (8.7)	129.4, CH	7.63, d (7.8)
7	167.1, C	–	167.7, C	–
8	53.0, CH_3	4.12, s	70.2, CH_2	4.49, m; 4.33, m
9	131.4, C	–	68.7, CH	4.46, m
10	141.1, C	–	64.8, CH_2	3.70, d (4.2)
11	143.2, C	–	–	–
12	134.5, CH	8.30, d (7.2)	–	–
13	129.8, CH	7.89, dd (7.2, 8.7)	–	–
14	133.2, CH	8.47, d (8.7)	–	–
15	167.1, C	–	–	–
16	53.0, CH_3	4.12, s	–	–

[a] The data were recorded in $CHCl_3$-*d*; [b] The data were recorded in MeOH-d_4.

Table S5. Experimental ^{13}C NMR data of **1** and calculated ^{13}C NMR data of 8*R*,9*R*,11*R*-**1** (**1a**) and 8*S*,9*R*,11*R*-**1**(**1b**) (ppm)

No.	**1**	**1a**	**1b**
1	121.4	121.927	124.512
2	157.4	158.946	158.483
3	112.3	109.412	111.257
4	132.8	134.218	134.161
5	120.7	122.150	122.870
6	131.2	136.352	136.759
7	164.0	166.360	168.292
8	52.8	64.845	57.278
9	73.9	78.335	78.065
10	33.1	40.440	38.458
11	69.6	70.845	68.027
12	40.7	36.379	37.323
13	62.2	67.306	63.931
14	39.6	37.191	35.931
15	169.2	168.743	168.357
16	22.4	22.146	21.910
17	56.1	52.553	53.960
DP4^{+}		0.00%	100.00%

Physicochemical data of known compounds 5–12

(*R*)-1-(3-ethylphenyl)-1,2-ethanediol (**5**): white amorphous powder; molecular formula $C_{10}H_{14}O_2$; $[\alpha]_D^{20}$ –9.6° (*c* 0.10, MeOH), *Ref.* [18] $[\alpha]_D^{19}$ –15.3° (*c* 0.09, $CHCl_3$); UV (MeOH) λ_{max} (log ε) 201 (3.72), 210 (3.64) nm; ^{13}C NMR data (150 MHz, in MeOH-*d*4), see Table S2, ^{1}H NMR data (600 MHz, in MeOH-*d*4), see Table S3.

Orthocetamol (**6**): white amorphous powder; molecular formula $C_8H_9NO_2$; ^{13}C NMR data (150 MHz, in DMSO-*d*6), see Table S2, ^{1}H NMR data (600 MHz, in DMSO-*d*6), see Table S3.

N-Salicyloyl-2-aminopropan-1,3-diol (**7**): white amorphous powder; molecular formula $C_{10}H_{13}NO_4$; ^{13}C NMR data (150 MHz, in DMSO-*d*6), see Table S2, ^{1}H NMR data (600 MHz, in DMSO-*d*6), see Table S3.

[2-Hydroxy-1-(hydroxymethyl)ethyl]-2-methoxybenzamide (**8**): white amorphous powder; molecular formula $C_{11}H_{15}NO_4$; UV (MeOH) λ_{max} (log ε) 205 (4.31), 233 (3.83), 289 (3.37) nm; ^{13}C NMR data (150 MHz, in MeOH-d_4), see Table S2, ^{1}H NMR data (600 MHz, in MeOH-d_4), see Table S3.

Salicylamide (**9**): white amorphous powder; molecular formula $C_7H_7NO_2$; ^{13}C NMR data (150 MHz, in MeOH-d_4), see Table S2, ^{1}H NMR data (600 MHz, in MeOH-d_4), see Table S3. HRESIMS *m/z* 138.0543 $[M+H]^+$ (calcd for $C_7H_8NO_2$, 138.0555).

4-Hydroxymethyl benzoate (**10**): white amorphous powder; molecular formula $C_8H_8O_3$; ^{13}C NMR data (150 MHz, in DMSO-d_6), see Table S2, ^{1}H NMR data (600 MHz, in DMSO-d_6), see Table S3.

1,9-Dicarbomethoxyphenazine (**11**): yellow amorphous powder; molecular formula $C_{16}H_{12}N_2O_4$; ^{13}C NMR data (150 MHz, in CHCl$_3$-d), see Table S4, ^{1}H NMR data (600 MHz, in CHCl$_3$-d), see Table S4.

Spoxazomicin C (**12**): white amorphous powder; molecular formula $C_{10}H_{11}NO_3$; $[\alpha]_D^{20}$ +5.3° (*c* 0.10, MeOH), *Ref.* [25] $[\alpha]_D^{20}$ +7.4° (*c* 0.10, MeOH); ^{13}C NMR data (150 MHz, in MeOH-d_4), see Table S4, ^{1}H NMR data (600 MHz, in MeOH-d_4), see Table S4.

Table S6. Antimicrobial activities of compounds **1–12** (MIC: μg/mL)

Compounds	*E. coli*	MRSA	*C. albicans*
**1**	NA	NA	47
**2**	NA	NA	42
**3**	NA	NA	42
**4**	NA	NA	NA
**5**	NA	NA	NA
**6**	NA	NA	NA
**7**	NA	NA	NA
**8**	NA	NA	43
**9**	NA	NA	38
**10**	NA	NA	38
**11**	NA	NA	NA
**12**	NA	4	10
Gentamicin	0.5	NT	NT
Vancomycin	NT	0.3	NT
Amphotericin B	NT	NT	2

NA: No activity at concentration of 50 μg/mL; NT: No testing.

Table S7. Gibbs free energies[a] and equilibrium populations[b] of low-energy conformers of 8*R*,9*R*,11*R*-**1**.

Conformers	In MeOH	
	G	P (%)
8*R*,9*R*,11*R*-**1a**	-957513.94850826	48.64
8*R*,9*R*,11*R*-**1b**	-957513.94913577	48.69
8*R*,9*R*,11*R*-**1c**	-957511.63362387	0.97
8*R*,9*R*,11*R*-**1d**	-957511.58216805	0.89
8*R*,9*R*,11*R*-**1e**	-957511.52506464	0.81

[a] B3LYP/6-31G(d, p), in kcal/mol; [b] from G values at 298.15K.

Table S8. Cartesian coordinates for the low-energy reoptimized MMFF conformers of 8*R*,9*R*,11*R*-**1** at B3LYP/6-31G(d, p) level of theory in gas.

8*R*,9*R*,11*R*-**1a**			Standard Orientation (Ångstroms)		
Center number	Atomic number	Atomic type	X	Y	Z
1.	6.	0.	0.692634	1.762617	-6.245024
2.	6.	0.	1.634801	2.424729	-5.461135
3.	6.	0.	1.628692	2.228815	-4.084040
4.	6.	0.	0.699320	1.388814	-3.456445
5.	6.	0.	-0.250378	0.723884	-4.269677
6.	6.	0.	-0.245060	0.914945	-5.656041
7.	6.	0.	0.832185	1.283813	-1.958647
8.	7.	0.	-0.082976	0.558348	-1.271683
9.	8.	0.	-1.155353	-0.101266	-3.651505
10.	6.	0.	0.003987	0.440770	0.180738
11.	6.	0.	-1.304813	-0.150904	0.741702
12.	16.	0.	-1.555044	-1.961884	0.419717
13.	6.	0.	1.298863	-0.275232	0.650835
14.	8.	0.	2.257540	0.640333	1.148523
15.	6.	0.	-2.188265	1.186186	2.576613
16.	7.	0.	-1.354967	0.051684	2.192220
17.	6.	0.	-1.601876	-1.162333	2.989764
18.	6.	0.	-1.165105	-2.375684	2.163315
19.	6.	0.	-0.844478	-1.074789	4.332856
20.	6.	0.	-2.087137	-0.835330	-4.439957
21.	8.	0.	1.758739	1.869557	-1.376052
22.	7.	0.	0.558309	-0.766194	4.149606
23.	6.	0.	1.564092	-1.567053	4.627722
24.	6.	0.	2.969308	-1.075523	4.318110
25.	8.	0.	1.344989	-2.584311	5.276660
26.	1.	0.	-2.145245	0.355800	0.245665
27.	1.	0.	-2.678996	-1.261657	3.211521
28.	1.	0.	0.066468	1.457391	0.590426
29.	1.	0.	0.679652	1.898487	-7.322504

30.	1.	0.	2.367097	3.083534	-5.916053
31.	1.	0.	2.350084	2.721130	-3.441925
32.	1.	0.	-0.968347	0.407211	-6.281442
33.	1.	0.	-0.764453	0.027117	-1.797093
34.	1.	0.	1.076514	-0.953350	1.474294
35.	1.	0.	1.693828	-0.877892	-0.178636
36.	1.	0.	2.403334	1.269843	0.418436
37.	1.	0.	-2.080269	1.384477	3.646552
38.	1.	0.	-1.863251	2.085893	2.046755
39.	1.	0.	-3.259942	1.023086	2.364146
40.	1.	0.	-0.101213	-2.586586	2.284443
41.	1.	0.	-1.730297	-3.266094	2.444883
42.	1.	0.	-1.323372	-0.318267	4.969160
43.	1.	0.	-0.896777	-2.026942	4.866717
44.	1.	0.	-2.759858	-0.166179	-4.988346
45.	1.	0.	-1.574505	-1.499138	-5.144905
46.	1.	0.	-2.665684	-1.433058	-3.735149
47.	1.	0.	0.785690	0.015866	3.550577
48.	1.	0.	3.621801	-1.942887	4.205083
49.	1.	0.	3.337955	-0.491589	5.168727
50.	1.	0.	3.019778	-0.452185	3.420492

8*R*,9*R*,11*R*-**1b**			Standard Orientation (Ångstroms)		
Center number	Atomic number	Atomic type	X	Y	Z
1.	6.	0.	0.554510	1.967060	-6.197531
2.	6.	0.	1.612560	2.473213	-5.445934
3.	6.	0.	1.659066	2.213186	-4.080272
4.	6.	0.	0.670381	1.460936	-3.432330
5.	6.	0.	-0.397110	0.954322	-4.212841
6.	6.	0.	-0.445524	1.210096	-5.587893
7.	6.	0.	0.872426	1.268579	-1.951031
8.	7.	0.	-0.084676	0.624909	-1.240143
9.	8.	0.	-1.360420	0.214466	-3.575139
10.	6.	0.	0.067708	0.427892	0.198198
11.	6.	0.	-1.270164	-0.030274	0.812905
12.	16.	0.	-1.756179	-1.780988	0.432352
13.	6.	0.	1.290014	-0.458923	0.558699
14.	8.	0.	2.378810	0.310961	1.034935
15.	6.	0.	-1.881300	1.312343	2.751718
16.	7.	0.	-1.214805	0.106594	2.270887
17.	6.	0.	-1.562870	-1.106467	3.031286
18.	6.	0.	-1.323476	-2.321887	2.130479
19.	6.	0.	-0.727060	-1.174595	4.328216
20.	6.	0.	-2.416655	-0.364908	-4.335031
21.	8.	0.	1.894351	1.710834	-1.402306

22.	7.	0.	0.690509	-1.026835	4.072724
23.	6.	0.	1.616263	-1.963921	4.455363
24.	6.	0.	3.051518	-1.629000	4.081254
25.	8.	0.	1.311281	-2.977885	5.074075
26.	1.	0.	-2.068993	0.596134	0.390265
27.	1.	0.	-2.630089	-1.087495	3.313975
28.	1.	0.	0.275950	1.408886	0.644927
29.	1.	0.	0.498392	2.155378	-7.265697
30.	1.	0.	2.393422	3.060974	-5.917128
31.	1.	0.	2.469722	2.584190	-3.463598
32.	1.	0.	-1.259050	0.823283	-6.188407
33.	1.	0.	-0.854185	0.205196	-1.744500
34.	1.	0.	1.032781	-1.145006	1.365225
35.	1.	0.	1.562435	-1.063216	-0.317554
36.	1.	0.	2.559639	0.953164	0.324000
37.	1.	0.	-1.691033	1.444357	3.820417
38.	1.	0.	-1.478966	2.191515	2.240792
39.	1.	0.	-2.974947	1.288509	2.598093
40.	1.	0.	-0.288084	-2.663546	2.177866
41.	1.	0.	-1.976320	-3.151214	2.408978
42.	1.	0.	-1.074243	-0.397954	5.023084
43.	1.	0.	-0.865177	-2.138344	4.824651
44.	1.	0.	-3.023551	-0.922916	-3.621548
45.	1.	0.	-3.032522	0.405035	-4.813513
46.	1.	0.	-2.028223	-1.050179	-5.096543
47.	1.	0.	0.977822	-0.249478	3.493647
48.	1.	0.	3.586125	-2.561460	3.892671
49.	1.	0.	3.535286	-1.135294	4.931343
50.	1.	0.	3.127651	-0.973226	3.208915

8*R*,9*R*,11*R*-**1c**			Standard Orientation (Ångstroms)		
Center number	Atomic number	Atomic type	X	Y	Z
1.	6.	0.	0.191849	1.648732	-4.574174
2.	6.	0.	0.994243	0.519531	-4.428321
3.	6.	0.	1.333772	0.089303	-3.149441
4.	6.	0.	0.899405	0.760035	-1.998141
5.	6.	0.	0.090727	1.909729	-2.168648
6.	6.	0.	-0.260616	2.341883	-3.452006
7.	6.	0.	1.368073	0.166469	-0.688008
8.	7.	0.	1.272882	0.936949	0.417155
9.	8.	0.	-0.330420	2.557155	-1.036815
10.	6.	0.	1.744953	0.678153	1.781297
11.	6.	0.	0.578195	0.474907	2.787227
12.	16.	0.	-0.710863	1.809932	2.663897
13.	6.	0.	2.779652	-0.457314	1.914535

14.	8.	0.	3.887003	-0.301183	1.056808
15.	6.	0.	0.152184	-1.723534	3.760755
16.	7.	0.	-0.107265	-0.816524	2.642084
17.	6.	0.	-1.551759	-0.698213	2.347106
18.	6.	0.	-1.787782	0.670557	1.711915
19.	6.	0.	-2.038337	-1.867597	1.477910
20.	6.	0.	-1.135729	3.727306	-1.150813
21.	8.	0.	1.855087	-0.980366	-0.686676
22.	7.	0.	-1.286117	-2.011859	0.248247
23.	6.	0.	-1.891321	-2.422587	-0.909945
24.	6.	0.	-0.954963	-2.681388	-2.078126
25.	8.	0.	-3.105408	-2.578274	-0.999591
26.	1.	0.	1.008717	0.566882	3.792985
27.	1.	0.	-2.125455	-0.733678	3.289748
28.	1.	0.	2.254056	1.602509	2.086032
29.	1.	0.	-0.089496	2.000123	-5.562489
30.	1.	0.	1.351454	-0.020804	-5.298683
31.	1.	0.	1.952053	-0.788274	-3.001416
32.	1.	0.	-0.886651	3.215481	-3.583069
33.	1.	0.	0.816125	1.832604	0.292959
34.	1.	0.	3.157405	-0.421176	2.944298
35.	1.	0.	2.286095	-1.421311	1.764450
36.	1.	0.	3.579332	-0.657877	0.206193
37.	1.	0.	-0.316130	-2.692172	3.566722
38.	1.	0.	1.225038	-1.898416	3.867359
39.	1.	0.	-0.239388	-1.339731	4.719265
40.	1.	0.	-1.512861	0.682473	0.656193
41.	1.	0.	-2.827100	0.985555	1.822746
42.	1.	0.	-1.994947	-2.793561	2.070427
43.	1.	0.	-3.086519	-1.726957	1.202187
44.	1.	0.	-1.323154	4.052081	-0.127432
45.	1.	0.	-0.611966	4.518871	-1.698497
46.	1.	0.	-2.087571	3.505859	-1.645968
47.	1.	0.	-0.277068	-1.947558	0.298885
48.	1.	0.	-1.299398	-2.103310	-2.939346
49.	1.	0.	-1.026789	-3.739435	-2.348133
50.	1.	0.	0.085773	-2.429978	-1.864647

8*R*,9*R*,11*R*-**1d**			Standard Orientation (Ångstroms)		
Center number	Atomic number	Atomic type	X	Y	Z
1.	6.	0.	1.782051	0.997963	-4.466791
2.	6.	0.	2.093190	2.291397	-4.043340
3.	6.	0.	1.988336	2.621871	-2.696423
4.	6.	0.	1.565030	1.684360	-1.741399
5.	6.	0.	1.266390	0.383508	-2.186318

6.	6.	0.	1.375098	0.044885	-3.535673
7.	6.	0.	1.478314	2.183738	-0.320290
8.	7.	0.	0.725953	1.474273	0.548267
9.	8.	0.	0.844244	-0.606694	-1.299133
10.	6.	0.	0.413644	1.926781	1.898874
11.	6.	0.	-0.810738	1.117811	2.407457
12.	16.	0.	-2.381893	1.845575	1.740049
13.	6.	0.	1.644819	1.936772	2.845384
14.	8.	0.	2.357451	3.147242	2.772231
15.	6.	0.	0.102274	-1.089402	2.827320
16.	7.	0.	-0.809106	-0.294967	1.997705
17.	6.	0.	-2.187770	-0.831545	1.994973
18.	6.	0.	-3.070819	0.207083	1.296653
19.	6.	0.	-2.275344	-2.204268	1.312570
20.	6.	0.	1.905070	-1.451343	-0.823243
21.	8.	0.	2.066675	3.226892	0.000800
22.	7.	0.	-1.814329	-2.178331	-0.063701
23.	6.	0.	-2.475543	-2.867853	-1.046602
24.	6.	0.	-1.915029	-2.701887	-2.449992
25.	8.	0.	-3.450657	-3.572745	-0.814202
26.	1.	0.	-0.848500	1.213139	3.503663
27.	1.	0.	-2.544310	-0.958113	3.033790
28.	1.	0.	0.104867	2.978964	1.854372
29.	1.	0.	1.856080	0.729444	-5.516231
30.	1.	0.	2.414773	3.039712	-4.760675
31.	1.	0.	2.232629	3.616470	-2.340820
32.	1.	0.	1.133089	-0.968481	-3.840422
33.	1.	0.	0.275607	0.619295	0.241540
34.	1.	0.	1.305123	1.833456	3.883445
35.	1.	0.	2.286811	1.067686	2.619123
36.	1.	0.	2.453486	3.355133	1.821962
37.	1.	0.	0.153571	-2.114693	2.454942
38.	1.	0.	1.109407	-0.673553	2.779049
39.	1.	0.	-0.209613	-1.121286	3.885570
40.	1.	0.	-3.046194	0.077088	0.213231
41.	1.	0.	-4.104437	0.133496	1.640189
42.	1.	0.	-1.723441	-2.950576	1.899189
43.	1.	0.	-3.315751	-2.539668	1.296087
44.	1.	0.	1.436018	-2.221545	-0.208855
45.	1.	0.	2.431865	-1.921282	-1.660357
46.	1.	0.	2.617467	-0.878021	-0.219956
47.	1.	0.	-1.040892	-1.572825	-0.306715
48.	1.	0.	-1.038001	-2.052517	-2.493198
49.	1.	0.	-2.699921	-2.293745	-3.092759

50.	1.	0.	-1.658043	-3.689691	-2.842339
8*R*,9*R*,11*R*-**1e**			Standard Orientation (Ångstroms)		
Center number	Atomic number	Atomic type	X	Y	Z
1.	6.	0.	1.841334	1.000773	-4.530311
2.	6.	0.	2.723909	1.919965	-3.959799
3.	6.	0.	2.590653	2.263486	-2.619004
4.	6.	0.	1.584140	1.707873	-1.813069
5.	6.	0.	0.698727	0.789647	-2.408646
6.	6.	0.	0.829473	0.440772	-3.754113
7.	6.	0.	1.575389	2.159649	-0.372182
8.	7.	0.	0.850671	1.435593	0.507912
9.	8.	0.	-0.320750	0.185056	-1.677763
10.	6.	0.	0.734960	1.753671	1.927839
11.	6.	0.	-0.307513	0.792255	2.555515
12.	16.	0.	-2.028127	1.390534	2.205465
13.	6.	0.	2.101194	1.811282	2.674968
14.	8.	0.	2.646947	3.106152	2.681883
15.	6.	0.	0.914896	-1.309782	2.522263
16.	7.	0.	-0.263504	-0.581363	2.040913
17.	6.	0.	-1.529609	-1.268708	2.367003
18.	6.	0.	-2.675386	-0.313950	1.999240
19.	6.	0.	-1.658793	-2.613539	1.639198
20.	6.	0.	-1.599159	0.833649	-1.817246
21.	8.	0.	2.253091	3.143517	-0.038511
22.	7.	0.	-1.478363	-2.476235	0.205606
23.	6.	0.	-2.095191	-3.327935	-0.672280
24.	6.	0.	-1.732028	-3.132001	-2.135867
25.	8.	0.	-2.882039	-4.192088	-0.304080
26.	1.	0.	-0.170589	0.813330	3.648147
27.	1.	0.	-1.578464	-1.475514	3.452083
28.	1.	0.	0.336515	2.771004	2.040157
29.	1.	0.	1.939381	0.718087	-5.574142
30.	1.	0.	3.514769	2.363149	-4.556569
31.	1.	0.	3.260386	2.976374	-2.151658
32.	1.	0.	0.132753	-0.277215	-4.175326
33.	1.	0.	0.331125	0.631274	0.173656
34.	1.	0.	1.947986	1.531554	3.723843
35.	1.	0.	2.792265	1.075262	2.232466
36.	1.	0.	2.688763	3.371367	1.742005
37.	1.	0.	0.944757	-2.306419	2.077506
38.	1.	0.	1.823121	-0.790741	2.215049
39.	1.	0.	0.927049	-1.418535	3.620530
40.	1.	0.	-2.986608	-0.463157	0.963532
41.	1.	0.	-3.536239	-0.472223	2.651691

42.	1.	0.	-0.949342	-3.339493	2.059134
43.	1.	0.	-2.654472	-3.031870	1.808748
44.	1.	0.	-2.320258	0.204626	-1.294991
45.	1.	0.	-1.580359	1.828191	-1.360531
46.	1.	0.	-1.876030	0.914160	-2.873520
47.	1.	0.	-0.798126	-1.805536	-0.127007
48.	1.	0.	-2.653970	-3.078671	-2.720229
49.	1.	0.	-1.182489	-4.013573	-2.479854
50.	1.	0.	-1.126848	-2.240704	-2.317653

Table S9. Gibbs free energies[a] and equilibrium populations[b] of low-energy conformers of 8*S*,9*R*,11*R*-**1**.

Conformers	In MeOH	
	G	*P* (%)
8*S*,9*R*,11*R*-**1a**	-957514.10475825	40.06
8*S*,9*R*,11*R*-**1b**	-957514.10601327	40.14
8*S*,9*R*,11*R*-**1c**	-957513.11329245	7.50
8*S*,9*R*,11*R*-**1d**	-957513.11454747	7.52
8*S*,9*R*,11*R*-**1e**	-957512.8466007	4.78

[a]B3LYP/6-31G(d, p), in kcal/mol; [b] from G values at 298.15K.

Table S10. Cartesian coordinates for the low-energy reoptimized MMFF conformers of 8*S*,9*R*,11*R*-**1** at B3LYP/6-31G(d,p) level of theory in gas.

8*S*,9*R*,11*R*-**1a**		Standard Orientation (Ångstroms)			
Center number	Atomic number	Atomic type	X	Y	Z
1.	6.	0.	3.625760	-0.460761	-3.073836
2.	6.	0.	2.647535	0.362598	-3.626938
3.	6.	0.	1.383482	0.399065	-3.045577
4.	6.	0.	1.060213	-0.378449	-1.926218
5.	6.	0.	2.065619	-1.211103	-1.381042
6.	6.	0.	3.341457	-1.242838	-1.954301
7.	6.	0.	-0.354493	-0.230064	-1.415485
8.	7.	0.	-0.865202	-1.267121	-0.685907
9.	8.	0.	1.734204	-1.954698	-0.277732
10.	6.	0.	-2.219664	-1.289873	-0.149618
11.	6.	0.	-2.290118	-0.928143	1.353900
12.	16.	0.	-1.050742	-1.835456	2.396467
13.	6.	0.	-2.846677	-2.661267	-0.446779
14.	8.	0.	-2.900987	-2.933602	-1.832954
15.	6.	0.	-3.315724	1.258141	1.623386
16.	7.	0.	-2.067105	0.506427	1.561902

17.	6.	0.	-1.113110	0.820606	2.633971
18.	6.	0.	-0.074073	-0.298956	2.623184
19.	6.	0.	-0.492571	2.211500	2.417980
20.	6.	0.	2.717045	-2.790951	0.326179
21.	8.	0.	-1.020592	0.764013	-1.716613
22.	7.	0.	-0.021895	2.387979	1.058910
23.	6.	0.	1.031243	3.211436	0.772853
24.	6.	0.	1.335414	3.384579	-0.706435
25.	8.	0.	1.688327	3.770484	1.646332
26.	1.	0.	-3.279348	-1.231578	1.729956
27.	1.	0.	-1.608977	0.825178	3.621987
28.	1.	0.	-2.779742	-0.527473	-0.698962
29.	1.	0.	4.620426	-0.500610	-3.507937
30.	1.	0.	2.865814	0.971062	-4.498283
31.	1.	0.	0.602381	1.036559	-3.444292
32.	1.	0.	4.116192	-1.871490	-1.533622
33.	1.	0.	-0.201591	-1.933556	-0.306444
34.	1.	0.	-2.294132	-3.445818	0.099396
35.	1.	0.	-3.879495	-2.678014	-0.083197
36.	1.	0.	-2.020128	-2.738808	-2.184815
37.	1.	0.	-3.109079	2.331229	1.622193
38.	1.	0.	-3.915961	1.021100	2.520401
39.	1.	0.	-3.920655	1.047189	0.737351
40.	1.	0.	0.637632	-0.175257	1.804552
41.	1.	0.	0.463008	-0.358717	3.571583
42.	1.	0.	-1.230456	2.983458	2.681352
43.	1.	0.	0.355912	2.361290	3.090833
44.	1.	0.	3.067344	-3.561982	-0.369577
45.	1.	0.	2.218775	-3.262401	1.173280
46.	1.	0.	3.570676	-2.204272	0.683193
47.	1.	0.	-0.575201	1.979392	0.313551
48.	1.	0.	2.360828	3.056311	-0.897656
49.	1.	0.	1.286468	4.450586	-0.946188
50.	1.	0.	0.652963	2.832073	-1.355286

8*S*,9*R*,11*R*-**1b**		Standard Orientation (Ångstroms)			
Center number	Atomic number	Atomic type	X	Y	Z
1.	6.	0.	3.734391	-0.473917	-2.938924
2.	6.	0.	2.783086	0.358482	-3.524506
3.	6.	0.	1.498582	0.401551	-2.990338
4.	6.	0.	1.128603	-0.378096	-1.887049
5.	6.	0.	2.107294	-1.219941	-1.308408
6.	6.	0.	3.403312	-1.258383	-1.834013
7.	6.	0.	-0.303064	-0.221825	-1.428598

8.	7.	0.	-0.847646	-1.258186	-0.722921
9.	8.	0.	1.729972	-1.965522	-0.221297
10.	6.	0.	-2.221268	-1.273639	-0.237522
11.	6.	0.	-2.345230	-0.917561	1.263873
12.	16.	0.	-1.151756	-1.837732	2.347929
13.	6.	0.	-2.846121	-2.639386	-0.563767
14.	8.	0.	-2.850469	-2.905752	-1.952166
15.	6.	0.	-3.365325	1.274615	1.504670
16.	7.	0.	-2.120416	0.514547	1.486300
17.	6.	0.	-1.204771	0.817745	2.594339
18.	6.	0.	-0.173736	-0.309019	2.617390
19.	6.	0.	-0.567098	2.205096	2.407489
20.	6.	0.	2.683841	-2.811172	0.415110
21.	8.	0.	-0.950663	0.778113	-1.750002
22.	7.	0.	-0.044763	2.383608	1.067684
23.	6.	0.	1.023700	3.201147	0.824617
24.	6.	0.	1.383914	3.378322	-0.641551
25.	8.	0.	1.651462	3.752113	1.724395
26.	1.	0.	-3.349819	-1.215687	1.601500
27.	1.	0.	-1.736944	0.821844	3.563292
28.	1.	0.	-2.755248	-0.505093	-0.804025
29.	1.	0.	4.744201	-0.519041	-3.335957
30.	1.	0.	3.037789	0.968827	-4.384577
31.	1.	0.	0.737251	1.046102	-3.415090
32.	1.	0.	4.157555	-1.894093	-1.387496
33.	1.	0.	-0.203170	-1.930757	-0.321901
34.	1.	0.	-2.319749	-3.429979	-0.000717
35.	1.	0.	-3.891890	-2.650354	-0.239062
36.	1.	0.	-1.955795	-2.715600	-2.270067
37.	1.	0.	-3.151540	2.346247	1.515931
38.	1.	0.	-4.000118	1.037999	2.377698
39.	1.	0.	-3.938317	1.071577	0.595844
40.	1.	0.	0.568762	-0.186960	1.826326
41.	1.	0.	0.327272	-0.376321	3.584834
42.	1.	0.	-1.308990	2.981166	2.646398
43.	1.	0.	0.256667	2.346275	3.112158
44.	1.	0.	3.054424	-3.581837	-0.270465
45.	1.	0.	2.151138	-3.282520	1.241053
46.	1.	0.	3.527636	-2.232012	0.806157
47.	1.	0.	-0.572878	1.982275	0.300437
48.	1.	0.	2.413749	3.044494	-0.795780
49.	1.	0.	1.350361	4.445559	-0.878480
50.	1.	0.	0.722822	2.832677	-1.317746

8*S*,9*R*,11*R*-**1c**	Standard Orientation (Ångstroms)

Center number	Atomic number	Atomic type	X	Y	Z
1.	6.	0.	3.556795	-1.224622	-3.070569
2.	6.	0.	2.976528	-2.486265	-3.214100
3.	6.	0.	1.702556	-2.722942	-2.708734
4.	6.	0.	0.978124	-1.721990	-2.042421
5.	6.	0.	1.576727	-0.455587	-1.916716
6.	6.	0.	2.852195	-0.209718	-2.427197
7.	6.	0.	-0.390677	-2.124586	-1.540105
8.	7.	0.	-0.904664	-1.403193	-0.520274
9.	8.	0.	0.920568	0.592917	-1.271648
10.	6.	0.	-2.213051	-1.569845	0.111788
11.	6.	0.	-2.213657	-0.765170	1.429822
12.	16.	0.	-1.188420	-1.564538	2.759235
13.	6.	0.	-2.657077	-3.033452	0.352012
14.	8.	0.	-3.266442	-3.620597	-0.768182
15.	6.	0.	-2.662255	1.468167	0.611954
16.	7.	0.	-1.679805	0.595541	1.261820
17.	6.	0.	-1.210204	1.126808	2.558628
18.	6.	0.	-0.381992	0.018124	3.211991
19.	6.	0.	-0.406460	2.425120	2.399324
20.	6.	0.	0.208769	1.467773	-2.161187
21.	8.	0.	-0.945990	-3.107402	-2.051472
22.	7.	0.	0.758975	2.265680	1.548712
23.	6.	0.	1.945504	2.880477	1.851371
24.	6.	0.	3.094443	2.585625	0.900395
25.	8.	0.	2.076403	3.619507	2.820242
26.	1.	0.	-3.253607	-0.740946	1.792792
27.	1.	0.	-2.075021	1.355293	3.208678
28.	1.	0.	-2.991507	-1.141441	-0.537639
29.	1.	0.	4.552201	-1.028928	-3.457578
30.	1.	0.	3.515701	-3.282821	-3.716876
31.	1.	0.	1.226675	-3.690730	-2.818643
32.	1.	0.	3.279474	0.781150	-2.308925
33.	1.	0.	-0.398435	-0.574591	-0.225751
34.	1.	0.	-1.794994	-3.609480	0.720600
35.	1.	0.	-3.411019	-3.023961	1.148374
36.	1.	0.	-2.562845	-3.642962	-1.446850
37.	1.	0.	-2.219454	2.443583	0.399907
38.	1.	0.	-3.562330	1.624328	1.231582
39.	1.	0.	-2.974345	1.035783	-0.340895
40.	1.	0.	0.645252	0.029641	2.843133
41.	1.	0.	-0.371463	0.125688	4.298293
42.	1.	0.	-1.059945	3.225688	2.027606

43.	1.	0.	-0.048188	2.756419	3.377638
44.	1.	0.	0.882809	1.883965	-2.917327
45.	1.	0.	-0.193203	2.274869	-1.546852
46.	1.	0.	-0.610183	0.936098	-2.658319
47.	1.	0.	0.703700	1.637842	0.756649
48.	1.	0.	2.820677	1.912942	0.084904
49.	1.	0.	3.916258	2.145931	1.472179

8*S*,9*R*,11*R*-**1d**		Standard Orientation (Ångstroms)			
Center number	Atomic number	Atomic type	X	Y	Z
1.	6.	0.	3.604391	-1.092219	-3.064984
2.	6.	0.	3.163921	-2.416834	-3.085389
3.	6.	0.	1.934782	-2.745522	-2.523589
4.	6.	0.	1.118641	-1.775190	-1.921079
5.	6.	0.	1.576406	-0.445389	-1.919401
6.	6.	0.	2.805888	-0.107883	-2.487133
7.	6.	0.	-0.186410	-2.278682	-1.345479
8.	7.	0.	-0.754824	-1.532258	-0.373673
9.	8.	0.	0.822441	0.577668	-1.344189
10.	6.	0.	-2.023031	-1.784552	0.309818
11.	6.	0.	-2.084407	-0.874226	1.555692
12.	16.	0.	-0.948873	-1.440462	2.914884
13.	6.	0.	-2.297389	-3.261499	0.684502
14.	8.	0.	-2.862120	-4.005339	-0.363683
15.	6.	0.	-2.794459	1.218637	0.568974
16.	7.	0.	-1.707914	0.516753	1.258765
17.	6.	0.	-1.272429	1.205396	2.491902
18.	6.	0.	-0.313033	0.253359	3.209644
19.	6.	0.	-0.620701	2.564587	2.200578
20.	6.	0.	-0.001615	1.289367	-2.281046
21.	8.	0.	-0.640873	-3.355916	-1.756358
22.	7.	0.	0.536775	2.459741	1.330827
23.	6.	0.	1.654340	3.223087	1.544874
24.	6.	0.	2.808237	2.973663	0.586926
25.	8.	0.	1.723449	4.052457	2.444524
26.	1.	0.	-3.112656	-0.931649	1.947138
27.	1.	0.	-2.143185	1.393955	3.146807
28.	1.	0.	-2.857702	-1.500474	-0.348890
29.	1.	0.	4.563728	-0.824069	-3.497043
30.	1.	0.	3.776934	-3.190697	-3.536328
31.	1.	0.	1.566347	-3.764935	-2.538120
32.	1.	0.	3.123601	0.929870	-2.464799
33.	1.	0.	-0.336807	-0.631667	-0.164426
34.	1.	0.	-1.369232	-3.706980	1.072992

35.	1.	0.	-3.030476	-3.265217	1.500148
36.	1.	0.	-2.175183	-4.009617	-1.059548
37.	1.	0.	-2.466790	2.214356	0.262954
38.	1.	0.	-3.692803	1.329190	1.200803
39.	1.	0.	-3.077162	0.674539	-0.334526
40.	1.	0.	0.698598	0.344361	2.809974
41.	1.	0.	-0.291487	0.454463	4.282409
42.	1.	0.	-1.366465	3.254676	1.783766
43.	1.	0.	-0.280415	3.015703	3.136482
44.	1.	0.	0.605110	1.708713	-3.090573
45.	1.	0.	-0.476639	2.098556	-1.724307
46.	1.	0.	-0.767591	0.630789	-2.705379
47.	1.	0.	0.534255	1.763882	0.595724
48.	1.	0.	2.593041	2.207670	-0.161021
49.	1.	0.	3.685594	2.676255	1.167827
50.	1.	0.	3.056193	3.913585	0.086029

8*S*,9*R*,11*R*-**1e**		Standard Orientation (Ångstroms)			
Center number	Atomic number	Atomic type	X	Y	Z
1.	6.	0.	3.746459	-1.071880	-2.821903
2.	6.	0.	2.726709	-1.224626	-3.758680
3.	6.	0.	1.412960	-1.337852	-3.316166
4.	6.	0.	1.078204	-1.297143	-1.955372
5.	6.	0.	2.129577	-1.152068	-1.016148
6.	6.	0.	3.453589	-1.036844	-1.458955
7.	6.	0.	-0.395933	-1.417917	-1.655624
8.	7.	0.	-0.814808	-1.258354	-0.379053
9.	8.	0.	1.803146	-1.135372	0.313372
10.	6.	0.	-2.225582	-1.300188	-0.001084
11.	6.	0.	-2.443650	-0.563475	1.338032
12.	16.	0.	-1.358112	-1.171174	2.719327
13.	6.	0.	-2.805610	-2.731746	-0.005144
14.	8.	0.	-3.179252	-3.161837	-1.295949
15.	6.	0.	-3.467139	1.602190	0.949675
16.	7.	0.	-2.219506	0.886111	1.205346
17.	6.	0.	-1.420139	1.469362	2.293344
18.	6.	0.	-0.421561	0.404192	2.735415
19.	6.	0.	-0.707105	2.758144	1.829772
20.	6.	0.	2.840019	-1.018236	1.290726
21.	8.	0.	-1.195127	-1.630516	-2.582898
22.	7.	0.	-0.010681	2.616901	0.566102
23.	6.	0.	1.351428	2.531328	0.463239
24.	6.	0.	1.878356	2.478895	-0.961301
25.	8.	0.	2.097805	2.560162	1.438809

26.	1.	0.	-3.474143	-0.769186	1.658360
27.	1.	0.	-2.059340	1.731970	3.156590
28.	1.	0.	-2.785446	-0.743589	-0.763384
29.	1.	0.	4.780073	-0.980671	-3.142895
30.	1.	0.	2.950768	-1.257093	-4.819807
31.	1.	0.	0.592919	-1.463637	-4.013875
32.	1.	0.	4.259562	-0.917701	-0.746276
33.	1.	0.	-0.118465	-1.240505	0.355501
34.	1.	0.	-2.072931	-3.409182	0.464946
35.	1.	0.	-3.713153	-2.758225	0.613382
36.	1.	0.	-2.539430	-2.780674	-1.926655
37.	1.	0.	-3.261824	2.651279	0.717974
38.	1.	0.	-4.163318	1.571351	1.807080
39.	1.	0.	-3.973241	1.172494	0.081018
40.	1.	0.	0.429085	0.360262	2.054736
41.	1.	0.	-0.058894	0.592146	3.747534
42.	1.	0.	-1.436958	3.572319	1.746224
43.	1.	0.	0.033568	3.055280	2.575574
44.	1.	0.	3.587910	-1.808418	1.168203
45.	1.	0.	2.346138	-1.139146	2.254626
46.	1.	0.	3.309856	-0.030302	1.247242
47.	1.	0.	-0.569419	2.346535	-0.229952
48.	1.	0.	1.160822	2.062810	-1.673509
49.	1.	0.	2.793408	1.887179	-0.988543
50.	1.	0.	2.125404	3.497645	-1.278976

Table S11. Gibbs free energies[a] and equilibrium populations[b] of low-energy conformers of 8*R*,9*S*,11*S*-**1**.

Conformers	In MeOH	
	G	*P* (%)
8*R*,9*S*,11*S*-**1a**	-957514.10350323	40.06
8*R*,9*S*,11*S*-**1b**	-957514.10413074	40.10
8*R*,9*S*,11*S*-**1c**	-957513.11329245	7.52
8*R*,9*S*,11*S*-**1d**	-957513.11454747	7.53
8*R*,9*S*,11*S*-**1e**	-957512.8466007	4.79

[a]B3LYP/6-31G(d, p), in kcal/mol; [b] from G values at 298.15K.

Table S12. Cartesian coordinates for the low-energy reoptimized MMFF conformers of 8*R*,9*S*,11*S*-**1** at B3LYP/6-31G(d, p) level of theory in gas.

8*R*,9*S*,11*S*-**1a**		Standard Orientation (Ångstroms)			
Center number	Atomic number	Atomic Type	X	Y	Z
1.	6.	0.	1.012856	4.007188	2.391960

2.	6.	0.	1.824875	3.920223	1.263326
3.	6.	0.	1.506486	3.001150	0.267794
4.	6.	0.	0.384457	2.167797	0.360320
5.	6.	0.	-0.430118	2.273291	1.512154
6.	6.	0.	-0.108178	3.187198	2.521371
7.	6.	0.	0.172622	1.227436	-0.803776
8.	7.	0.	-1.098798	0.770486	-1.011888
9.	8.	0.	-1.516304	1.440164	1.590757
10.	6.	0.	-1.471230	-0.116538	-2.106046
11.	6.	0.	-1.605264	-1.597653	-1.676299
12.	16.	0.	-2.612091	-1.837136	-0.135137
13.	6.	0.	-2.755756	0.416663	-2.760139
14.	8.	0.	-2.590953	1.724832	-3.270217
15.	6.	0.	0.228888	-2.890416	-2.608563
16.	7.	0.	-0.288976	-2.197413	-1.434070
17.	6.	0.	-0.194359	-2.962814	-0.183749
18.	6.	0.	-1.102658	-2.255522	0.820190
19.	6.	0.	1.266232	-3.048162	0.291334
20.	6.	0.	-2.355782	1.487891	2.740980
21.	8.	0.	1.110111	0.954980	-1.558381
22.	7.	0.	1.916996	-1.753592	0.272538
23.	6.	0.	2.924415	-1.451876	1.146076
24.	6.	0.	3.586212	-0.100468	0.930442
25.	8.	0.	3.270452	-2.214725	2.043621
26.	1.	0.	-2.142293	-2.134932	-2.473166
27.	1.	0.	-0.563323	-3.996170	-0.318436
28.	1.	0.	-0.660455	-0.057524	-2.838085
29.	1.	0.	1.245657	4.713370	3.183479
30.	1.	0.	2.696766	4.557563	1.159805
31.	1.	0.	2.122812	2.900232	-0.618459
32.	1.	0.	-0.723819	3.263614	3.408797
33.	1.	0.	-1.752527	0.862048	-0.241980
34.	1.	0.	-3.025161	-0.219555	-3.609743
35.	1.	0.	-3.587408	0.365585	-2.035660
36.	1.	0.	-2.178332	2.243748	-2.564432
37.	1.	0.	1.267467	-3.187125	-2.442601
38.	1.	0.	-0.353443	-3.793678	-2.865726
39.	1.	0.	0.221499	-2.217705	-3.470482
40.	1.	0.	-1.378699	-2.913468	1.646263
41.	1.	0.	-0.635847	-1.349546	1.211710
42.	1.	0.	1.315035	-3.435383	1.312496
43.	1.	0.	1.810414	-3.770331	-0.334838
44.	1.	0.	-3.129575	0.739692	2.569048
45.	1.	0.	-2.818311	2.474738	2.857054

46.	1.	0.	-1.797440	1.235684	3.649264
47.	1.	0.	1.686940	-1.123354	-0.488065
48.	1.	0.	3.173721	0.452372	0.084135
49.	1.	0.	4.656956	-0.261441	0.776056
50.	1.	0.	3.476230	0.496580	1.839969

8*R*,9*S*,11*S*-**1b**		Standard Orientation (Ångstroms)			
Center number	Atomic number	Atomic type	X	Y	Z
1.	6.	0.	1.720110	3.708951	2.468361
2.	6.	0.	1.459187	4.102231	1.157625
3.	6.	0.	0.683521	3.280931	0.344640
4.	6.	0.	0.145169	2.074143	0.809212
5.	6.	0.	0.418008	1.693504	2.144144
6.	6.	0.	1.206863	2.509556	2.962174
7.	6.	0.	-0.677897	1.298334	-0.193256
8.	7.	0.	-1.598728	0.419057	0.304297
9.	8.	0.	-0.106515	0.502744	2.576600
10.	6.	0.	-2.487719	-0.379870	-0.528835
11.	6.	0.	-2.011589	-1.842008	-0.707245
12.	16.	0.	-1.525749	-2.665926	0.883775
13.	6.	0.	-3.910685	-0.298804	0.046240
14.	8.	0.	-4.394721	1.028876	0.086404
15.	6.	0.	-1.241264	-2.181138	-2.986683
16.	7.	0.	-0.854640	-1.913713	-1.605785
17.	6.	0.	0.246198	-2.749675	-1.108283
18.	6.	0.	0.245805	-2.597029	0.411537
19.	6.	0.	1.579794	-2.328711	-1.749063
20.	6.	0.	0.158146	0.060277	3.904819
21.	8.	0.	-0.552039	1.518624	-1.400814
22.	7.	0.	1.778071	-0.894464	-1.691268
23.	6.	0.	3.031571	-0.352234	-1.629501
24.	6.	0.	3.084844	1.165717	-1.691089
25.	8.	0.	4.043865	-1.039472	-1.528470
26.	1.	0.	-2.855255	-2.427583	-1.103961
27.	1.	0.	0.080176	-3.815402	-1.350658
28.	1.	0.	-2.486822	0.090209	-1.516628
29.	1.	0.	2.327480	4.331703	3.118491
30.	1.	0.	1.856370	5.035098	0.771461
31.	1.	0.	0.470049	3.551778	-0.683248
32.	1.	0.	1.425899	2.216469	3.981246
33.	1.	0.	-1.493033	0.141546	1.273994
34.	1.	0.	-4.596776	-0.863505	-0.593745
35.	1.	0.	-3.932361	-0.768833	1.045107
36.	1.	0.	-3.697007	1.562263	0.494264
37.	1.	0.	-0.376921	-2.073883	-3.646707

38.	1.	0.	-1.661484	-3.193540	-3.126635
39.	1.	0.	-1.986081	-1.449960	-3.312475
40.	1.	0.	0.773307	-3.419167	0.898688
41.	1.	0.	0.685210	-1.645505	0.717315
42.	1.	0.	2.418981	-2.806022	-1.236217
43.	1.	0.	1.613111	-2.688220	-2.788014
44.	1.	0.	-0.354286	-0.896918	4.001335
45.	1.	0.	-0.236238	0.764487	4.646318
46.	1.	0.	1.232231	-0.080884	4.068381
47.	1.	0.	0.965356	-0.302353	-1.823673
48.	1.	0.	2.101312	1.628655	-1.792914
49.	1.	0.	3.715119	1.454721	-2.536949
50.	1.	0.	3.568057	1.539950	-0.784313

8*R*,9*S*,11*S*-**1c**			Standard Orientation (Ångstroms)		
Center number	Atomic number	Atomic type	X	Y	Z
1.	6.	0.	0.849227	3.970637	2.663000
2.	6.	0.	-0.440500	3.874227	3.188701
3.	6.	0.	-1.352588	2.988474	2.624828
4.	6.	0.	-1.007960	2.172047	1.536087
5.	6.	0.	0.293106	2.291283	1.015590
6.	6.	0.	1.211121	3.182739	1.572739
7.	6.	0.	-2.098417	1.252823	1.032158
8.	7.	0.	-1.706230	0.154545	0.350526
9.	8.	0.	0.711640	1.518229	-0.067569
10.	6.	0.	-2.562444	-0.859318	-0.264401
11.	6.	0.	-1.682349	-2.077670	-0.619040
12.	16.	0.	-1.171617	-3.054355	0.878516
13.	6.	0.	-3.795854	-1.286143	0.568386
14.	8.	0.	-4.900712	-0.436287	0.401259
15.	6.	0.	-0.695355	-1.347984	-2.705025
16.	7.	0.	-0.437701	-1.695763	-1.304427
17.	6.	0.	0.577938	-2.759103	-1.155189
18.	6.	0.	0.570439	-3.167952	0.319525
19.	6.	0.	1.969523	-2.304124	-1.617229
20.	6.	0.	0.581401	2.177816	-1.336936
21.	8.	0.	-3.276926	1.525003	1.302030
22.	7.	0.	2.450788	-1.140711	-0.894697
23.	6.	0.	3.764425	-1.033721	-0.520079
24.	6.	0.	4.117197	0.214253	0.273310
25.	8.	0.	4.601825	-1.882371	-0.803923
26.	1.	0.	-2.292876	-2.744656	-1.248497
27.	1.	0.	0.299981	-3.635755	-1.769109
28.	1.	0.	-2.978520	-0.466935	-1.204717
29.	1.	0.	1.571155	4.655702	3.097064

30.	1.	0.	-0.733556	4.486245	4.035782
31.	1.	0.	-2.363166	2.904771	3.008082
32.	1.	0.	2.205772	3.244348	1.142334
33.	1.	0.	-0.721289	0.066609	0.123040
34.	1.	0.	-4.107355	-2.276338	0.214560
35.	1.	0.	-3.493432	-1.391964	1.621095
36.	1.	0.	-4.602600	0.425971	0.753082
37.	1.	0.	0.215006	-0.968532	-3.173782
38.	1.	0.	-1.054307	-2.209716	-3.294117
39.	1.	0.	-1.447161	-0.558179	-2.762246
40.	1.	0.	0.928496	-4.191706	0.443846
41.	1.	0.	1.194792	-2.497859	0.913308
42.	1.	0.	2.691608	-3.107943	-1.450846
43.	1.	0.	1.962184	-2.121141	-2.700091
44.	1.	0.	1.005110	1.502975	-2.082222
45.	1.	0.	1.135096	3.122641	-1.341551
46.	1.	0.	-0.471542	2.373149	-1.568576
47.	1.	0.	1.786045	-0.431820	-0.611518
48.	1.	0.	3.264955	0.875587	0.443008
49.	1.	0.	4.900833	0.758376	-0.261192
50.	1.	0.	4.535792	-0.091402	1.236119

8*R*,9*S*,11*S*-**1d**		Standard Orientation (Ångstroms)			
Center number	Atomic number	Atomic type	X	Y	Z
1.	6.	0.	0.871475	2.788353	3.878814
2.	6.	0.	-0.161279	2.138889	4.557528
3.	6.	0.	-1.023453	1.297345	3.862618
4.	6.	0.	-0.878562	1.071444	2.484713
5.	6.	0.	0.162209	1.742505	1.818082
6.	6.	0.	1.026849	2.593280	2.508325
7.	6.	0.	-1.883594	0.126810	1.863783
8.	7.	0.	-1.520631	-0.483699	0.714789
9.	8.	0.	0.370117	1.574422	0.448959
10.	6.	0.	-2.322319	-1.404132	-0.090976
11.	6.	0.	-1.382741	-2.098977	-1.100453
12.	16.	0.	-0.277531	-3.362108	-0.300157
13.	6.	0.	-3.149912	-2.441983	0.705887
14.	8.	0.	-4.389459	-1.947964	1.142211
15.	6.	0.	-1.216924	-0.407181	-2.823295
16.	7.	0.	-0.492272	-1.151412	-1.788872
17.	6.	0.	0.697075	-1.853742	-2.314886
18.	6.	0.	1.206795	-2.755722	-1.188572
19.	6.	0.	1.778831	-0.880523	-2.804444
20.	6.	0.	-0.253899	2.582824	-0.361605
21.	8.	0.	-2.956056	-0.070791	2.452487

22.	7.	0.	2.234424	0.024863	-1.765252
23.	6.	0.	3.557482	0.362293	-1.650670
24.	6.	0.	3.898580	1.279540	-0.487198
25.	8.	0.	4.411007	-0.043622	-2.430880
26.	1.	0.	-2.021813	-2.636201	-1.819341
27.	1.	0.	0.412158	-2.486067	-3.176083
28.	1.	0.	-3.065366	-0.833984	-0.668978
29.	1.	0.	1.553107	3.444753	4.411188
30.	1.	0.	-0.293088	2.286899	5.624633
31.	1.	0.	-1.839784	0.791692	4.365684
32.	1.	0.	1.818081	3.092057	1.957376
33.	1.	0.	-0.650279	-0.194387	0.280771
34.	1.	0.	-3.367374	-3.279478	0.032076
35.	1.	0.	-2.531823	-2.832147	1.528437
36.	1.	0.	-4.161879	-1.226784	1.762079
37.	1.	0.	-0.574114	0.362699	-3.255215
38.	1.	0.	-1.572795	-1.058807	-3.640112
39.	1.	0.	-2.081918	0.095546	-2.385888
40.	1.	0.	1.765557	-3.603903	-1.588703
41.	1.	0.	1.847720	-2.198884	-0.502596
42.	1.	0.	2.651250	-1.444139	-3.145677
43.	1.	0.	1.413887	-0.325772	-3.679194
44.	1.	0.	0.042687	2.380281	-1.391850
45.	1.	0.	0.089760	3.581229	-0.071113
46.	1.	0.	-1.344895	2.534472	-0.272773
47.	1.	0.	1.567785	0.357145	-1.079844
48.	1.	0.	4.634829	0.778690	0.147418
49.	1.	0.	3.030956	1.556907	0.115109
50.	1.	0.	4.373137	2.182469	-0.881339

8*R*,9*S*,11*S*-**1e**		Standard Orientation (Ångstroms)			
Center number	Atomic number	Atomic type	X	Y	Z
1.	6.	0.	0.520300	4.199063	2.290299
2.	6.	0.	0.201866	4.687335	1.025007
3.	6.	0.	-0.363963	3.826574	0.090392
4.	6.	0.	-0.619899	2.478331	0.377126
5.	6.	0.	-0.299505	2.001765	1.672812
6.	6.	0.	0.271342	2.866148	2.615859
7.	6.	0.	-1.220933	1.686097	-0.758056
8.	7.	0.	-1.378213	0.350813	-0.606947
9.	8.	0.	-0.571118	0.690625	1.958464
10.	6.	0.	-1.896836	-0.507283	-1.669770
11.	6.	0.	-1.458309	-1.969592	-1.439816
12.	16.	0.	-1.900136	-2.635011	0.238778
13.	6.	0.	-3.420851	-0.357635	-1.871747

14.	8.	0.	-3.755618	0.760277	-2.664821
15.	6.	0.	0.329976	-2.561911	-2.969405
16.	7.	0.	-0.005200	-2.136346	-1.612470
17.	6.	0.	0.631167	-2.952116	-0.567257
18.	6.	0.	-0.134732	-2.697514	0.727250
19.	6.	0.	2.127125	-2.598833	-0.420384
20.	6.	0.	-0.286875	0.181767	3.263958
21.	8.	0.	-1.530638	2.264367	-1.813248
22.	7.	0.	2.383533	-1.174996	-0.326802
23.	6.	0.	2.728299	-0.547745	0.839790
24.	6.	0.	3.040191	0.932743	0.696282
25.	8.	0.	2.841984	-1.142122	1.909107
26.	1.	0.	-2.008548	-2.591130	-2.159377
27.	1.	0.	0.563325	-4.028780	-0.810002
28.	1.	0.	-1.432616	-0.181651	-2.609352
29.	1.	0.	0.963619	4.851554	3.036914
30.	1.	0.	0.389609	5.724908	0.769193
31.	1.	0.	-0.630722	4.170467	-0.902414
32.	1.	0.	0.525471	2.505317	3.604250
33.	1.	0.	-1.255762	-0.045029	0.316686
34.	1.	0.	-3.807408	-1.243973	-2.393176
35.	1.	0.	-3.905353	-0.327103	-0.881268
36.	1.	0.	-3.113686	1.466063	-2.459306
37.	1.	0.	1.413893	-2.545547	-3.115301
38.	1.	0.	-0.030892	-3.580079	-3.201499
39.	1.	0.	-0.104332	-1.869924	-3.695954
40.	1.	0.	0.008033	-3.509733	1.442203
41.	1.	0.	0.177107	-1.759361	1.187118
42.	1.	0.	2.524668	-3.059161	0.486900
43.	1.	0.	2.686488	-3.007288	-1.270575
44.	1.	0.	-0.686789	-0.831941	3.274420
45.	1.	0.	-0.788892	0.774407	4.035641
46.	1.	0.	0.792413	0.145932	3.444249
47.	1.	0.	2.073241	-0.608112	-1.102268
48.	1.	0.	2.532660	1.402219	-0.150610
49.	1.	0.	4.120297	1.049140	0.556193
50.	1.	0.	2.762218	1.453005	1.612933

Table S13. Gibbs free energies[a] and equilibrium populations[b] of low-energy conformers of 8*S*,9*S*,11*S*-**1**.

Conformers	In MeOH	
	G	*P* (%)
8*S*,9*S*,11*S*-**1a**	-957513.9447432	63.01
8*S*,9*S*,11*S*-**1b**	-957513.56886471	33.39
8*S*,9*S*,11*S*-**1c**	-957511.63864395	1.28
8*S*,9*S*,11*S*-**1d**	-957511.63487889	1.27
8*S*,9*S*,11*S*-**1e**	-957511.51690701	1.04

[a]B3LYP/6-31G(d, p), in kcal/mol; [b] from G values at 298.15K.

Table S14. Cartesian coordinates for the low-energy reoptimized MMFF conformers of 8*S*,9*S*,11*S*-**1** at B3LYP/6-31G(d, p) level of theory in gas.

8*S*,9*S*,11*S*-**1a**			Standard Orientation (Ångstroms)		
Center number	Atomic number	Atomic type	X	Y	Z
1.	6.	0.	-5.038120	1.649941	3.805391
2.	6.	0.	-4.423826	2.798781	3.312080
3.	6.	0.	-3.352335	2.670688	2.434403
4.	6.	0.	-2.872352	1.419846	2.024274
5.	6.	0.	-3.509099	0.263369	2.536640
6.	6.	0.	-4.584775	0.387749	3.423198
7.	6.	0.	-1.702963	1.456868	1.073901
8.	7.	0.	-1.238080	0.290113	0.565556
9.	8.	0.	-3.032664	-0.959217	2.136305
10.	6.	0.	-0.113814	0.284170	-0.365503
11.	6.	0.	0.010453	-1.099571	-1.034525
12.	16.	0.	0.662613	-2.450348	0.059059
13.	6.	0.	1.196586	0.823400	0.268801
14.	8.	0.	1.480788	2.145839	-0.150211
15.	6.	0.	0.166574	-0.921977	-3.459332
16.	7.	0.	0.896019	-1.006159	-2.198371
17.	6.	0.	2.010145	-1.970096	-2.221368
18.	6.	0.	2.291902	-2.411472	-0.781962
19.	6.	0.	3.249678	-1.328700	-2.882605
20.	6.	0.	-3.606394	-2.145946	2.675950
21.	8.	0.	-1.194745	2.544629	0.758521
22.	7.	0.	3.587875	-0.053138	-2.286609
23.	6.	0.	4.820130	0.210625	-1.744837
24.	6.	0.	4.970660	1.604863	-1.157034
25.	8.	0.	5.735039	-0.605883	-1.758647
26.	1.	0.	-0.997229	-1.422569	-1.333403

27.	1.	0.	1.732229	-2.861536	-2.810701
28.	1.	0.	-0.355016	0.982599	-1.177445
29.	1.	0.	-5.874966	1.725371	4.493572
30.	1.	0.	-4.772562	3.782466	3.608693
31.	1.	0.	-2.844514	3.541025	2.034740
32.	1.	0.	-5.073203	-0.493877	3.818474
33.	1.	0.	-1.624043	-0.574257	0.921343
34.	1.	0.	1.122704	0.742743	1.362063
35.	1.	0.	2.045750	0.219339	-0.049609
36.	1.	0.	0.689390	2.660149	0.094020
37.	1.	0.	-0.551783	-0.098315	-3.421962
38.	1.	0.	0.858874	-0.710056	-4.278826
39.	1.	0.	-0.379038	-1.851189	-3.702449
40.	1.	0.	2.721295	-3.414727	-0.756264
41.	1.	0.	2.975112	-1.727908	-0.275022
42.	1.	0.	4.122989	-1.976395	-2.772301
43.	1.	0.	3.065225	-1.211959	-3.959142
44.	1.	0.	-3.046197	-2.972019	2.237168
45.	1.	0.	-4.663910	-2.237321	2.403262
46.	1.	0.	-3.504441	-2.175851	3.766411
47.	1.	0.	2.841442	0.620349	-2.179689
48.	1.	0.	4.021183	2.040317	-0.831975
49.	1.	0.	5.415820	2.263239	-1.911211
50.	1.	0.	5.662592	1.552010	-0.314820

8*S*,9*S*,11*S*-**1b**			Standard Orientation (Ångstroms)		
Center number	Atomic number	Atomic type	X	Y	Z
1.	6.	0.	-6.089560	0.623072	2.509083
2.	6.	0.	-6.185991	0.663612	1.119901
3.	6.	0.	-5.021669	0.639889	0.359407
4.	6.	0.	-3.750274	0.583265	0.945587
5.	6.	0.	-3.672503	0.541173	2.358655
6.	6.	0.	-4.841667	0.559927	3.128164
7.	6.	0.	-2.596885	0.558692	-0.030400
8.	7.	0.	-1.324882	0.662674	0.470559
9.	8.	0.	-2.426311	0.477731	2.928867
10.	6.	0.	-0.160434	0.534099	-0.398165
11.	6.	0.	0.244150	-0.947927	-0.577706
12.	16.	0.	0.937081	-1.739502	0.954192
13.	6.	0.	0.947279	1.462030	0.108202
14.	8.	0.	0.578803	2.826903	-0.004776
15.	6.	0.	0.603074	-1.406470	-2.931614
16.	7.	0.	1.242567	-1.098961	-1.650625
17.	6.	0.	2.334734	-2.024821	-1.300684
18.	6.	0.	2.600520	-1.845576	0.193080

19.	6.	0.	3.585353	-1.756245	-2.157350
20.	6.	0.	-2.307135	0.373693	4.344075
21.	8.	0.	-2.806670	0.480609	-1.241017
22.	7.	0.	3.985097	-0.364302	-2.119458
23.	6.	0.	5.275383	0.029310	-1.887560
24.	6.	0.	5.505866	1.532699	-1.888436
25.	8.	0.	6.187996	-0.767152	-1.701201
26.	1.	0.	-0.674491	-1.501783	-0.811403
27.	1.	0.	2.029382	-3.072163	-1.477444
28.	1.	0.	-0.473613	0.893863	-1.382515
29.	1.	0.	-6.984771	0.638816	3.123905
30.	1.	0.	-7.155238	0.711206	0.634498
31.	1.	0.	-5.055116	0.662147	-0.724014
32.	1.	0.	-4.786787	0.526157	4.208880
33.	1.	0.	-1.204511	0.546429	1.469558
34.	1.	0.	1.212240	1.199713	1.144563
35.	1.	0.	1.840297	1.322397	-0.505108
36.	1.	0.	-0.300558	2.907016	0.392036
37.	1.	0.	-0.201465	-0.692769	-3.123965
38.	1.	0.	1.325641	-1.320147	-3.748021
39.	1.	0.	0.173019	-2.423027	-2.955739
40.	1.	0.	3.127083	-2.704967	0.612045
41.	1.	0.	3.180476	-0.940729	0.388683
42.	1.	0.	4.430822	-2.344103	-1.792006
43.	1.	0.	3.397298	-2.076013	-3.191204
44.	1.	0.	-1.238030	0.307244	4.547171
45.	1.	0.	-2.804988	-0.526991	4.720195
46.	1.	0.	-2.718937	1.257135	4.845150
47.	1.	0.	3.254725	0.321342	-2.243883
48.	1.	0.	4.587099	2.121490	-1.956430
49.	1.	0.	6.152995	1.788517	-2.732493
50.	1.	0.	6.040506	1.804800	-0.975241

8*S*,9*S*,11*S*-**1c**			Standard Orientation (Ångstroms)		
Center number	Atomic number	Atomic type	X	Y	Z
1.	6.	0.	-2.901039	2.567947	3.147517
2.	6.	0.	-2.604653	3.741132	2.451003
3.	6.	0.	-1.910432	3.672554	1.248151
4.	6.	0.	-1.494331	2.445610	0.707077
5.	6.	0.	-1.810232	1.273320	1.419633
6.	6.	0.	-2.505893	1.337378	2.628355
7.	6.	0.	-0.732607	2.528683	-0.594120
8.	7.	0.	-0.044392	1.435786	-0.989097
9.	8.	0.	-1.426097	0.016914	0.958013
10.	6.	0.	0.727300	1.359423	-2.225788

11.	6.	0.	1.224176	-0.099747	-2.396554
12.	16.	0.	-0.128607	-1.164000	-3.088069
13.	6.	0.	1.839342	2.444599	-2.343652
14.	8.	0.	1.381111	3.604522	-2.991008
15.	6.	0.	2.906643	-0.229055	-0.645535
16.	7.	0.	1.635435	-0.757682	-1.150929
17.	6.	0.	1.668630	-2.220126	-1.355772
18.	6.	0.	0.368116	-2.610345	-2.074042
19.	6.	0.	1.835533	-2.982888	-0.034655
20.	6.	0.	-2.458107	-0.674800	0.229253
21.	8.	0.	-0.730858	3.592681	-1.231726
22.	7.	0.	0.832800	-2.603432	0.943672
23.	6.	0.	0.400675	-3.487238	1.897006
24.	6.	0.	-0.571696	-2.920219	2.919461
25.	8.	0.	0.775832	-4.653317	1.928214
26.	1.	0.	2.043000	-0.092998	-3.133111
27.	1.	0.	2.523126	-2.491502	-2.002897
28.	1.	0.	0.068127	1.572050	-3.078203
29.	1.	0.	-3.435687	2.608304	4.091778
30.	1.	0.	-2.909608	4.704845	2.846306
31.	1.	0.	-1.669686	4.567482	0.685751
32.	1.	0.	-2.723606	0.412256	3.152839
33.	1.	0.	-0.073631	0.601111	-0.413703
34.	1.	0.	2.244570	2.664310	-1.342312
35.	1.	0.	2.660979	2.050760	-2.953311
36.	1.	0.	0.593502	3.885913	-2.485077
37.	1.	0.	2.816891	0.842110	-0.463283
38.	1.	0.	3.154171	-0.700506	0.307597
39.	1.	0.	3.744022	-0.397346	-1.344571
40.	1.	0.	0.524259	-3.482599	-2.711691
41.	1.	0.	-0.419278	-2.837968	-1.352935
42.	1.	0.	1.734410	-4.056848	-0.212011
43.	1.	0.	2.849609	-2.834829	0.360826
44.	1.	0.	-2.072152	-1.673839	0.025875
45.	1.	0.	-2.673284	-0.164715	-0.714962
46.	1.	0.	-3.370588	-0.746556	0.830148
47.	1.	0.	0.540250	-1.635661	0.975520
48.	1.	0.	-0.913639	-1.910506	2.680099
49.	1.	0.	-0.081875	-2.909057	3.897957
50.	1.	0.	-1.429530	-3.592913	2.995961

8*S*,9*S*,11*S*-**1d**			Standard Orientation (Ångstroms)		
Center number	Atomic number	Atomic type	X	Y	Z
1.	6.	0.	-3.500635	0.674463	3.311936
2.	6.	0.	-2.858274	1.877579	3.028920

3.	6.	0.	-2.060370	1.969591	1.892791
4.	6.	0.	-1.885334	0.890271	1.016160
5.	6.	0.	-2.552825	-0.321356	1.318229
6.	6.	0.	-3.349642	-0.421974	2.463727
7.	6.	0.	-0.995016	1.162983	-0.176058
8.	7.	0.	-1.055089	0.298621	-1.212093
9.	8.	0.	-2.373638	-1.371190	0.456080
10.	6.	0.	-0.406265	0.366321	-2.525545
11.	6.	0.	0.684850	-0.724859	-2.708117
12.	16.	0.	0.083347	-2.420883	-2.238191
13.	6.	0.	0.142888	1.753010	-2.916829
14.	8.	0.	-0.818716	2.778362	-2.815661
15.	6.	0.	3.046284	-0.110470	-2.800816
16.	7.	0.	1.913080	-0.464060	-1.945115
17.	6.	0.	2.257520	-1.531590	-0.981106
18.	6.	0.	0.973224	-2.284592	-0.640147
19.	6.	0.	2.974714	-0.957417	0.250033
20.	6.	0.	-3.032522	-2.609965	0.705223
21.	8.	0.	-0.280311	2.183562	-0.172771
22.	7.	0.	2.220248	0.088905	0.909343
23.	6.	0.	2.249845	0.234987	2.271008
24.	6.	0.	1.540893	1.466002	2.809888
25.	8.	0.	2.814853	-0.567769	3.007642
26.	1.	0.	0.906563	-0.773207	-3.782301
27.	1.	0.	2.950403	-2.248373	-1.455548
28.	1.	0.	-1.199751	0.129278	-3.247123
29.	1.	0.	-4.124693	0.578249	4.195503
30.	1.	0.	-2.975721	2.734284	3.684172
31.	1.	0.	-1.542955	2.889460	1.646938
32.	1.	0.	-3.852952	-1.350907	2.700455
33.	1.	0.	-1.639922	-0.516854	-1.073422
34.	1.	0.	1.033131	1.974959	-2.322166
35.	1.	0.	0.440969	1.688671	-3.971073
36.	1.	0.	-0.807940	3.020486	-1.874019
37.	1.	0.	2.807227	0.767788	-3.404527
38.	1.	0.	3.909782	0.147317	-2.181830
39.	1.	0.	3.342224	-0.932519	-3.476118
40.	1.	0.	1.188590	-3.286807	-0.264815
41.	1.	0.	0.365453	-1.744614	0.087392
42.	1.	0.	3.152523	-1.743373	0.988373
43.	1.	0.	3.965279	-0.588496	-0.055021
44.	1.	0.	-2.741094	-3.264587	-0.116029
45.	1.	0.	-4.121475	-2.487418	0.710091
46.	1.	0.	-2.707664	-3.046685	1.655993

47.	1.	0.	1.791110	0.800659	0.331314
48.	1.	0.	2.282014	2.099070	3.307360
49.	1.	0.	0.820684	1.152300	3.569762
50.	1.	0.	1.027106	2.043785	2.039108

8*S*,9*S*,11*S*-**1e**			Standard Orientation (Ångstroms)		
Center number	Atomic number	Atomic type	X	Y	Z
1.	6.	0.	-2.949385	1.978717	3.326534
2.	6.	0.	-1.683810	2.556993	3.390492
3.	6.	0.	-0.799247	2.371918	2.332553
4.	6.	0.	-1.142026	1.625689	1.196910
5.	6.	0.	-2.435807	1.052437	1.148666
6.	6.	0.	-3.326217	1.228287	2.213285
7.	6.	0.	-0.068149	1.532825	0.135476
8.	7.	0.	-0.441255	1.128251	-1.097892
9.	8.	0.	-2.760365	0.322764	0.035179
10.	6.	0.	0.343164	1.053130	-2.334704
11.	6.	0.	0.593028	-0.410069	-2.794294
12.	16.	0.	-0.963612	-1.427779	-2.812416
13.	6.	0.	1.662417	1.851311	-2.325820
14.	8.	0.	1.490487	3.201577	-1.960517
15.	6.	0.	2.833109	-1.375805	-2.686558
16.	7.	0.	1.579734	-1.125654	-1.973877
17.	6.	0.	1.051545	-2.349882	-1.333618
18.	6.	0.	-0.464994	-2.203592	-1.227006
19.	6.	0.	1.736984	-2.610213	0.016245
20.	6.	0.	-4.054482	-0.264189	-0.071954
21.	8.	0.	1.096100	1.868169	0.426103
22.	7.	0.	1.641353	-1.487557	0.926848
23.	6.	0.	1.509506	-1.677874	2.276947
24.	6.	0.	1.580321	-0.417583	3.122569
25.	8.	0.	1.351434	-2.789115	2.773344
26.	1.	0.	0.929770	-0.360018	-3.838068
27.	1.	0.	1.259651	-3.222605	-1.977051
28.	1.	0.	-0.294035	1.510598	-3.103592
29.	1.	0.	-3.654614	2.106019	4.142527
30.	1.	0.	-1.387589	3.144574	4.253100
31.	1.	0.	0.194333	2.803990	2.353488
32.	1.	0.	-4.312332	0.782380	2.181373
33.	1.	0.	-1.407359	0.839144	-1.194320
34.	1.	0.	2.389408	1.348826	-1.682076
35.	1.	0.	2.046551	1.838139	-3.353769
36.	1.	0.	1.475108	3.183353	-0.988469
37.	1.	0.	3.269245	-0.435211	-3.030104
38.	1.	0.	3.555836	-1.839989	-2.010235

39.	1.	0.	2.701747	-2.042260	-3.557154
40.	1.	0.	-0.952084	-3.176378	-1.136809
41.	1.	0.	-0.755028	-1.570390	-0.387242
42.	1.	0.	1.283482	-3.471024	0.514068
43.	1.	0.	2.789409	-2.875147	-0.164254
44.	1.	0.	-4.065249	-0.770174	-1.037243
45.	1.	0.	-4.840651	0.498823	-0.047108
46.	1.	0.	-4.225392	-0.993949	0.727093
47.	1.	0.	1.825250	-0.562130	0.559938
48.	1.	0.	1.651646	0.498126	2.532387
49.	1.	0.	2.449742	-0.495031	3.782580
50.	1.	0.	0.693322	-0.371422	3.759446

Table S15. Gibbs free energies[a] and equilibrium populations[b] of low-energy conformers of *R*-OR-**2**.

Conformers	In MeOH	
	Boltzmann population (%)	Optical rotation (OR)
R-OR-**2a**	39.41	–39.19
R-OR-**2b**	39.45	–39.13
R-OR-**2c**	7.05	+454.65
R-OR-**2d**	7.05	+454.4
R-OR-**2e**	7.05	+54.79
Average		+37.06

[a] B3LYP/6-31+G(d, p), in kcal/mol; [b] from Δ*G* values at 298.15K.

Table S16. Cartesian coordinates for the low-energy reoptimized MMFF conformers of *R*-OR-**2** at B3LYP/6-311+G(d, p) level of theory in MeOH.

R-OR-**2a**	Standard Orientation (Ångstroms)				
No.	Atom	Type	X	Y	Z
1.	6.	0.	-1.599023	1.262045	-0.125064
2.	6.	0.	-0.456598	1.201579	-0.934898
3.	6.	0.	-0.585121	0.693234	-2.236338
4.	6.	0.	-1.820887	0.255123	-2.711921
5.	6.	0.	-2.950117	0.317327	-1.893611
6.	6.	0.	-2.835990	0.822600	-0.598100
7.	7.	0.	1.121819	0.115264	1.579035
8.	6.	0.	1.714889	0.552055	0.317085
9.	6.	0.	0.447363	-1.065449	1.762767
10.	6.	0.	0.575496	-2.087835	0.668425
11.	7.	0.	1.432041	-1.786690	-0.400425
12.	6.	0.	2.012874	-0.579561	-0.660661
13.	6.	0.	-0.077229	-3.251378	0.769988

14.	8.	0.	-0.183445	-1.312424	2.785594
15.	6.	0.	1.114214	1.075913	2.676931
16.	6.	0.	0.890952	1.655291	-0.420227
17.	8.	0.	2.712291	-0.387160	-1.645361
18.	1.	0.	-1.523922	1.662488	0.882479
19.	1.	0.	0.292248	0.640312	-2.875118
20.	1.	0.	-1.902354	-0.130479	-3.724207
21.	1.	0.	-3.912995	-0.021391	-2.264630
22.	1.	0.	-3.709357	0.877908	0.045064
23.	1.	0.	2.692469	0.993497	0.548173
24.	1.	0.	1.608993	-2.499961	-1.098017
25.	1.	0.	0.001012	-4.023580	0.012193
26.	1.	0.	-0.695617	-3.425900	1.639492
27.	1.	0.	2.070919	1.606645	2.698578
28.	1.	0.	0.307095	1.811881	2.580086
29.	1.	0.	0.966997	0.532254	3.608989
30.	1.	0.	0.773440	2.502089	0.263876
31.	1.	0.	1.518543	1.994304	-1.249177

R-OR-**2b**			Standard Orientation (Ångstroms)		
No.	Atom	Type	X	Y	Z
1.	6.	0.	-1.816810	0.829405	-0.419961
2.	6.	0.	-0.614451	0.929543	-1.133363
3.	6.	0.	-0.498312	0.238963	-2.349204
4.	6.	0.	-1.554130	-0.532088	-2.834986
5.	6.	0.	-2.744902	-0.626373	-2.112617
6.	6.	0.	-2.873744	0.057130	-0.903108
7.	7.	0.	0.906744	0.578260	1.615123
8.	6.	0.	1.511463	0.971038	0.344246
9.	6.	0.	0.493396	-0.691944	1.928907
10.	6.	0.	0.951021	-1.791421	1.011852
11.	7.	0.	1.822336	-1.443021	-0.030386
12.	6.	0.	2.146342	-0.179077	-0.430069
13.	6.	0.	0.563070	-3.050816	1.243368
14.	8.	0.	-0.166049	-0.941867	2.932787
15.	6.	0.	0.579248	1.646646	2.553000
16.	6.	0.	0.542554	1.746679	-0.603924
17.	8.	0.	2.880421	0.039105	-1.383665
18.	1.	0.	-1.931897	1.365010	0.518665
19.	1.	0.	0.428196	0.307387	-2.912694
20.	1.	0.	-1.447980	-1.055512	-3.780927
21.	1.	0.	-3.568122	-1.224642	-2.491961
22.	1.	0.	-3.797313	-0.007533	-0.335369
23.	1.	0.	2.340283	1.653340	0.571084
24.	1.	0.	2.219906	-2.181106	-0.599486

25.	1.	0.	0.883877	-3.876525	0.617312
26.	1.	0.	-0.085080	-3.248064	2.085928
27.	1.	0.	-0.356221	2.156228	2.292839
28.	1.	0.	0.463415	1.210960	3.544326
29.	1.	0.	1.388911	2.382747	2.558379
30.	1.	0.	0.174483	2.625511	-0.064425
31.	1.	0.	1.159343	2.110037	-1.430670

R-OR-**2c**			Standard Orientation (Ångstroms)		
No.	Atom	Type	X	Y	Z
1.	6.	0.	1.120716	2.479049	-0.523304
2.	6.	0.	0.049866	1.595251	-0.721253
3.	6.	0.	-1.245679	2.039752	-0.428962
4.	6.	0.	-1.467174	3.328723	0.059689
5.	6.	0.	-0.392412	4.194997	0.261795
6.	6.	0.	0.903480	3.766408	-0.033653
7.	7.	0.	0.201907	-1.115981	0.928731
8.	6.	0.	0.996630	-0.775820	-0.252540
9.	6.	0.	-0.662961	-2.177800	0.970600
10.	6.	0.	-0.501316	-3.212858	-0.111665
11.	7.	0.	0.620406	-3.096588	-0.947461
12.	6.	0.	1.436422	-2.003397	-1.047730
13.	6.	0.	-1.370205	-4.227254	-0.195406
14.	8.	0.	-1.488976	-2.326375	1.865937
15.	6.	0.	0.177454	-0.181911	2.054889
16.	6.	0.	0.295269	0.199045	-1.249944
17.	8.	0.	2.411882	-1.974936	-1.781963
18.	1.	0.	2.132043	2.158898	-0.763663
19.	1.	0.	-2.088060	1.370739	-0.585756
20.	1.	0.	-2.479548	3.654914	0.279616
21.	1.	0.	-0.562681	5.198329	0.640653
22.	1.	0.	1.745133	4.437630	0.111002
23.	1.	0.	1.921551	-0.304902	0.093375
24.	1.	0.	0.876417	-3.879424	-1.537439
25.	1.	0.	-1.275320	-5.007658	-0.942911
26.	1.	0.	-2.180405	-4.276823	0.519110
27.	1.	0.	1.167769	0.267145	2.164164
28.	1.	0.	-0.555715	0.616703	1.913164
29.	1.	0.	-0.082034	-0.737759	2.955745
30.	1.	0.	0.943894	0.246911	-2.131818
31.	1.	0.	-0.650353	-0.255006	-1.566000

R-OR-**2d**			Standard Orientation (Ångstroms)		
No.	Atom	Type	X	Y	Z
1.	6.	0.	1.247654	2.473829	0.007259
2.	6.	0.	0.368010	1.590147	-0.634854

3.	6.	0.	-0.922841	2.038328	-0.941957
4.	6.	0.	-1.330628	3.330848	-0.606727
5.	6.	0.	-0.449355	4.197036	0.040940
6.	6.	0.	0.843250	3.764750	0.345248
7.	7.	0.	-0.229975	-1.109984	0.929665
8.	6.	0.	1.003408	-0.779151	0.214071
9.	6.	0.	-1.030797	-2.169705	0.593729
10.	6.	0.	-0.416259	-3.212171	-0.303133
11.	7.	0.	0.958753	-3.103594	-0.563812
12.	6.	0.	1.741461	-2.012917	-0.301515
13.	6.	0.	-1.165348	-4.225412	-0.753893
14.	8.	0.	-2.166241	-2.310688	1.037467
15.	6.	0.	-0.740894	-0.168406	1.926517
16.	6.	0.	0.814348	0.190070	-0.994969
17.	8.	0.	2.940209	-1.991439	-0.533816
18.	1.	0.	2.260760	2.150724	0.235893
19.	1.	0.	-1.614234	1.369401	-1.448198
20.	1.	0.	-2.335706	3.659858	-0.854197
21.	1.	0.	-0.764186	5.203126	0.301450
22.	1.	0.	1.539314	4.435811	0.840241
23.	1.	0.	1.685454	-0.307549	0.927723
24.	1.	0.	1.443763	-3.891049	-0.977485
25.	1.	0.	-0.756224	-5.010866	-1.380370
26.	1.	0.	-2.206872	-4.268755	-0.466196
27.	1.	0.	0.103583	0.279314	2.456345
28.	1.	0.	-1.334083	0.630724	1.473839
29.	1.	0.	-1.371452	-0.717726	2.625443
30.	1.	0.	1.784095	0.231069	-1.503538
31.	1.	0.	0.101033	-0.264624	-1.691172

R-OR-**2e**			Standard Orientation (Ångstroms)		
No.	Atom	Type	X	Y	Z
1.	6.	0.	1.220253	0.844805	-2.339418
2.	6.	0.	0.026523	0.594042	-1.647218
3.	6.	0.	-0.920324	1.621834	-1.554628
4.	6.	0.	-0.678338	2.871954	-2.127280
5.	6.	0.	0.517472	3.111972	-2.804640
6.	6.	0.	1.466151	2.092649	-2.910954
7.	7.	0.	0.371659	-0.256290	1.394676
8.	6.	0.	0.638914	-1.095687	0.225624
9.	6.	0.	-0.570049	-0.565544	2.340573
10.	6.	0.	-1.070983	-1.986057	2.343632
11.	7.	0.	-0.407481	-2.901534	1.511757
12.	6.	0.	0.472054	-2.586461	0.512660
13.	6.	0.	-2.060890	-2.339272	3.172063

14.	8.	0.	-0.951577	0.238708	3.185342
15.	6.	0.	0.994799	1.065523	1.470840
16.	6.	0.	-0.230408	-0.762022	-1.027391
17.	8.	0.	1.033098	-3.435317	-0.162687
18.	1.	0.	1.959049	0.052808	-2.439293
19.	1.	0.	-1.855107	1.441311	-1.029883
20.	1.	0.	-1.425615	3.655864	-2.045223
21.	1.	0.	0.707086	4.083223	-3.251761
22.	1.	0.	2.395546	2.267496	-3.445275
23.	1.	0.	1.691372	-0.965770	-0.043321
24.	1.	0.	-0.570789	-3.892676	1.643710
25.	1.	0.	-2.433563	-3.356691	3.224604
26.	1.	0.	-2.487024	-1.587111	3.821747
27.	1.	0.	2.001387	1.007353	1.049278
28.	1.	0.	0.427807	1.821651	0.921115
29.	1.	0.	1.045273	1.360879	2.518725
30.	1.	0.	-0.018685	-1.548918	-1.759977
31.	1.	0.	-1.285612	-0.853720	-0.747398

Table S17. Gibbs free energies[a] and equilibrium populations[b] of low-energy conformers of *S*-OR-**2**.

Conformers	In MeOH	
	Boltzmann population (%)	Optical rotation (OR)
S-OR-**2a**	37.36	+39.18
S-OR-**2b**	37.6	+38.86
S-OR-**2c**	6.69	–454.66
S-OR-**2d**	6.69	–454.35
S-OR-**2e**	11.67	–3.37
Average		–31.95

[a] B3LYP/6-31+G(d, p), in kcal/mol; [b] from ΔG values at 298.15K.

Table S18. Cartesian coordinates for the low-energy reoptimized MMFF conformers of *S*-OR-**2** at B3LYP/6-311+G(d, p) level of theory in MeOH.

S-OR-**2a**			Standard Orientation (Ångstroms)		
No.	Atom	Type	X	Y	Z
1.	6.	0.	-2.258299	0.633734	-0.567947
2.	6.	0.	-1.089745	0.120436	-1.150770
3.	6.	0.	-0.960700	-1.269538	-1.277041
4.	6.	0.	-1.967565	-2.125622	-0.829486
5.	6.	0.	-3.123946	-1.602659	-0.249858
6.	6.	0.	-3.266829	-0.219987	-0.121552
7.	7.	0.	1.921245	0.192720	-0.191577
8.	6.	0.	1.105120	1.352589	-0.543257
9.	6.	0.	1.795914	-0.534044	0.965504
10.	6.	0.	0.957144	0.076675	2.053053
11.	7.	0.	0.445483	1.363129	1.829362
12.	6.	0.	0.456753	2.047892	0.649014
13.	6.	0.	0.770750	-0.589690	3.198222
14.	8.	0.	2.377874	-1.599635	1.141370
15.	6.	0.	2.825883	-0.317952	-1.215846
16.	6.	0.	0.007664	1.052297	-1.613327
17.	8.	0.	-0.055604	3.151656	0.526415
18.	1.	0.	-2.371697	1.709302	-0.463496
19.	1.	0.	-0.068498	-1.687771	-1.735509
20.	1.	0.	-1.848482	-3.199797	-0.936515
21.	1.	0.	-3.910278	-2.267516	0.095347
22.	1.	0.	-4.166961	0.195727	0.322287
23.	1.	0.	1.773125	2.104136	-0.982439
24.	1.	0.	-0.039007	1.832361	2.585532
25.	1.	0.	0.187519	-0.178645	4.015264
26.	1.	0.	1.222975	-1.565216	3.311085

27.	1.	0.	3.298239	0.523408	-1.732006
28.	1.	0.	3.583314	-0.932074	-0.730952
29.	1.	0.	2.307437	-0.937701	-1.957192
30.	1.	0.	-0.412076	2.022742	-1.892635
31.	1.	0.	0.504246	0.647046	-2.501049

S-OR-**2b**			Standard Orientation (Ångstroms)		
No.	Atom	Type	X	Y	Z
1.	6.	0.	-2.159868	0.869500	-0.635390
2.	6.	0.	-1.152803	0.006913	-1.094099
3.	6.	0.	-1.273549	-1.363651	-0.826467
4.	6.	0.	-2.366635	-1.860739	-0.115833
5.	6.	0.	-3.360408	-0.992179	0.336608
6.	6.	0.	-3.254304	0.374636	0.073540
7.	7.	0.	1.900819	-0.231741	-0.313258
8.	6.	0.	1.265258	0.921318	-0.946942
9.	6.	0.	1.758768	-0.580228	1.006227
10.	6.	0.	1.137417	0.449176	1.908185
11.	7.	0.	0.834005	1.699225	1.349480
12.	6.	0.	0.854896	2.023259	0.024003
13.	6.	0.	0.944254	0.165845	3.201476
14.	8.	0.	2.164762	-1.647942	1.453752
15.	6.	0.	2.609402	-1.162121	-1.185409
16.	6.	0.	0.040626	0.554417	-1.844209
17.	8.	0.	0.528637	3.128344	-0.386055
18.	1.	0.	-2.079090	1.934425	-0.835484
19.	1.	0.	-0.510303	-2.050726	-1.182151
20.	1.	0.	-2.441807	-2.926110	0.081215
21.	1.	0.	-4.213860	-1.378111	0.886329
22.	1.	0.	-4.027029	1.056899	0.416259
23.	1.	0.	2.008879	1.385641	-1.606750
24.	1.	0.	0.506266	2.438488	1.960042
25.	1.	0.	0.515331	0.885220	3.890812
26.	1.	0.	1.232386	-0.809578	3.568133
27.	1.	0.	3.291506	-1.753511	-0.576337
28.	1.	0.	1.928248	-1.849928	-1.700542
29.	1.	0.	3.170152	-0.597082	-1.936219
30.	1.	0.	-0.231721	1.473721	-2.370217
31.	1.	0.	0.378767	-0.162380	-2.599699

S-OR-**2c**			Standard Orientation (Ångstroms)		
No.	Atom	Type	X	Y	Z
1.	6.	0.	-1.301286	-0.660395	-1.940759
2.	6.	0.	-0.769702	0.548513	-1.474483
3.	6.	0.	-0.127596	1.394298	-2.390643
4.	6.	0.	-0.008226	1.034854	-3.732579

5.	6.	0.	-0.535374	-0.177186	-4.183565
6.	6.	0.	-1.185297	-1.022727	-3.284093
7.	7.	0.	1.018776	-0.418817	0.967222
8.	6.	0.	0.441444	0.914856	0.791998
9.	6.	0.	0.710677	-1.236844	2.022266
10.	6.	0.	0.040292	-0.581250	3.201058
11.	7.	0.	-0.043268	0.819926	3.193345
12.	6.	0.	0.185733	1.631444	2.116310
13.	6.	0.	-0.379545	-1.330456	4.227414
14.	8.	0.	1.010777	-2.426461	2.049717
15.	6.	0.	1.813974	-0.991191	-0.119682
16.	6.	0.	-0.891806	0.945221	-0.019546
17.	8.	0.	0.116205	2.848903	2.182550
18.	1.	0.	-1.810624	-1.323305	-1.245853
19.	1.	0.	0.273907	2.346554	-2.051443
20.	1.	0.	0.490430	1.703967	-4.427903
21.	1.	0.	-0.445146	-0.456613	-5.229021
22.	1.	0.	-1.604980	-1.964233	-3.626297
23.	1.	0.	1.177403	1.529039	0.264699
24.	1.	0.	-0.288125	1.301952	4.050166
25.	1.	0.	-0.843488	-0.896196	5.106603
26.	1.	0.	-0.232274	-2.400849	4.182894
27.	1.	0.	1.190467	-1.468717	-0.880349
28.	1.	0.	2.393910	-0.194315	-0.591887
29.	1.	0.	2.483491	-1.740139	0.303010
30.	1.	0.	-1.616753	0.298597	0.486997
31.	1.	0.	-1.265996	1.972570	0.052853

S-OR-**2d**			Standard Orientation (Ångstroms)		
No.	Atom	Type	X	Y	Z
1.	6.	0.	-1.742630	-0.581400	-1.587168
2.	6.	0.	-0.879181	0.509508	-1.426375
3.	6.	0.	-0.239664	1.029191	-2.561240
4.	6.	0.	-0.446986	0.465006	-3.819403
5.	6.	0.	-1.304106	-0.627849	-3.965369
6.	6.	0.	-1.954082	-1.147699	-2.845685
7.	7.	0.	1.056217	-0.519353	0.874037
8.	6.	0.	0.770270	0.877767	0.543827
9.	6.	0.	0.766974	-1.076574	2.091834
10.	6.	0.	0.483025	-0.114177	3.215187
11.	7.	0.	0.715231	1.247708	2.966693
12.	6.	0.	0.923634	1.815309	1.739747
13.	6.	0.	0.097237	-0.581497	4.408398
14.	8.	0.	0.792916	-2.287223	2.291002
15.	6.	0.	1.494874	-1.420280	-0.192375

16.	6.	0.	-0.645775	1.126022	-0.064558
17.	8.	0.	1.142053	3.008044	1.594482
18.	1.	0.	-2.253869	-0.990471	-0.719376
19.	1.	0.	0.418918	1.888825	-2.459502
20.	1.	0.	0.055321	0.882640	-4.687224
21.	1.	0.	-1.468144	-1.066117	-4.945274
22.	1.	0.	-2.628205	-1.992921	-2.949744
23.	1.	0.	1.518115	1.207172	-0.183579
24.	1.	0.	0.742759	1.891715	3.748322
25.	1.	0.	-0.092257	0.074910	5.251022
26.	1.	0.	-0.009336	-1.649858	4.537505
27.	1.	0.	0.654217	-1.828985	-0.759521
28.	1.	0.	2.144292	-0.869009	-0.876969
29.	1.	0.	2.044252	-2.245543	0.260371
30.	1.	0.	-1.394107	0.760213	0.647092
31.	1.	0.	-0.760962	2.213928	-0.127160

S-OR-**2e**			Standard Orientation (Ångstroms)		
No.	Atom	Type	X	Y	Z
1.	6.	0.	-2.061495	0.237678	-2.044102
2.	6.	0.	-1.620723	0.592090	-0.763835
3.	6.	0.	-1.920725	1.873886	-0.277558
4.	6.	0.	-2.641464	2.775793	-1.059353
5.	6.	0.	-3.076637	2.412362	-2.336083
6.	6.	0.	-2.785726	1.139654	-2.826411
7.	7.	0.	1.667536	-0.736106	0.009344
8.	6.	0.	0.591238	0.118984	0.508532
9.	6.	0.	2.324300	-1.680894	0.755301
10.	6.	0.	2.104786	-1.625462	2.242981
11.	7.	0.	1.349863	-0.555738	2.747664
12.	6.	0.	0.624149	0.339284	2.015175
13.	6.	0.	2.666155	-2.551080	3.029619
14.	8.	0.	3.088806	-2.506983	0.266806
15.	6.	0.	1.916098	-0.684193	-1.427299
16.	6.	0.	-0.839860	-0.387807	0.087637
17.	8.	0.	-0.015101	1.242699	2.536560
18.	1.	0.	-1.840568	-0.754117	-2.431994
19.	1.	0.	-1.583558	2.155283	0.716801
20.	1.	0.	-2.866830	3.764294	-0.669243
21.	1.	0.	-3.638619	3.116259	-2.942938
22.	1.	0.	-3.119951	0.846699	-3.817622
23.	1.	0.	0.738273	1.112909	0.072570
24.	1.	0.	1.308172	-0.417930	3.750795
25.	1.	0.	2.547809	-2.539406	4.107890
26.	1.	0.	3.265678	-3.326394	2.573006

27.	1.	0.	1.054482	-1.048926	-1.998262
28.	1.	0.	2.115964	0.349716	-1.728441
29.	1.	0.	2.777260	-1.312581	-1.646179
30.	1.	0.	-0.718794	-1.334620	-0.446646
31.	1.	0.	-1.411518	-0.614352	0.994285

Figure S1. Colonies of *Streptomyces* sp. SY1965

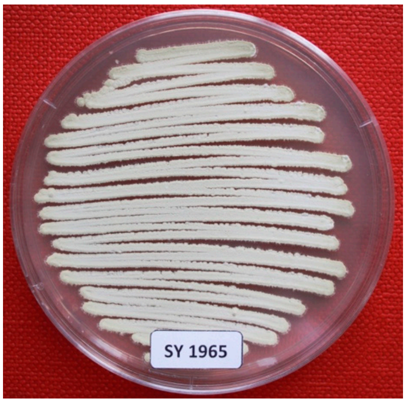

Figure S2. 16S rDNA sequence of *Streptomyces* sp. SY1965

CTGCAGTCGAACGATGAAGCCGCTTCGGTGGTGGATTAGTGGCGAACGGGTGAGTAACACGTG
GGCAATCTGCCCTTCACTCTGGGACAAGCCCTGGAAACGGGGTCTAATACCGGATAACACTCTG
TCCCGCATGGGACGGGGTTAAAAGCTCCGGCGGTGAAGGATGAGCCCGCGGCCTATCAGCTTGT
TGGTGGGGTAATGGCCTACCAAGGCGACGACGGGTAGCCGGCCTGAGAGGGCGACCGGCCACA
CTGGGACTGAGACACGGCCCAGACTCCTACGGGAGGCAGCAGTGGGGAATATTGCACAATGGG
CGAAAGCCTGATGCAGCGACGCCGCGTGAGGGATGACGGCCTTCGGGTTGTAAACCTCTTTCAG
CAGGGAAGAAGCGAAAGTGACGGTACCTGCAGAAGAAGCGCCGGCTAACTACGTGCCAGCAG
CCGCGGTAATACGTAGGGCGCAAGCGTTGTCCGGAATTATTGGGCGTAAAGAGCTCGTAGGCGG
CTTGTCACGTCGGATGTGAAAGCCCGGGGCTTAACCCCGGGTCTGCATTCGATACGGGCTAGCTA
GAGTGTGGTAGGGGAGATCGGAATTCCTGGTGTAGCGGTGAAATGCGCAGATATCAGGAGGAAC
ACCGGTGGCGAAGGCGGATCTCTGGGCCATTACTGACGCTGAGGAGCGAAAGCGTGGGGAGCG
AACAGGATTAGATACCCTGGTAGTCCACGCCGTAAACGTTGGGAACTAGGTGTTGGCGACATTC
CACGTCGTCGGTGCCGCAGCTAACGCATTAAGTTCCCCGCCTGGGGAGTACGGCCGCAAGGCTA
AAACTCAAAGGAATTGACGGGGGCCCGCACAAGCAGCGGAGCATGTGGCTTAATTCGACGCAA
CGCGAAGAACCTTACCAAGGCTTGACATATACCGGAAAGCATCAGAGATGGTGCCCCCCTTGTG
GTCGGTATACAGGTGGTGCATGGCTGTCGTCAGCTCGTGTCGTGAGATGTTGGGTTAAGTCCCGC
AACGAGCGCAACCCTTGTTCTGTGTTGCCAGCATGCCCTTCGGGGTGATGGGGACTCACAGGAG
ACTGCCGGGGTCAACTCGGAGGAAGGTGGGGACGACGTCAAGTCATCATGCCCCTTATGTCTTG
GGCTGCACACGTGCTACAATGGCCGGTACAATGAGCTGCGATGCCGCGAGGCGGAGCGAATCTC
AAAAAGCCGGTCTCAGTTCGGATTGGGGTCTGCAACTCGACCCCATGAAGTCGGAGTTGCTAGT
AATCGCAGATCAGCATTGCTGCGGTGAATACGTTCCCGGGCCTTGTACACACCGCCCGTCACGTC
ACGAAAGTCGGTAACACCCGAAGCCGGTGGCCCAACCCCTTGTGGGAAGGAGCTGTCAAAGGT
GGACTGCGATTGG (1416 bp)

Figure S3. HPLC separation chromatogram of streptodiketopiperazines A (**2**) and (**3**) on the chiral HPLC column (CHIRALCEL® OJ-RH, 150 × 4.6 mm, 5 µm; mobile phase: MeCN/H_2O, 11/89; flow rate: 0.4 mL/min; UV detection: 210 nm)

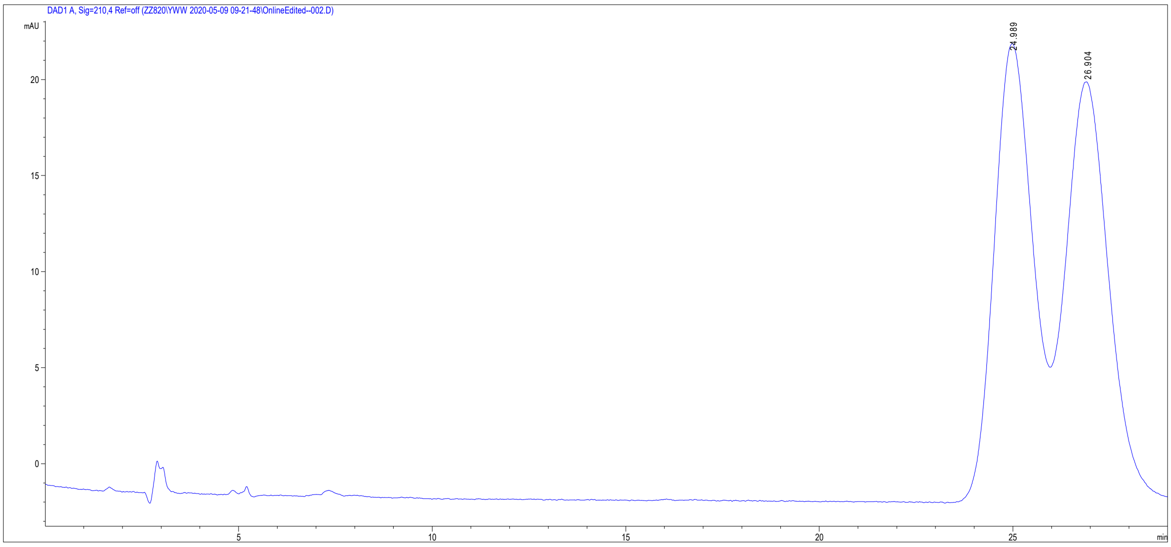

Figure S4. HPLC separation chromatogram of (*S*)-1-(3-ethylphenyl)-1,2-ethanediol (**4**) and (*R*-)1-(3-ethylphenyl)-1,2-ethanediol (**5**) on the chiral HPLC column (CHIRALCEL® OJ-RH,150 × 4.6 mm, 5 µm; mobile phase: MeCN/H_2O, 15/85; flow rate: 0.5 mL/min; UV detection: 210 nm)

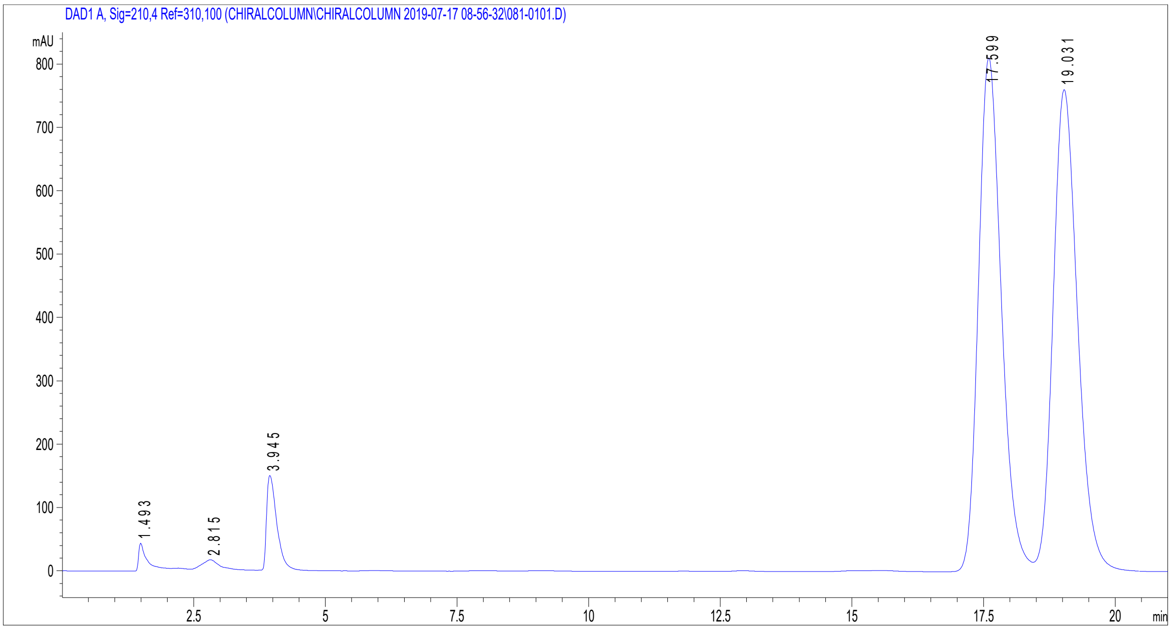

Figure S5. ^{1}H NMR spectrum of streptothiazomycin A (**1**)

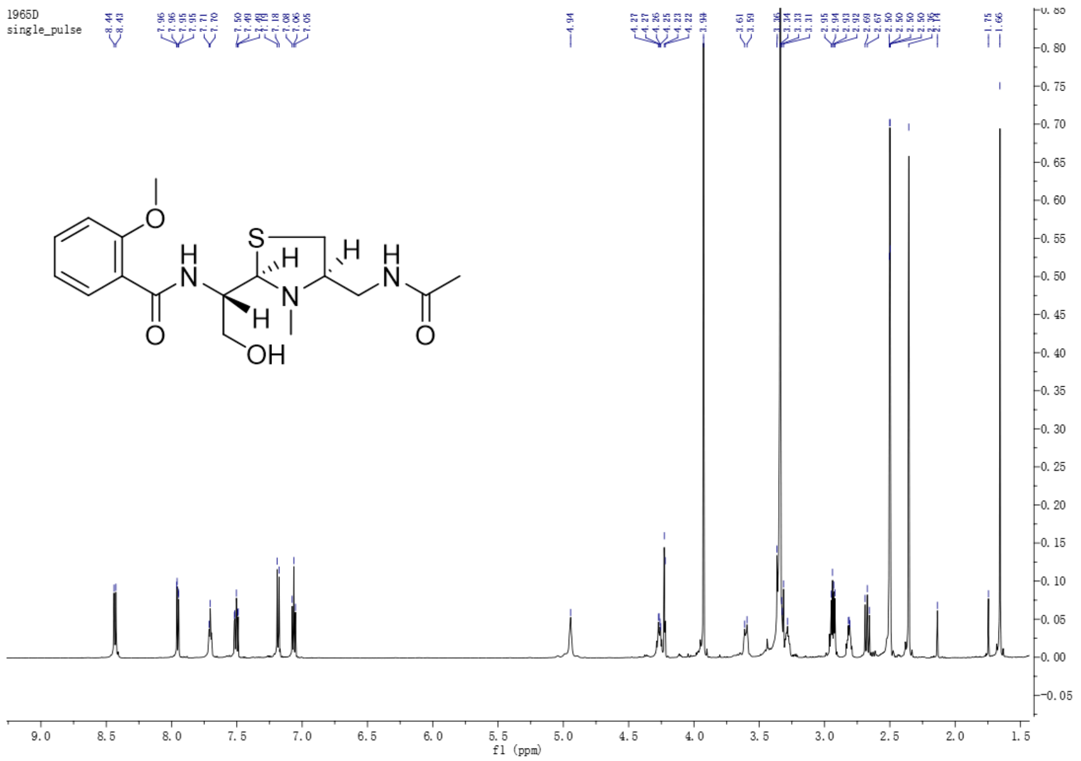

Figure S6. ^{1}H NMR spectrum of streptothiazomycin A (**1**)

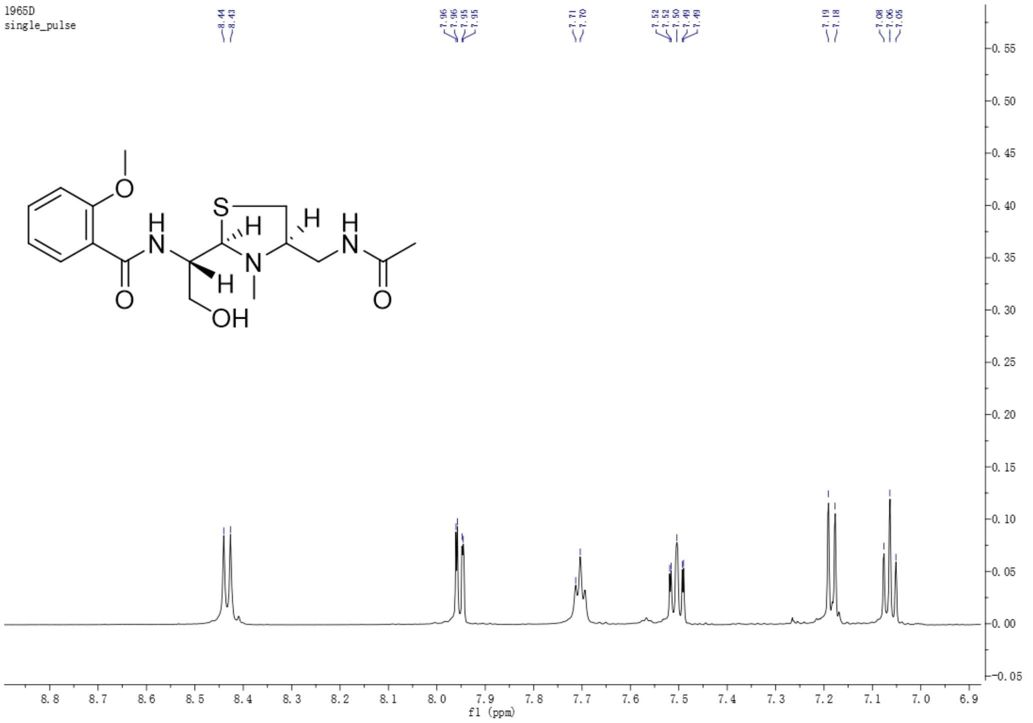

Figure S7. ^{1}H NMR spectrum of streptothiazomycin A (**1**)

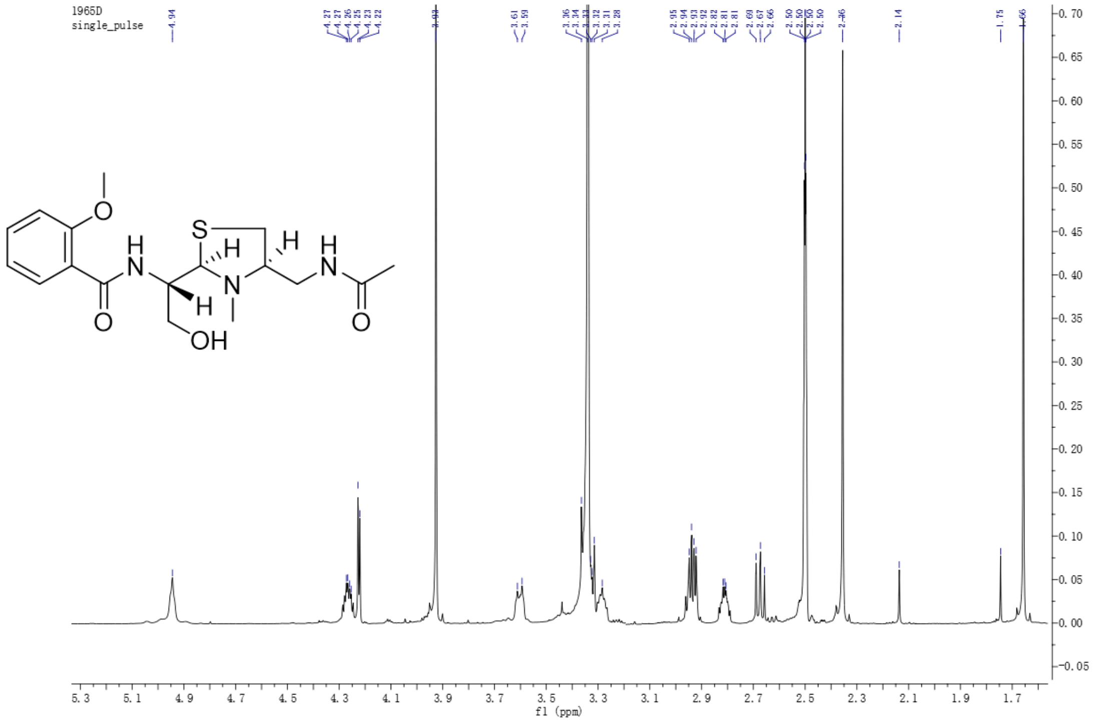

Figure S8. ^{13}C NMR spectrum of streptothiazomycin A (**1**)

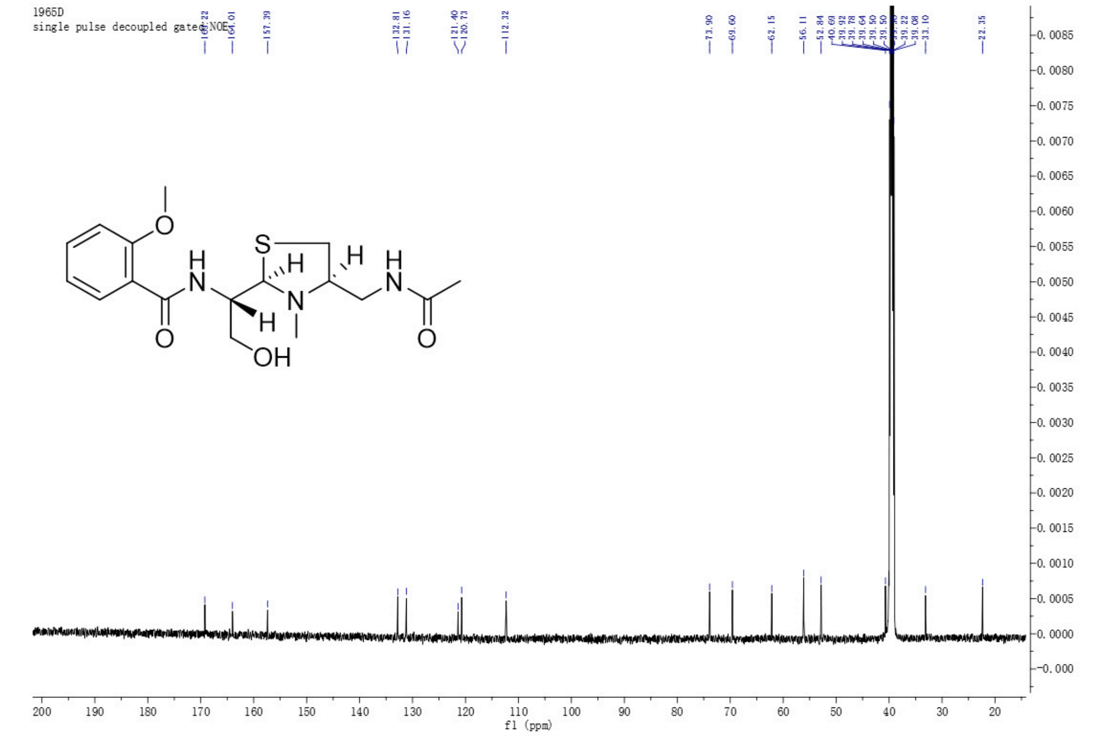

Figure S9. ^{13}C NMR spectrum of streptothiazomycin A (**1**)

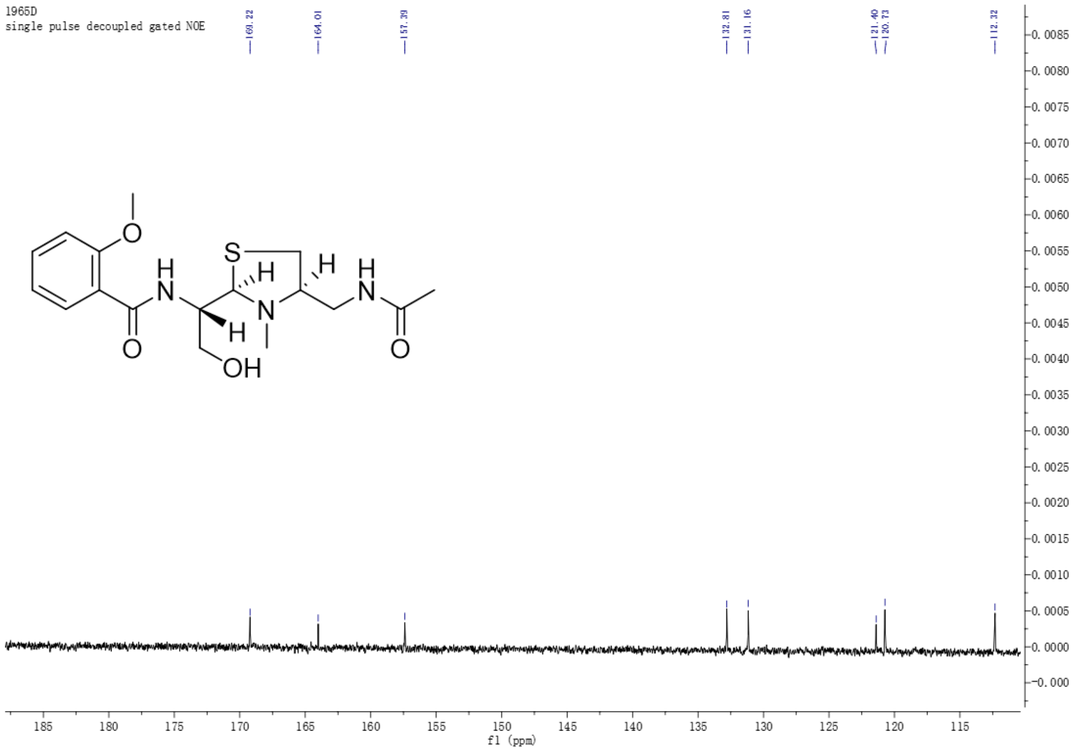

Figure S10. ^{13}C NMR spectrum of streptothiazomycin A (**1**)

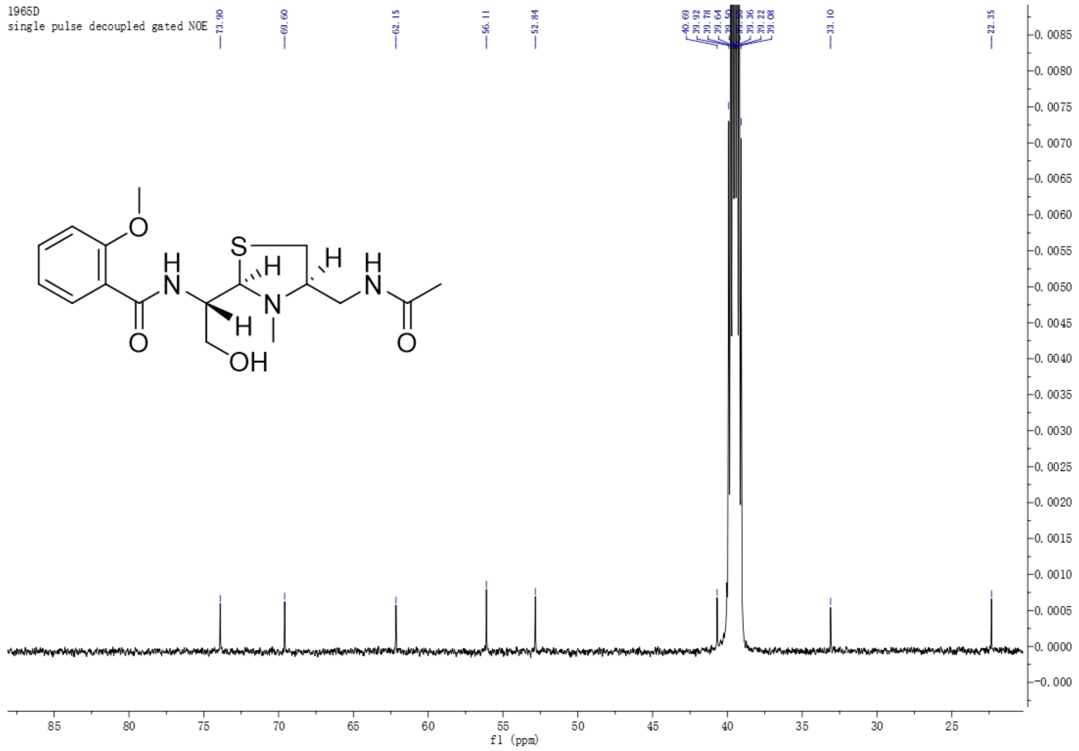

Figure S11. DEPT NMR spectrum of streptothiazomycin A (**1**)

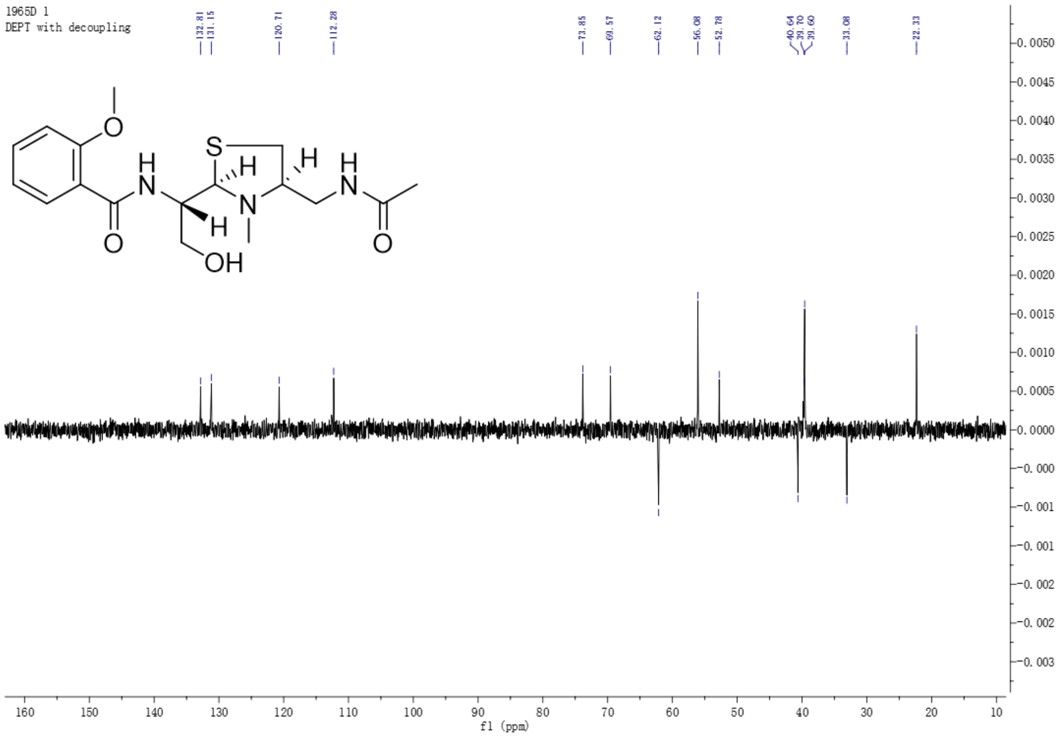

Figure S12. HMQC spectrum of streptothiazomycin A (**1**)

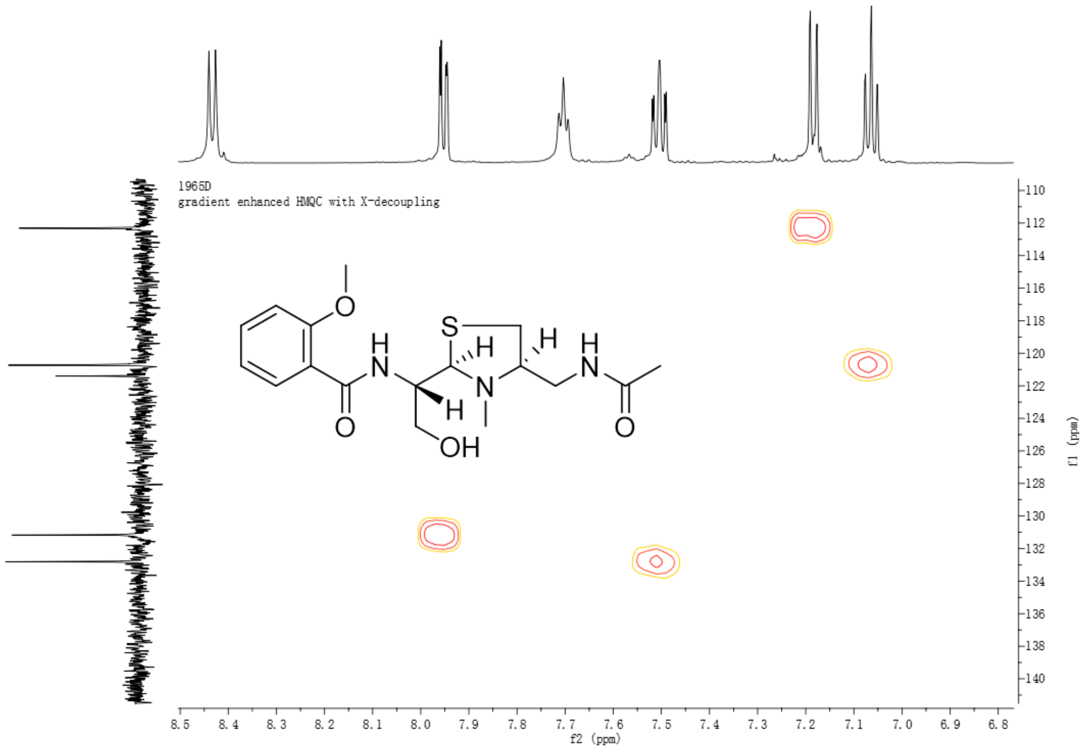

Figure S13. HMQC spectrum of streptothiazomycin A (**1**)

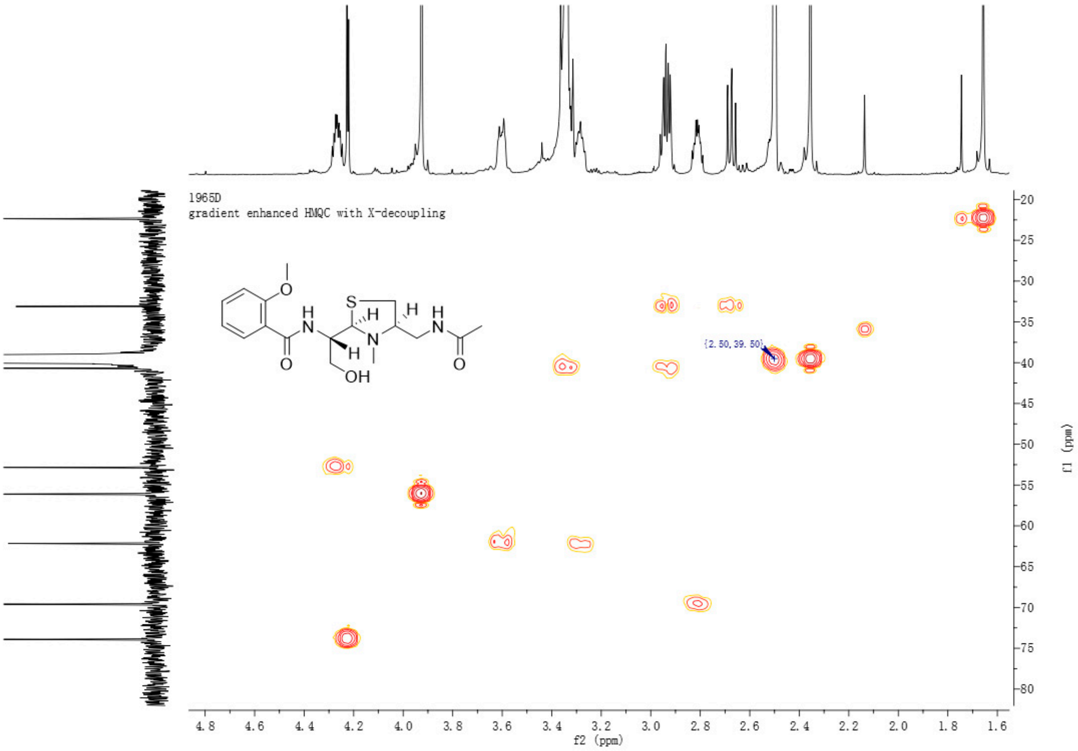

Figure S14. COSY spectrum of streptothiazomycin A (**1**)

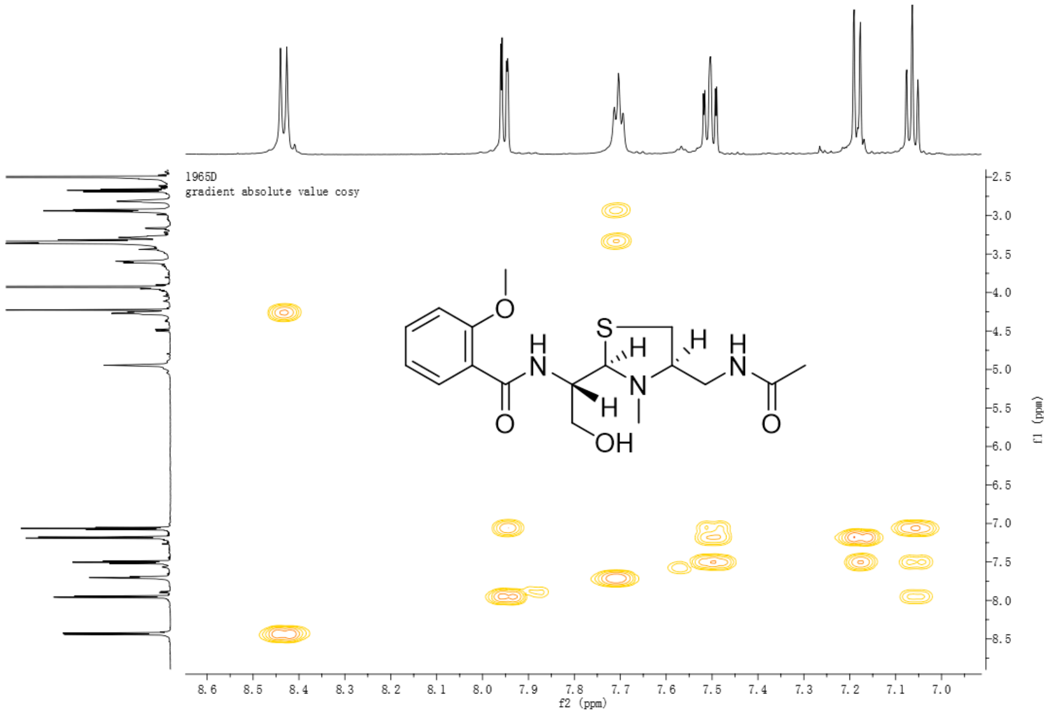

Figure S15. COSY spectrum of streptothiazomycin A (**1**)

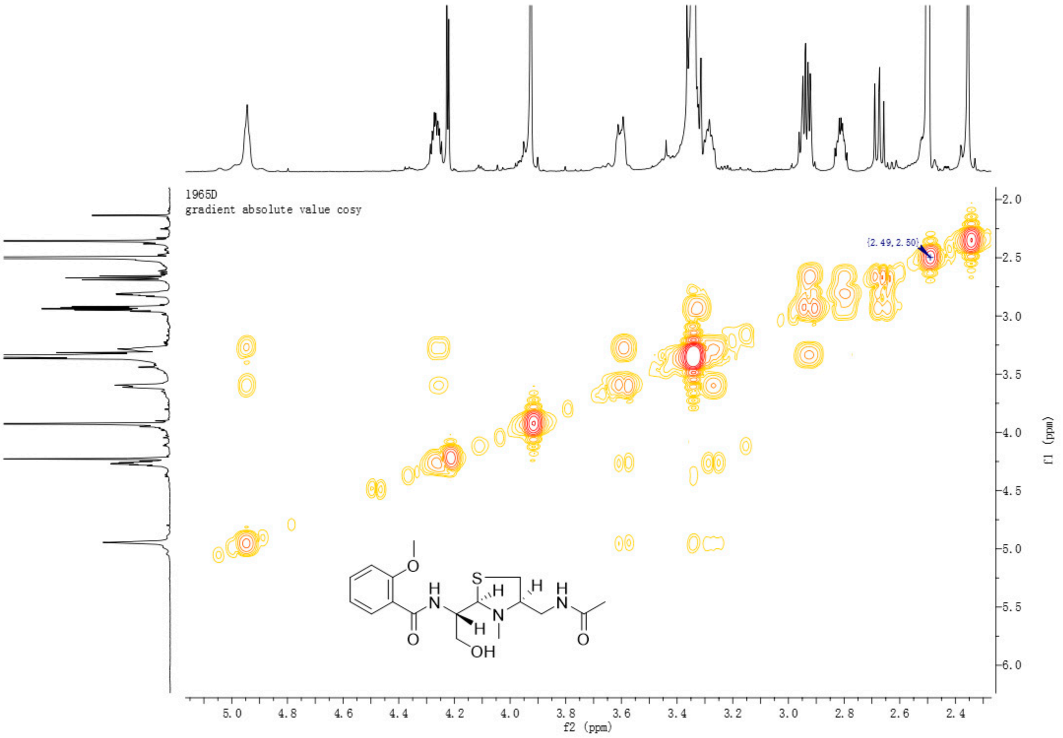

Figure S16. HMBC spectrum of streptothiazomycin A (**1**)

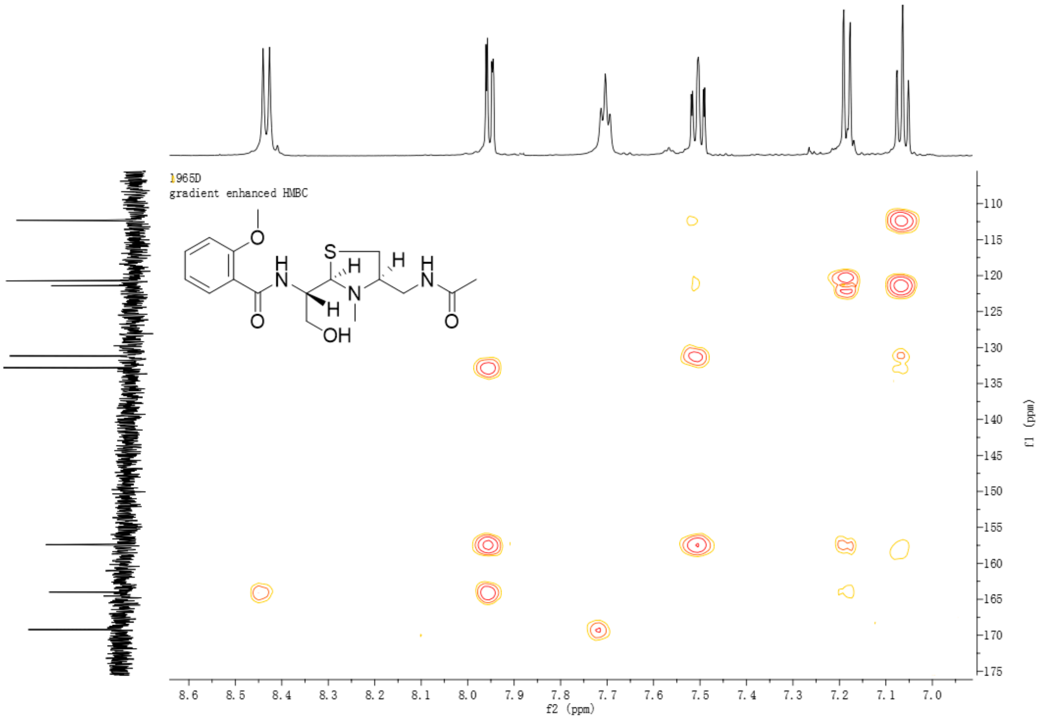

Figure S17. HMBC spectrum of streptothiazomycin A (**1**)

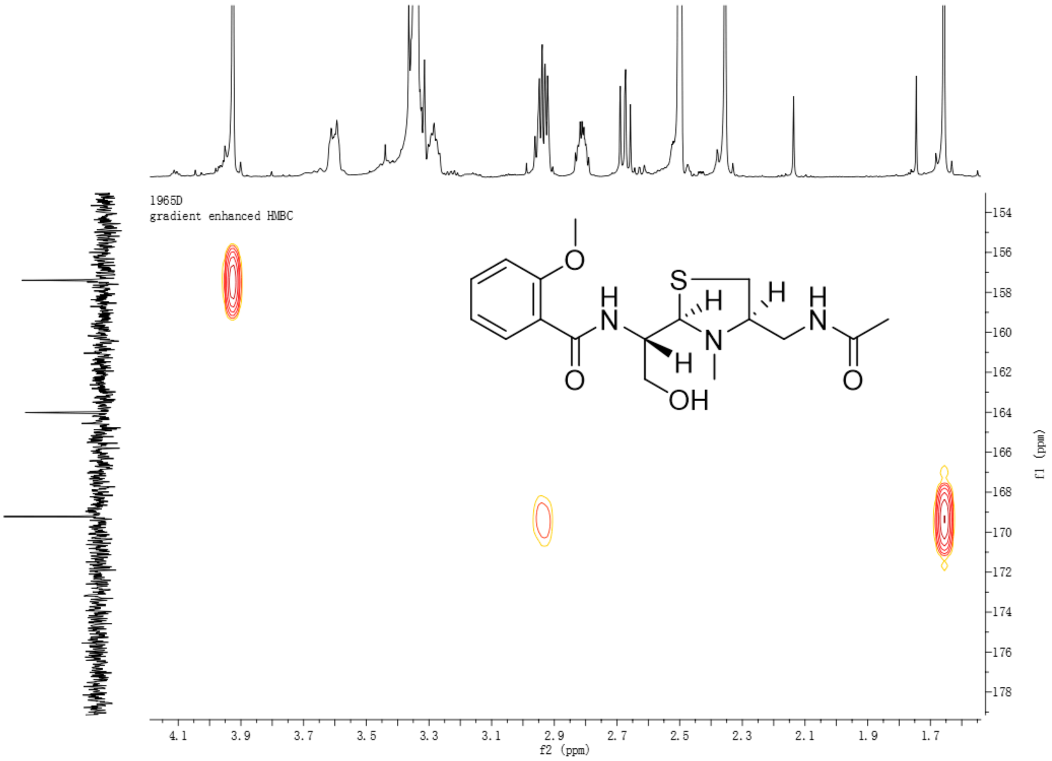

Figure S18. HMBC spectrum of streptothiazomycin A (**1**)

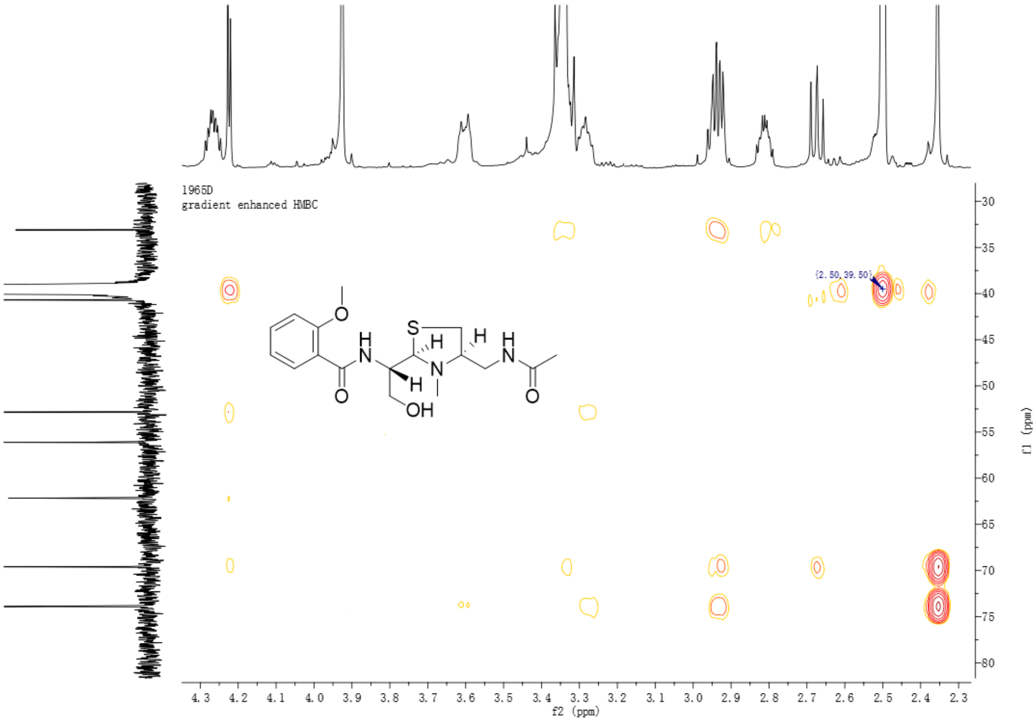

Figure S19. NOESY spectrum of streptothiazomycin A (**1**)

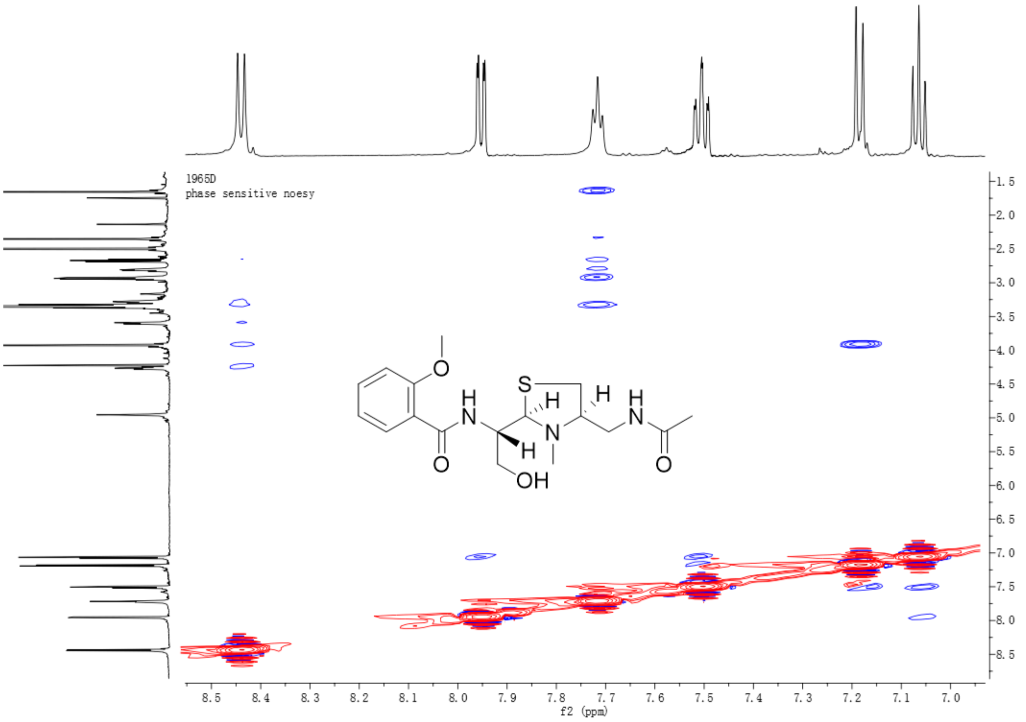

Figure S20. NOESY spectrum of streptothiazomycin A (**1**)

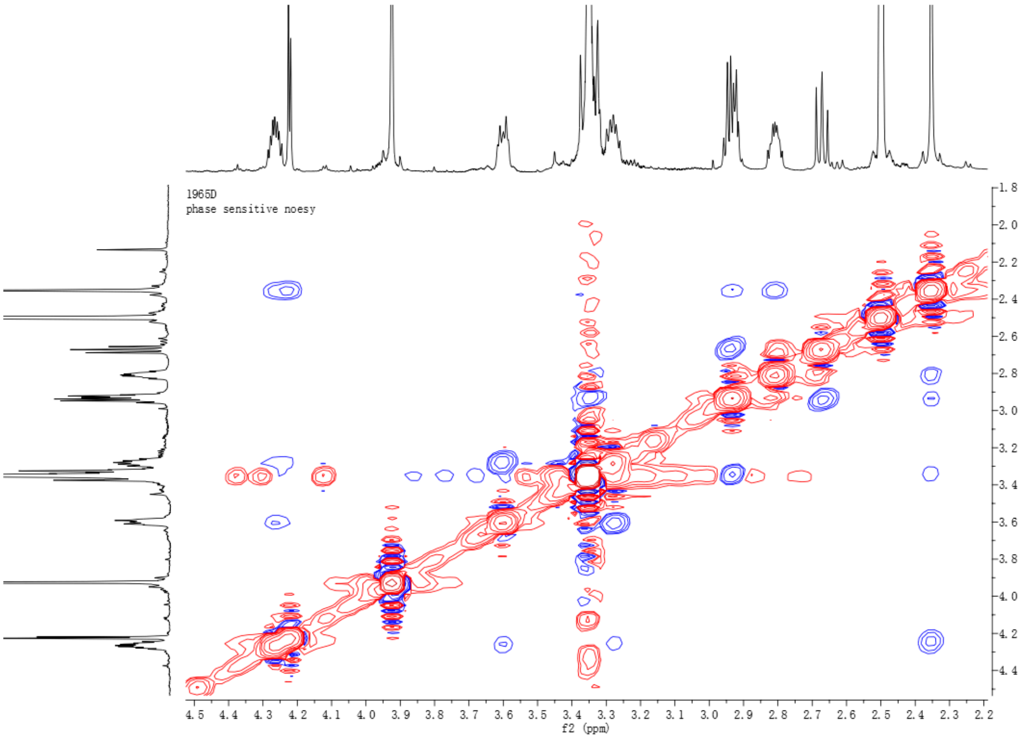

Figure S21. HRESIMS spectrum of streptothiazomycin A (**1**)

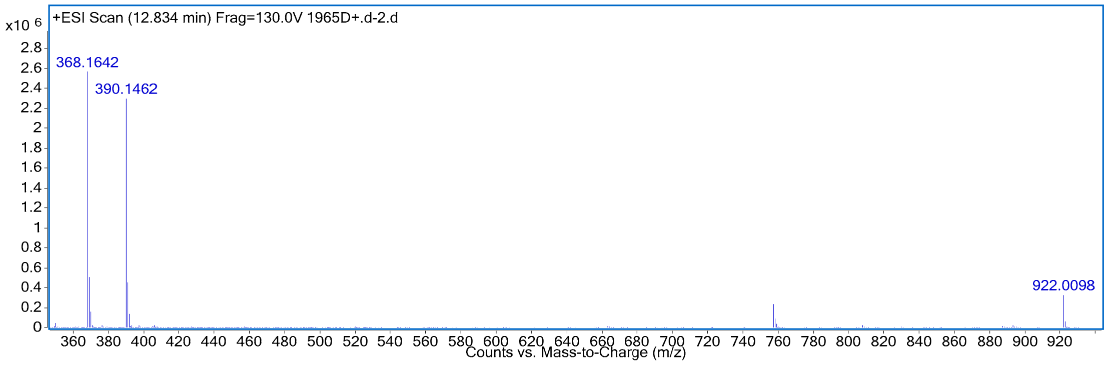

[M+H]+: 368.1642 (calcd for $C_{17}H_{26}N_3O_4S$, 368.1644); [M+Na]+: 390.1462 (calcd for $C_{17}H_{26}N_3O_4S$, 390.1463).

Figure S22. ^{1}H NMR spectrum of streptodiketopiperazines A (**2**) and B (**3**)

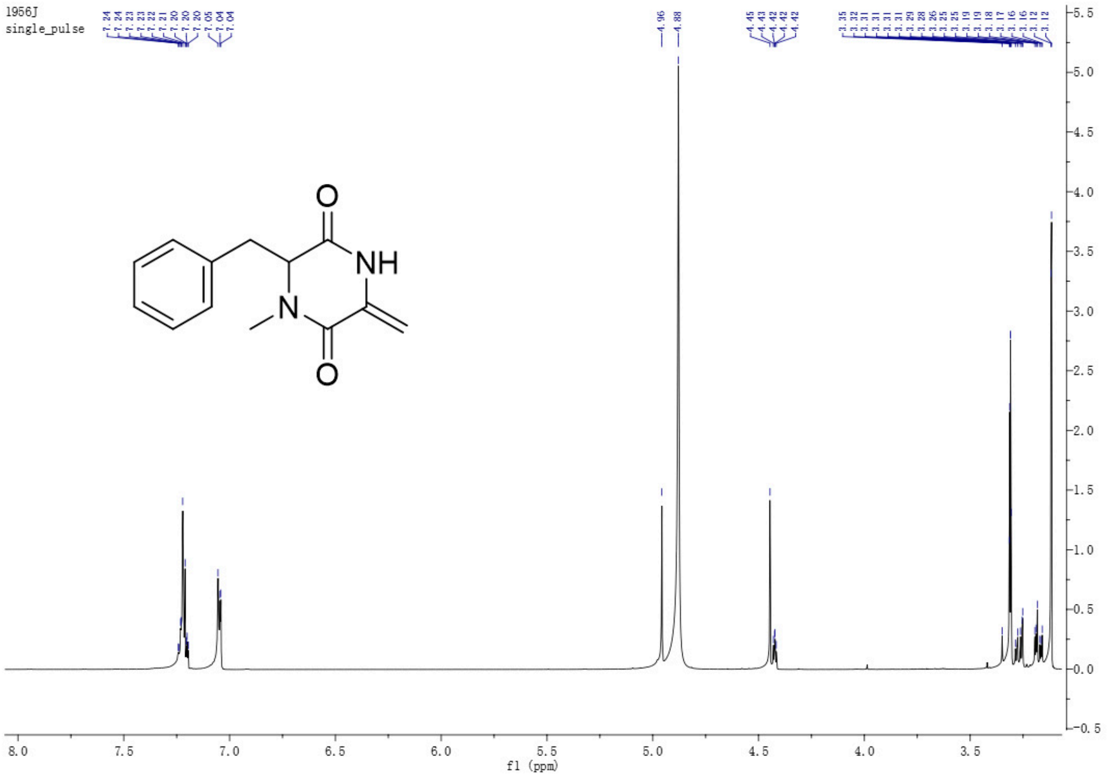

Figure S23. ^{13}C NMR spectrum of streptodiketopiperazines A (**2**) and B (**3**)

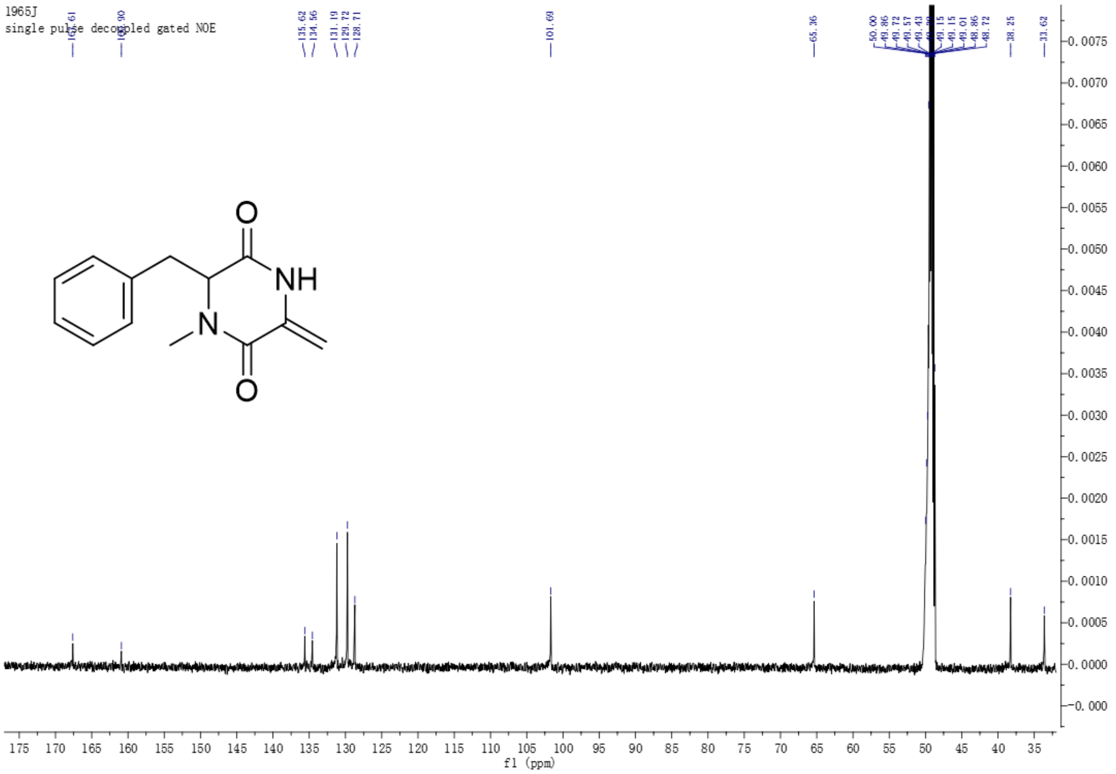

Figure S24. ^{13}C NMR spectrum of streptodiketopiperazines A (**2**) and B (**3**)

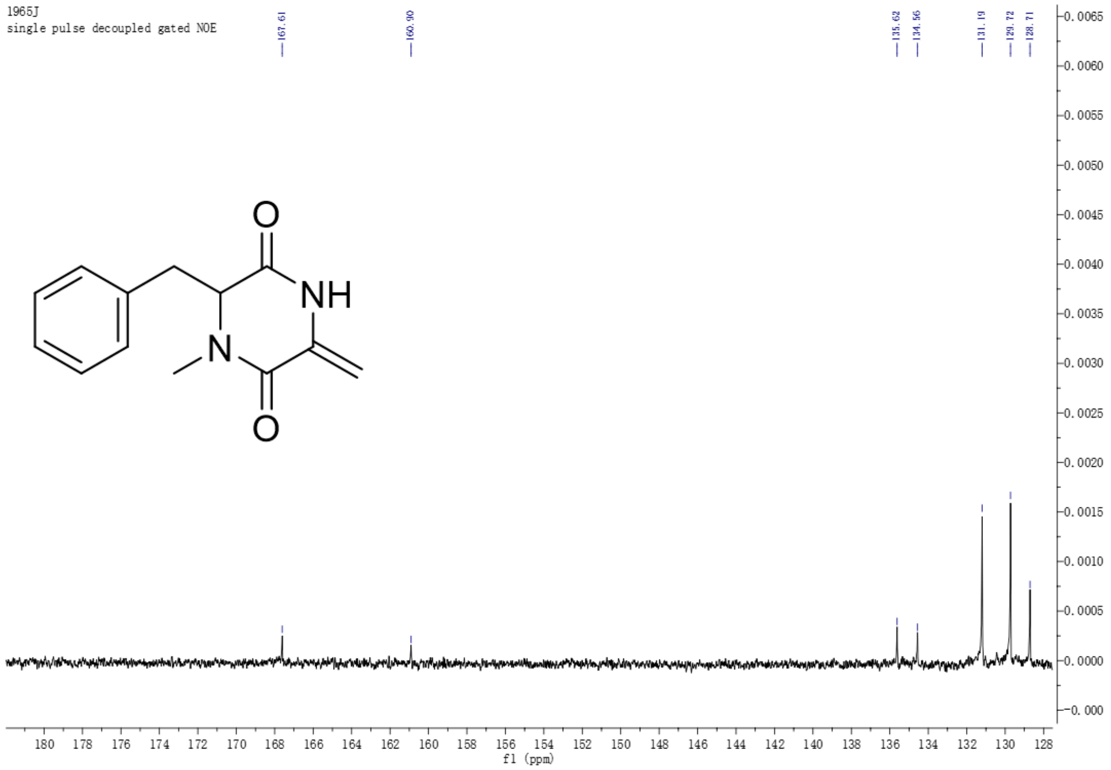

Figure S25. ^{13}C NMR spectrum of streptodiketopiperazines A (**2**) and B (**3**)

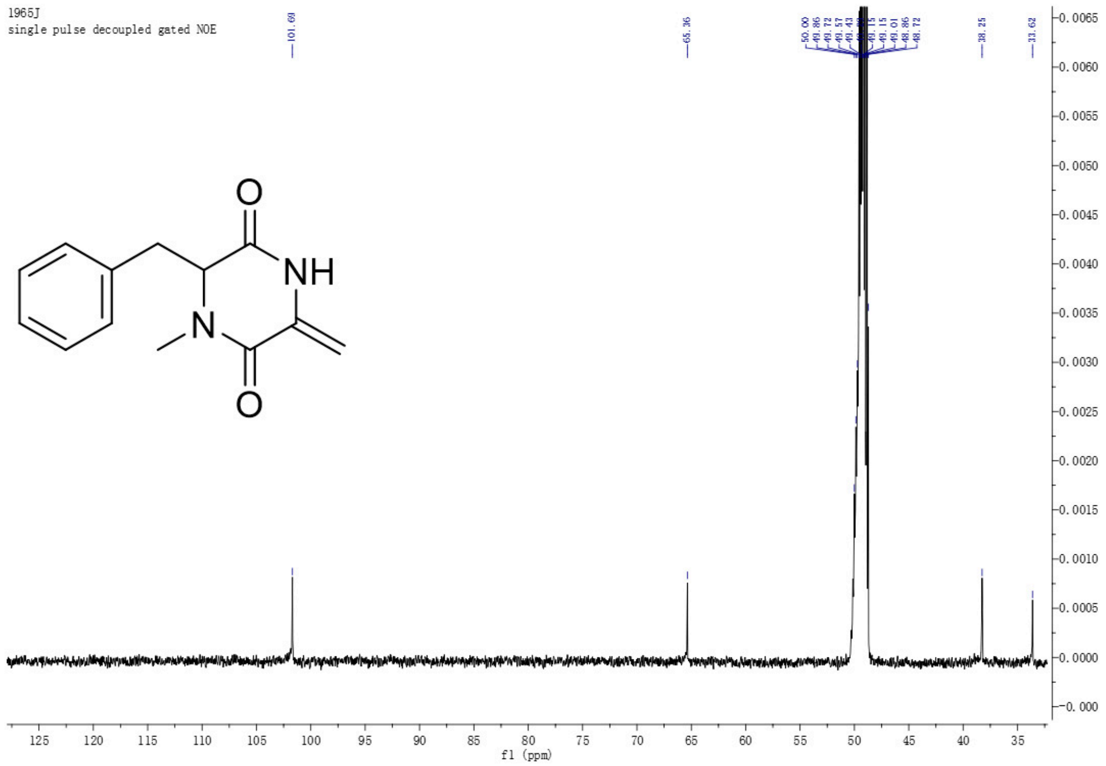

Figure S26. HMQC spectrum of streptodiketopiperazines A (**2**) and B (**3**)

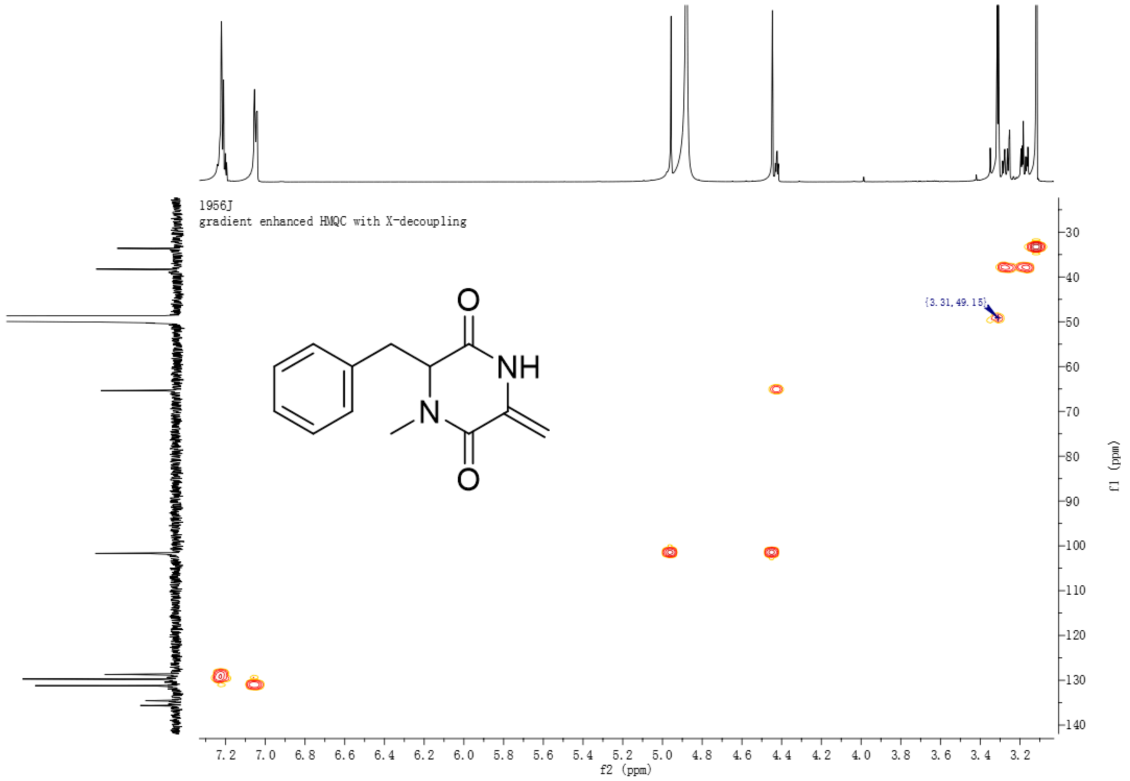

Figure S27. COSY spectrum of streptodiketopiperazines A (**2**) and B (**3**)

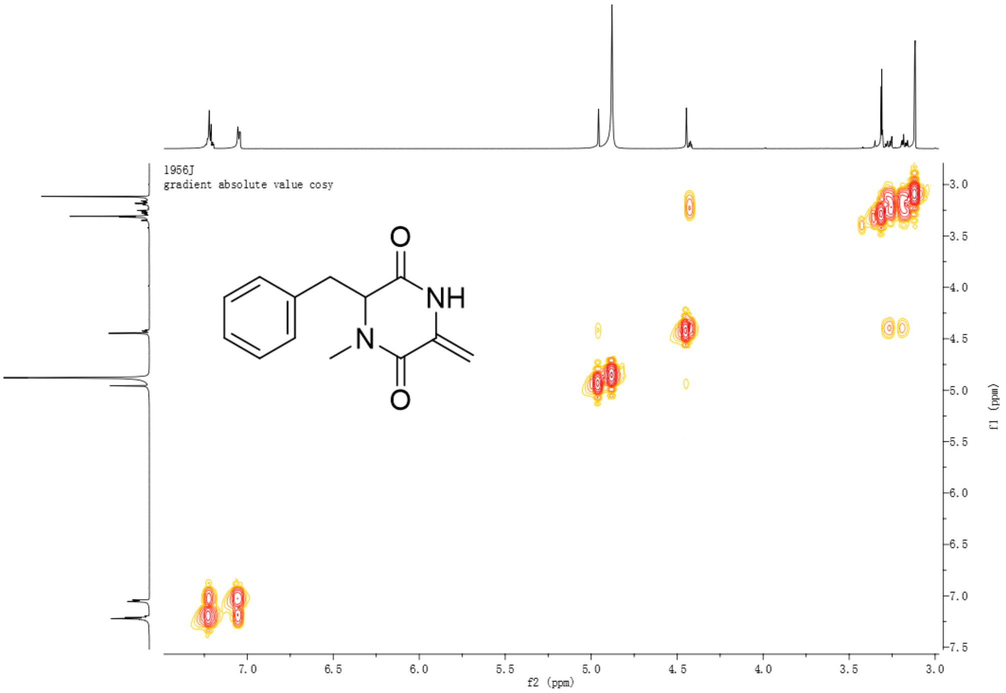

Figure S28. HMBC spectrum of streptodiketopiperazines A (**2**) and B (**3**)

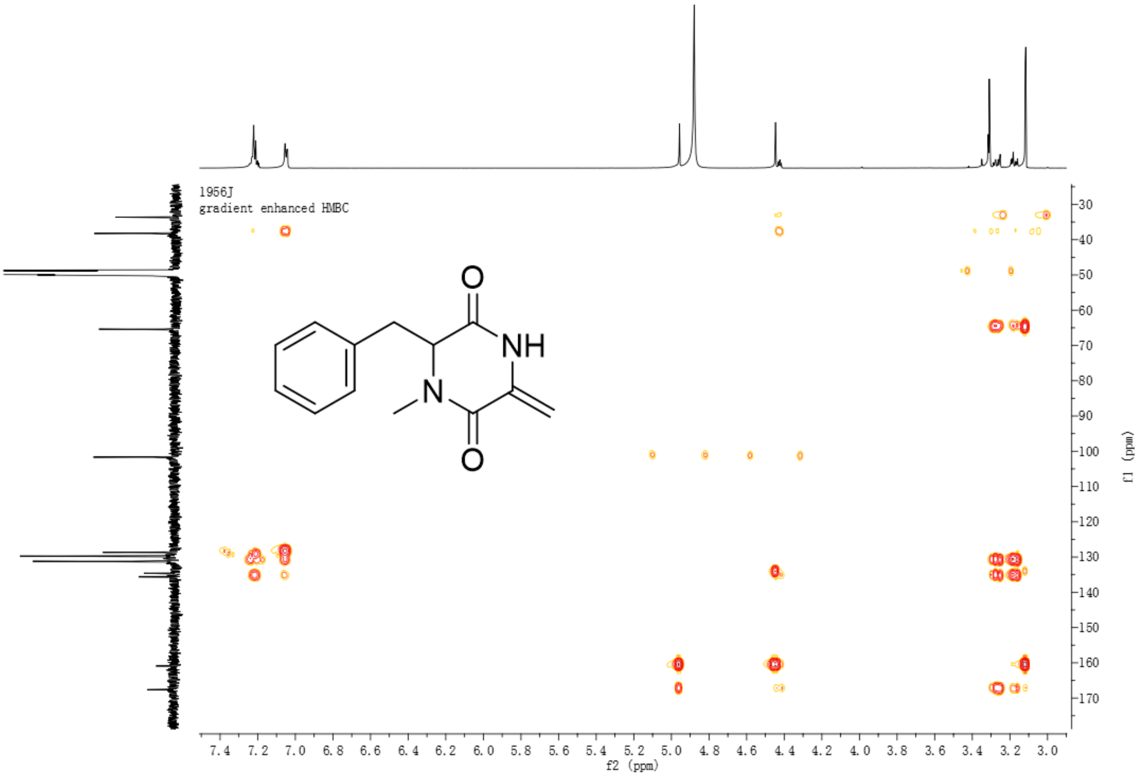

Figure S29. HMBC spectrum of streptodiketopiperazines A (**2**) and B (**3**)

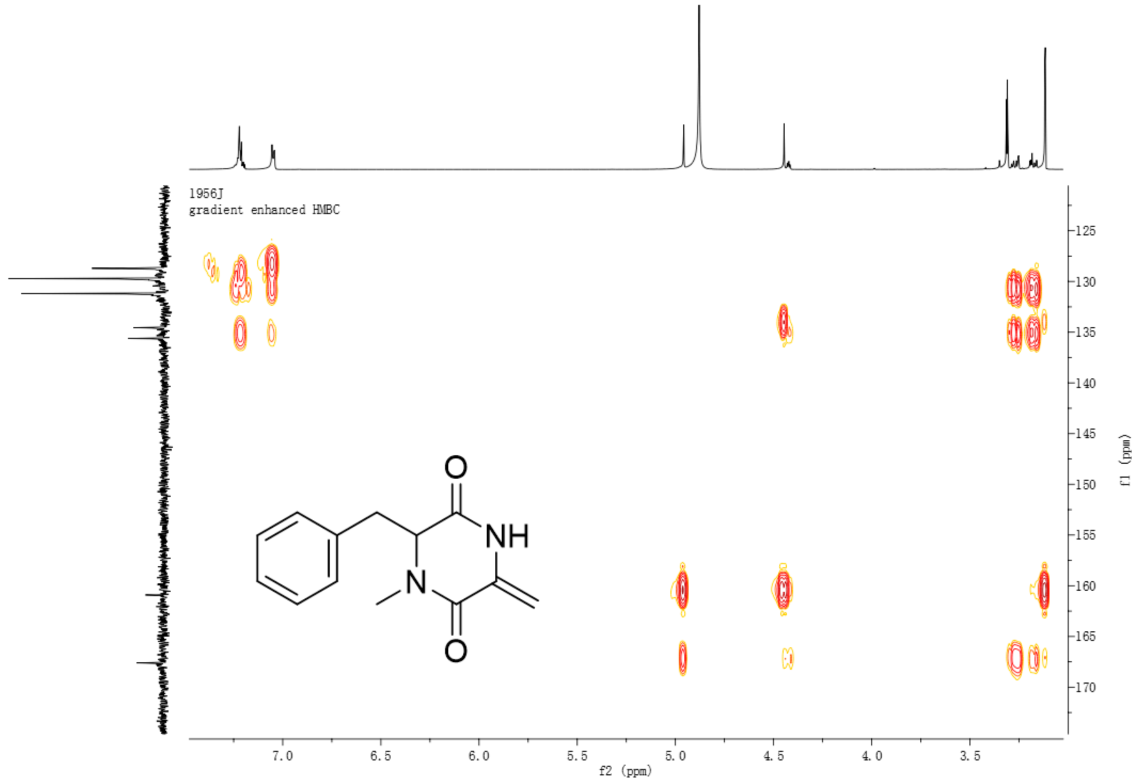

Figure S30. HMBC spectrum of streptodiketopiperazines A (**2**) and B (**3**)

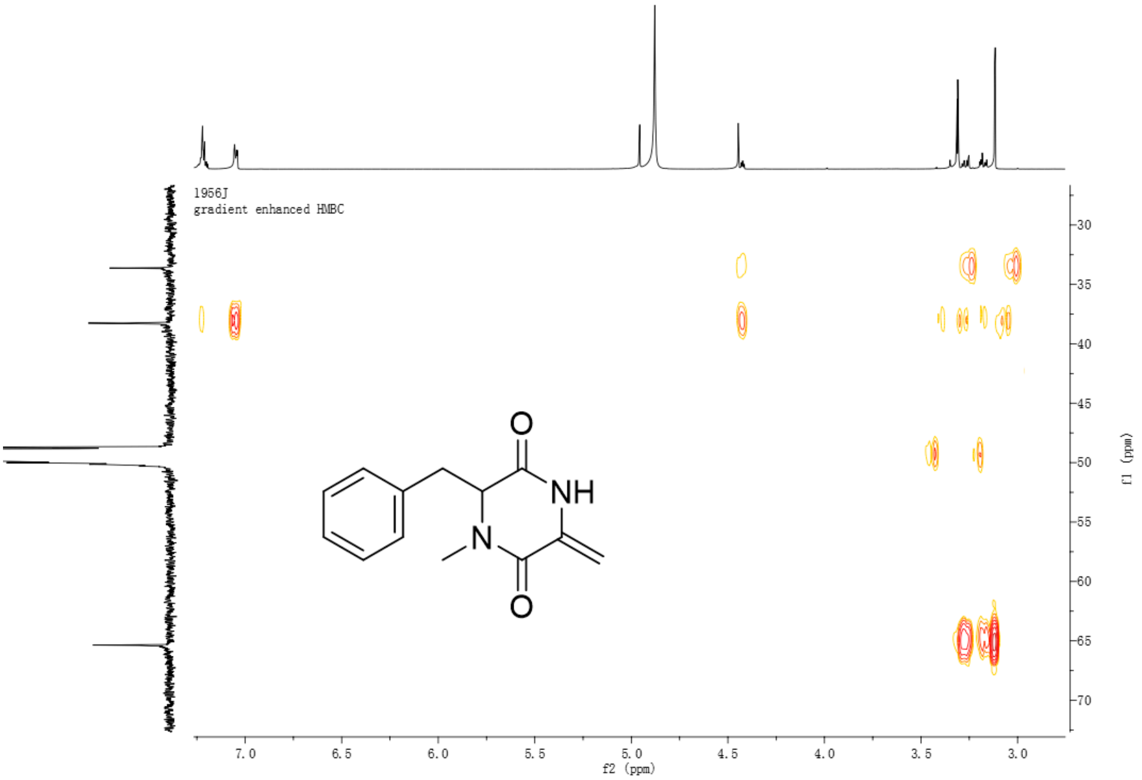

Figure S31. HRESIMS spectrum of streptodiketopiperazines A (**2**) and B (**3**)

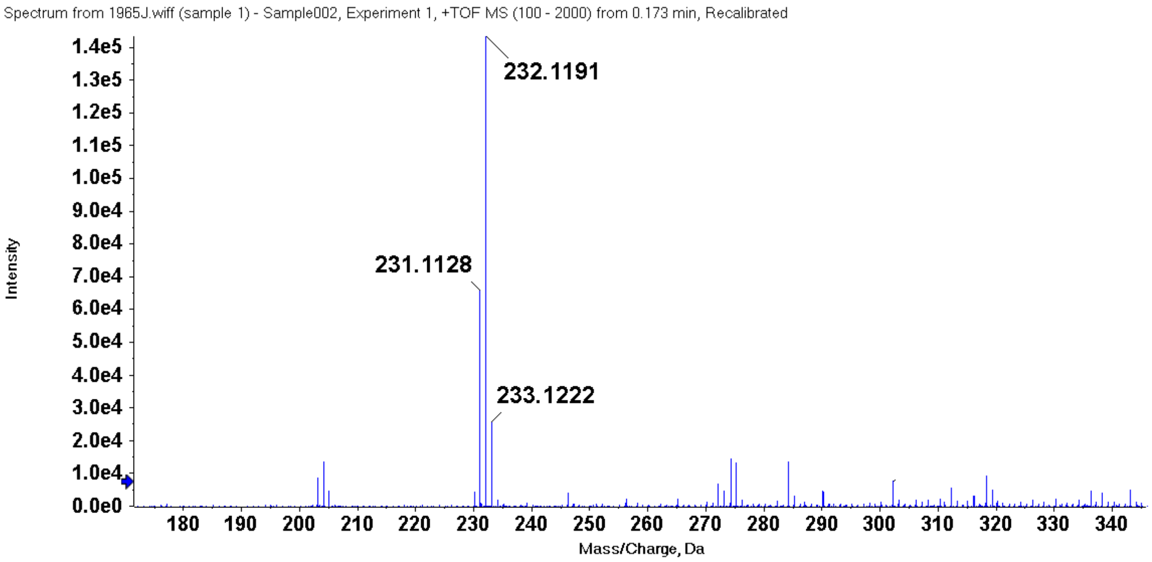

$[M+H]^+$: 231.1128 (calcd for $C_{13}H_{15}N_2O_2$, 231.1134).

Figure S32. ^{1}H NMR spectrum of (*S*)-1-(3-ethylphenyl)-1,2-ethanediol (**4**)

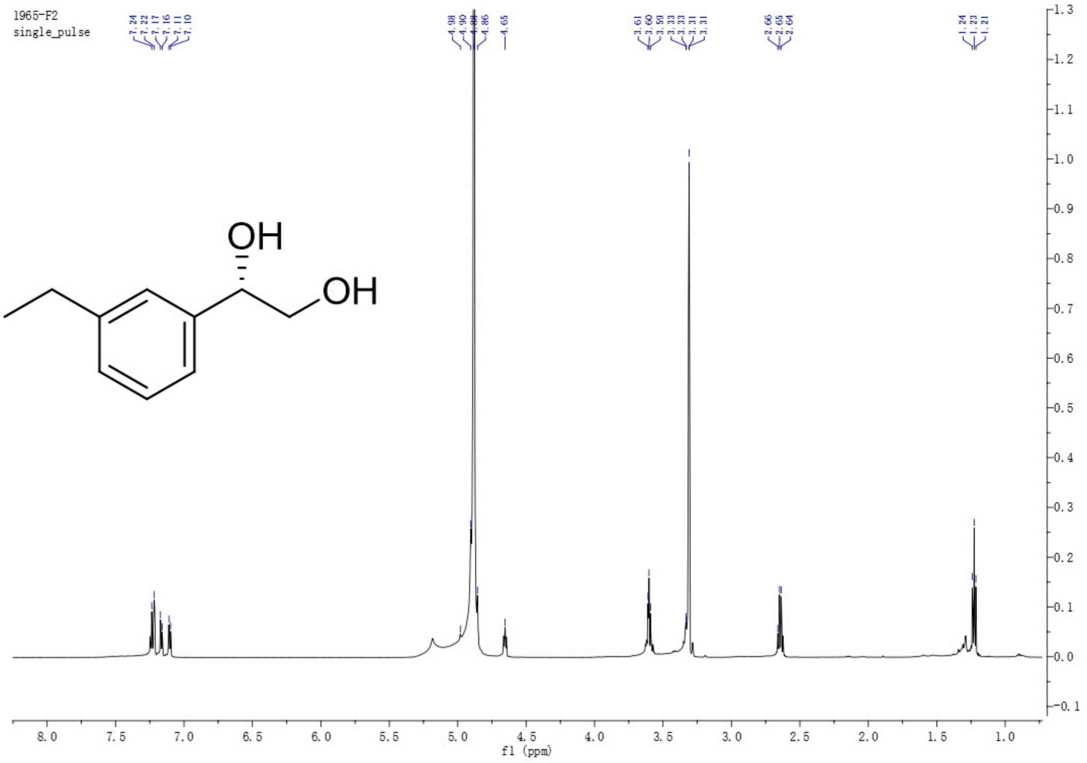

Figure S33. ^{1}H NMR spectrum of (*S*)-1-(3-ethylphenyl)-1,2-ethanediol (**4**)

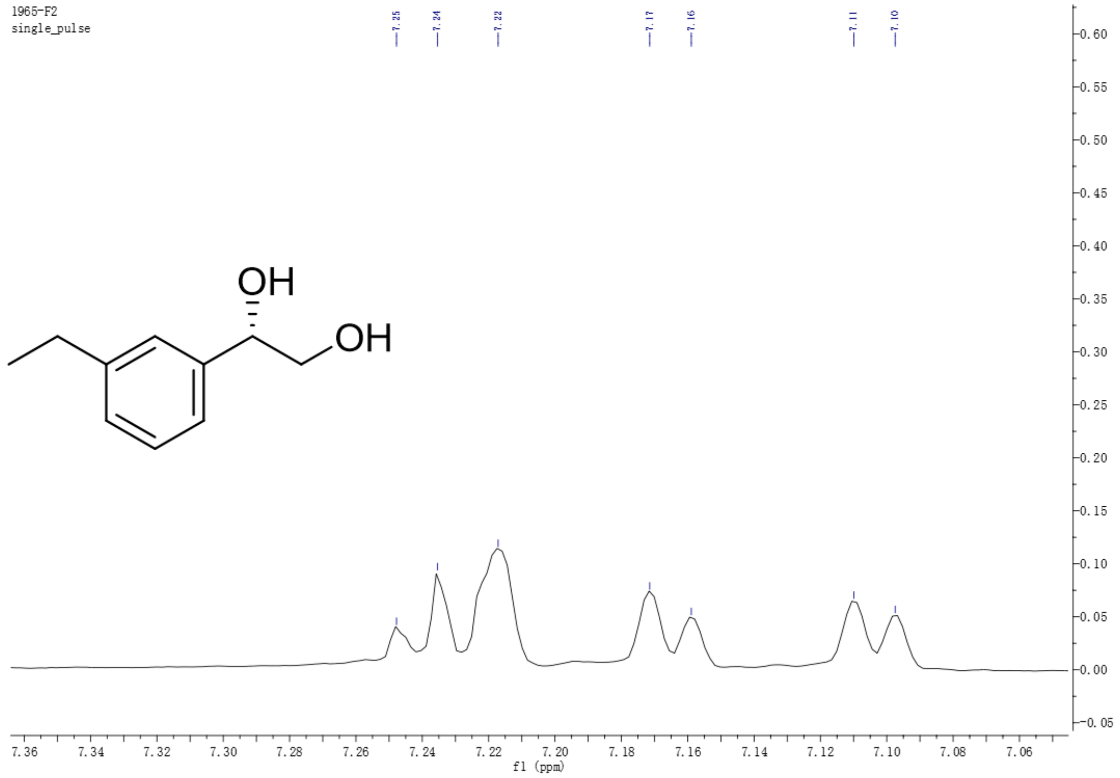

Figure S34. ^{1}H NMR spectrum of (*S*)-1-(3-ethylphenyl)-1,2-ethanediol (**4**)

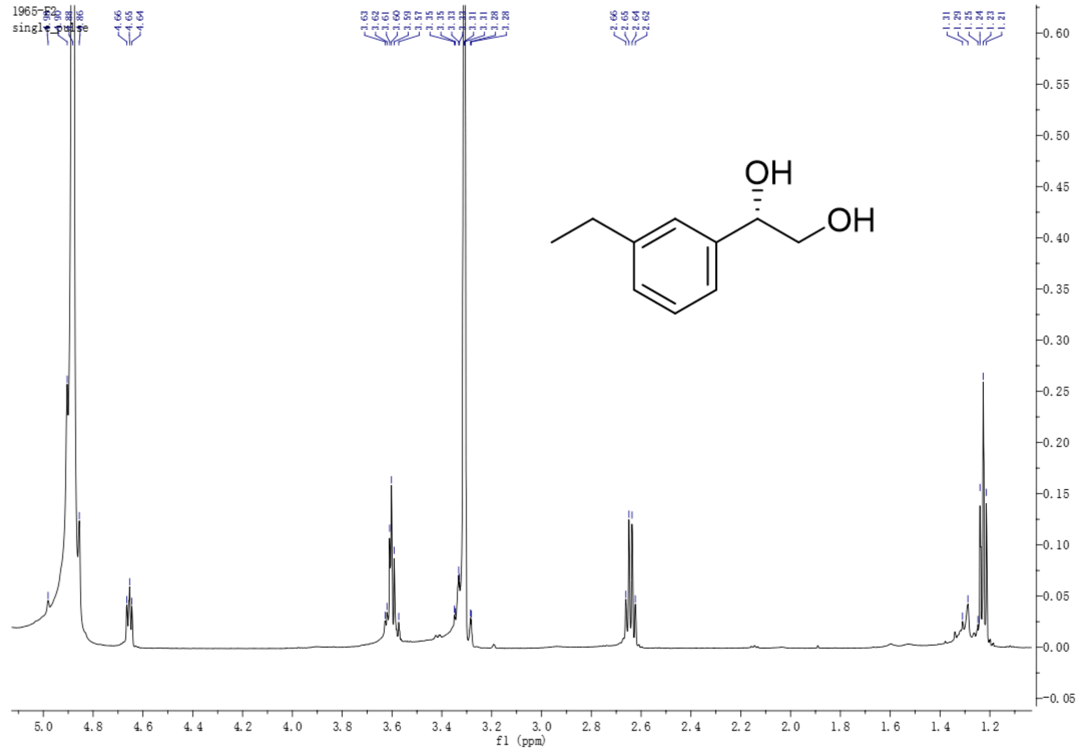

Figure S35. ^{13}C NMR spectrum of (*S*)-1-(3-ethylphenyl)-1,2-ethanediol (**4**)

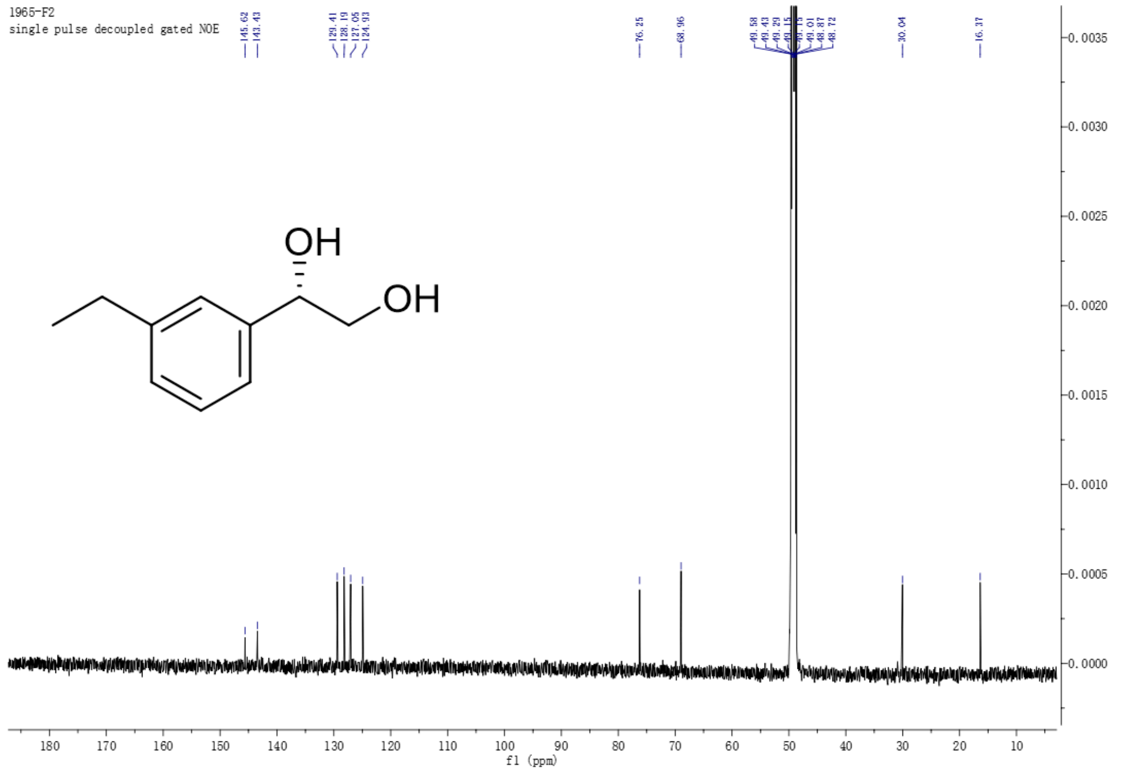

Figure S36. ^{13}C NMR spectrum of (*S*)-1-(3-ethylphenyl)-1,2-ethanediol (**4**)

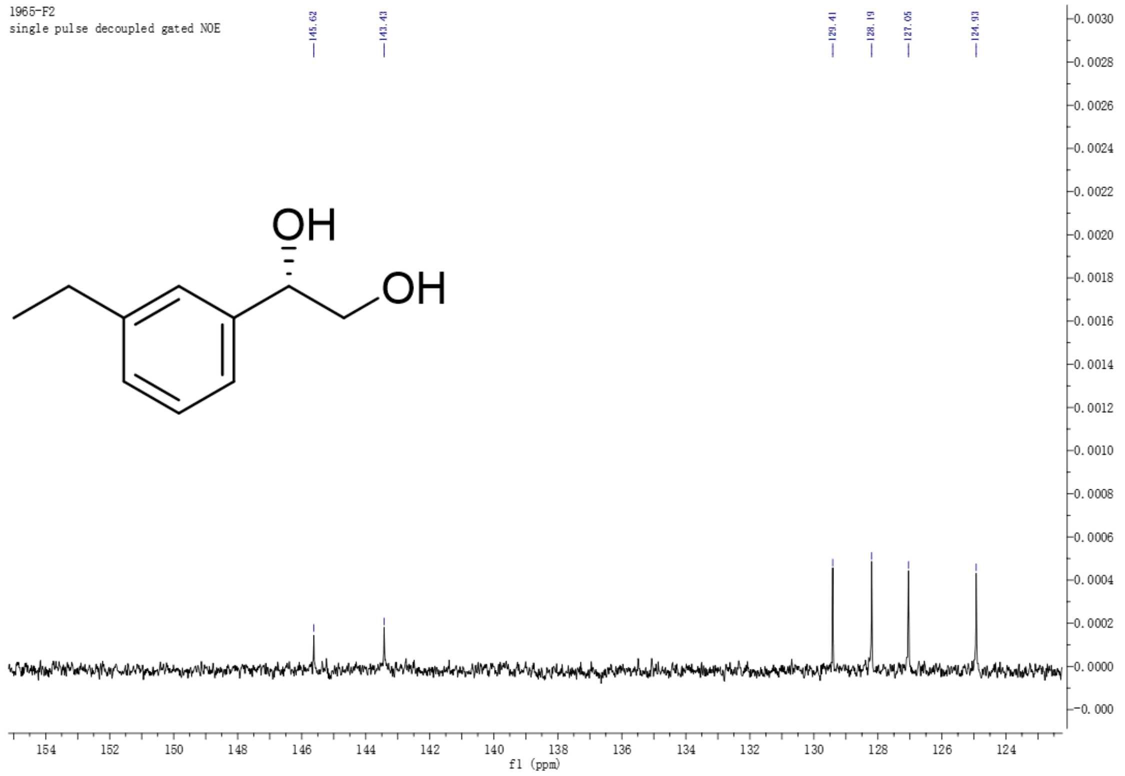

Figure S37. ^{13}C NMR spectrum of (*S*)-1-(3-ethylphenyl)-1,2-ethanediol (**4**)

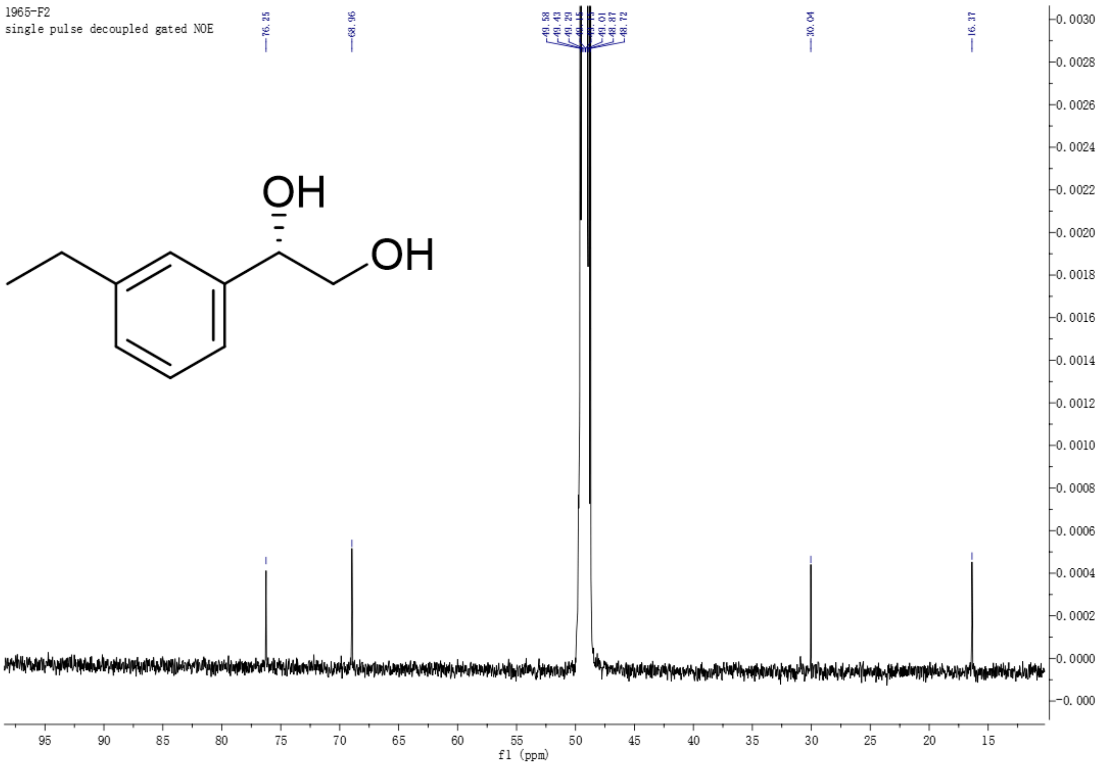

Figure S38. HRESIMS spectrum of (*S*)-1-(3-ethylphenyl)-1,2-ethanediol (**4**)

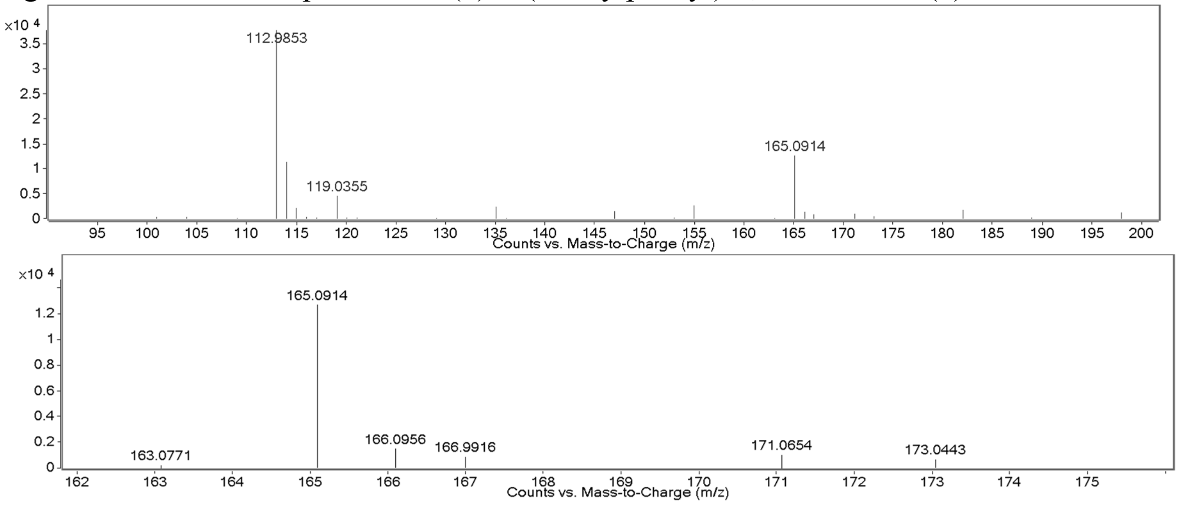

[M–H]⁻ 165.0914 (calcd for $C_{10}H_{13}O_2$, 165.0916)

Table S19. Crystal data and structure refinement parameters of streptodiketopiperazines A (**2**) and B (**3**)

Empirical formula	$C_{13}H_{14}N_2O_2$
Formula weight	230.26
Temperature/K	100.00(10)
Crystal system	monoclinic
Space group	$P2_1/c$
a/Å	12.0238(12)
b/Å	7.8159(7)
c/Å	12.0101(11)
α/°	90
β/°	97.506(9)
γ/°	90
Volume/Å^3	1119.00(18)
Z	4
ρ_{calc}g/cm^3	1.367
μ/mm^{-1}	0.094
F(000)	488.0
Crystal size/mm^3	0.14 × 0.13 × 0.12
Radiation	Mo Kα (λ = 0.71073)
2Θ range for data collection/°	6.234 to 49.998
Index ranges	$-9 \leq h \leq 14$, $-9 \leq k \leq 7$, $-14 \leq l \leq 14$
Reflections collected	4429
Independent reflections	1967 [R_{int} = 0.0336, R_{sigma} = 0.0530]
Data/restraints/parameters	1967/0/164
Goodness-of-fit on F^2	1.055
Final R indexes [I>=2σ (I)]	R_1 = 0.0458, wR_2 = 0.0932
Final R indexes [all data]	R_1 = 0.0611, wR_2 = 0.1030
Largest diff. peak/hole / e Å^{-3}	0.23/-0.24
CCDC Number	2004226
